# Causal association of dietary factors with five common cancers: univariate and multivariate Mendelian randomization studies

**DOI:** 10.3389/fnut.2024.1428844

**Published:** 2024-07-29

**Authors:** Lin Yang, Li Wang, Erhao Bao, Jiahao Wang, Pingyu Zhu

**Affiliations:** ^1^Department of Urology, Affiliated Hospital of North Sichuan Medical College, Nanchong, China; ^2^Department of Urology, The Second Hospital of Lanzhou University, Lanzhou, China; ^3^Department of Urology, The First People's Hospital of Dazhou, Dazhou, Sichuan, China; ^4^Department of Urology, People's Hospital of Xichong County, Nanchong, Sichuan, China

**Keywords:** consumption, dietary habit, cancer, Mendelian randomization, prognostic impact

## Abstract

**Background:**

Daily dietary habits are closely related to human health, and long-term unhealthy dietary intake, such as excessive consumption of alcohol and pickled foods, may promote the development of cancers. However, comprehensive research on the causal relationship between dietary habits and cancer is lacking. Therefore, this study aimed to reveal the potential causal link between dietary risk factors and the prognosis of cancer-related to genetic susceptibility.

**Methods:**

GWAS (Genome-Wide Association Studies) summary data on dietary habits and five common types of cancer and their pathological subtypes were obtained from the UK Biobank and various cancer association consortia. A univariable two-sample Mendelian randomization (UVMR) and FDR correction analysis was conducted to explore the causal relationships between 45 dietary habits and five common types of cancer and their histopathological subtypes. In addition, multivariable Mendelian randomization analysis (MVMR) was performed to adjust for traditional risk factors for dietary habits, and the direct or indirect effects of diet on cancer were evaluated. Finally, the prognostic impact of selected instrumental variables on cancer was analyzed using an online data platform.

**Results:**

In the UVMR analysis, four dietary habits were identified as risk factors for cancer, while five dietary habits were identified as protective factors. Among the latter, one dietary habit showed a significant association with cancer even after FDR correction, indicating a potential causal relationship. The MVMR analysis revealed that weekly beer and cider intake, may act as an independent risk factor for cancer development. Other causal associations between dietary habits and cancer risk may be mediated by intermediate factors. In the prognostic analysis, the SNPs (Single Nucleotide Polymorphisms) of average weekly beer and cider intake were set as independent risk factors and were found to significantly impact overall survival (OS) and cancer-specific survival (CSS) in lung cancer.

**Conclusion:**

This causal relationship study supports the notion that adjusting daily dietary habits and specific dietary interventions may decrease the risk of cancer.

## 1 Introduction

Cancer is a serious disease and current treatment modalities impose significant challenges. Overall, increasing mortality and incidence rates have been recorded worldwide, making cancer one of the leading causes of death (1). According to statistics from the American Cancer Society, prostate cancer (PC) and lung cancer (LC) are the most common malignancies in males, while breast cancer accounts for 31% of female cancers (2). The incidence rates of common gynecological malignancies, including endometrial and ovarian cancers, have also been on the rise over the years (3, 4). Lifestyle behaviors and metabolic factors such as dietary risks, alcohol consumption, and high fasting blood glucose are closely associated with the development and progression of cancers (5). In the tumor microenvironment, cancer cells involve complex metabolic pathways and show distinct characteristics from normal cells (6). Meanwhile, dietary habits, as an integral part of daily life, have a long-term impact on human health and metabolism. Therefore, dietary interventions and restrictions targeting metabolic dependencies of cancer cell seem to play a crucial role in cancer occurrence (7). A study reported a negative correlation between high adherence to the EAT-Lancet reference diet and the risk of cancer incidence and all-cause mortality (8). Conversely, a large prospective cohort study revealed a higher risk of developing cancer and other chronic metabolic diseases, such as cardiovascular disease, in individuals consuming a relatively unhealthy ultra-processed food diet (9). On the other hand, imbalanced nutritional intake, such as inadequate micronutrient levels and high-fat diets, can lead to obesity (10, 11). Moreover, researches have confirmed that high body weight index and obesity are significant risk factors for cancer development (12, 13). The mechanisms may involve obesity-induced inflammation and impaired anti-tumor immune surveillance, as well as abnormal lipid metabolism and changes in signaling that disrupt homeostasis and enhance cellular stress (14–16). However, existing research on the causal relationship between dietary habits and cancer has not yielded definitive conclusions.

This study employed Mendelian randomization (MR), which is a genetic instrumental variable analysis method (17). Inspired by Mendel’s second law of genetics, which shares similarities with the principles of randomized controlled trials (RCTs), our study design effectively minimized confounding factors and bias compared to prior clinical research approaches, explicitly making causal inferences (18). In this paper, PC, LC, endometrial cancer (EC), breast cancer (BC), and ovarian cancer (OC), along with their respective histopathological subtypes, were selected as the outcome variables. The causal relationships and related prognostic implications between 45 dietary habits and these five common malignancies were systematically investigated, aiming to facilitate a comprehensive understanding of the impact of diet on cancer progression and the identification of valuable dietary interventions for cancer prevention.

## 2 Methods

### 2.1 Study design and data access

**Figure 1:**
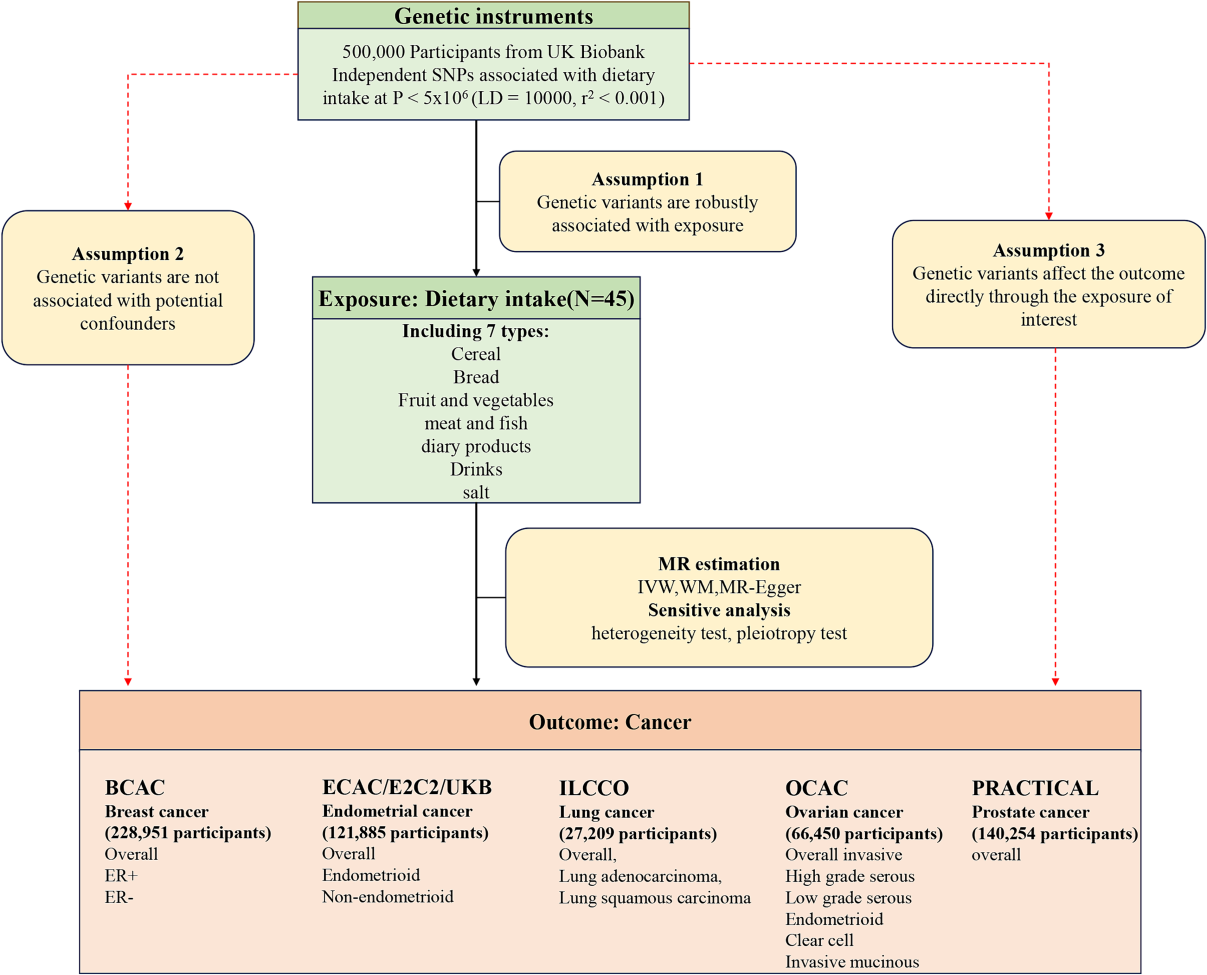
Schematic of the study design.

Figure 1 provides an overview of the study design. The univariable Mendelian randomization (MR) approach was employed to evaluate the causal effects of 45 dietary habits on the occurrence of five common cancers and their subtypes. The multivariable adjustment was performed by including traditional risk factors for cancer that were positively associated with the outcome risk, and the potential mediation effects were examined. Summary data for dietary habits can be found in Supplementary Table S1, which was sourced from the UK Biobank, including information on lifestyle and physical health from approximately 500,000 participants (19). The outcome data were obtained from large-scale GWAS (Genome-Wide Association Studies) conducted by the Breast Cancer Association Consortium (BCAC), Endometrial Cancer Association Consortium (ECAC), Endometrial Cancer Epidemiology Consortium (E2C2), UK Biobank, International Lung Cancer Consortium (ILCCO), Ovarian Cancer Association Consortium (OCAC), and Prostate Cancer Association Group to Investigate Cancer Associated Alterations in the Genome (PRACTICAL) consortium. The summary information for breast cancer (BC) and its subtypes included 122,977 cases and 105,974 controls, with estrogen receptor-positive (ER+) and estrogen receptor-negative (ER−) breast cancer included in the MR subgroup analysis (20). The GWAS data for endometrial cancer (EC) included the two subtypes endometrioid and non-endometrioid cancer, with 12,906 cancer patients and 108,979 healthy people (21). The summary GWAS data for lung cancer (LC) and its subtypes [lung squamous cell carcinoma (LUSC) and lung adenocarcinoma (LUAD)] involved 11,348 cases and 15,861 controls (22). Subgroup analysis was conducted on five histological subtypes of ovarian cancer (OC) using GWAS summary data, including high-grade serous (HGS), low-grade serous (LGS), endometrioid (ED), clear cell (CC), and invasive mucinous (IM) subtypes, with 25,509 cases and 40,941 controls (23). Cancer genetic information was extracted from the GWAS statistical data for prostate cancer, involving 79,148 diagnosed cases and 61,106 healthy controls (24). Additionally, summary data from genome-wide association studies (GWAS) were used to collect information about conventional risk factors of common cancers for multivariable analysis, including body mass index (BMI), age at menarche, age at menopause, and smoking (25–29). The aforementioned summary data can be obtained from the open GWAS in the IEU (Integrative Epidemiology Unit) program at https://gwas.mrcieu.ac.uk. The detailed sources of GWAS data for cancer and traditional risk factors can be found in Table 1.

**Table 1:** Description of GWAS data sources for each phenotype.

Dataset type	Variable	GWAS ID	Sample size	Consortium	Population	Sex
Exposure	Dietary habits	See Supplementary Table S1		MRC-IEU	European	Males and females
	Body mass index	ieu-a-2	339,224	GIANT	European	Males and females
	Age at menopause	ieu-a-1004	69,360	ReproGen	European	Females
	Age at menarche	ieu-a-1095	182,416	ReproGen	European	Females
	Age of Smoking Initiation	ieu-b-24	341,427	GSCAN	European	Males and females
Outcome	BC, breast cancer	ieu-a-1126	228,951	BCAC	European	Females
	BC (ER+), estrogen receptor positive breast cancer	ieu-a-1127	175,475	BCAC	European	Females
	BC (ER−), estrogen receptor negative breast cancer	ieu-a-1128	127,442	BCAC	European	Females
	EC, endometrial cancer	ebi-a-GCST006464	121,885	ECAC/E2C2/UKB	European	Females
	EC (EH), endometrial cancer (endometrioid histology)	ebi-a-GCST006465	54,884	ECAC/E2C2/UKB	European	Females
	EC (EH), endometrial cancer (non-endometrioid histology)	ebi-a-GCST006466	36,677	ECAC/E2C2/UKB	European	Females
	LC, lung cancer	ieu-a−966	27,209	ILCCO	European	Males and females
	LUAD, lung adenocarcinoma	ieu-a-965	18,336	ILCCO	European	Males and females
	LUSC, lung squamous carcinoma	ieu-a-967	18,313	ILCCO	European	Males and females
	OC, ovarian cancer	ieu-a−1,120	66,450	OCAC	European	Females
	OC (HGS), high grade serous ovarian cancer	ieu-a-1121	53,978	OCAC	European	Females
	OC (LGS), low grade serous ovarian cancer	ieu-a-1122	41,953	OCAC	European	Females
	OC (IM), invasive mucinous ovarian cancer	ieu-a-1123	42,358	OCAC	European	Females
	OC (CC), invasive mucinous ovarian cancer	ieu-a-1124	42,307	OCAC	European	Females
	OC (ED), endometrioid ovarian cancer	ieu-a-1125	43,751	OCAC	European	Females
	PC, prostate cancer	ieu-b-85	140,254	PRACTICAL	European	Males

### 2.2 Selection of instrumental variables

Single nucleotide polymorphisms (SNPs) represent genetic diversity at the DNA sequence level and are characterized by variations in a single nucleotide. In this study, SNPs (including coding-region, intergenic, and peri-genetic locations) were used as instrumental variables for causal inference, with SNPs affecting the phenotype including synonymous coding SNPs and nonsynonymous coding SNPs (missense and nonsense mutations). The threshold for filtering instrumental variables was set at *p* < 1 × 10^−6^ to ensure a sufficiently large SNP number for analysis. The linkage disequilibrium parameter was set at *r*^2^ = 0.001, with a cluster window of 10,000 kb, ensuring independence between SNPs (30). Weak instrumental variables with an *F*-statistic <10 were excluded. Finally, a harmonization step was performed for the effect alleles, removing non-matching alleles and alleles with palindromic sequences (31).

### 2.3 Statistical analysis

The inverse variance-weighted method (IVW) was employed as the primary approach for causal inference in both univariate (UVMR) and multivariate (MVMR) Mendelian randomization analyses. The dietary-related GWAS data and outcome data from different cancer consortia may differ in terms of data collection methods and other factors. Therefore, to ensure comparability, a homogeneous population (Europeans) was selected for our analysis. Besides, a series of sensitivity analyses were conducted to comprehensively assess heterogeneity and potential differences in the findings. Firstly, the heterogeneity of the data was evaluated using the Cochran *Q* test. A *p*-value of less than 0.05 indicated the presence of heterogeneity, and the IVW random effects model was applied to mitigate the potential impact. Conversely, a *p*-value >0.05 suggested no heterogeneity, and a fixed effects model was used. Subsequently, causal associations were determined (32). Moreover, alternative statistical methods were employed, including weight median, MR-Egger, and MR-PRESSO. The weight median approach provides robust causal effect estimates even in the presence of multiple invalid instruments (33). The MR-Egger regression analysis estimates the causal effects for all included instruments and conducts directional pleiotropy tests to determine the presence of horizontal pleiotropy (34). On the other hand, MR-PRESSO identifies and checks for outliers and horizontal pleiotropy, thereby enhancing the robustness of the results (35). Furthermore, the false discovery rate (FDR) method was applied to correct the *p*-values of the univariate analysis results (a *p*-value of FDR < 0.05 indicates statistical significance), enhancing the reliability of positive findings (36). Lastly, multivariable analysis adjustments were adopted to assess whether the association between dietary intake habits, traditional risk factors, and a high risk of cancer potential were mediated by confounding factors. In addition, the MVMR approach extends the single-variable Mendelian randomization (SMR) by leveraging genetic variants that may be associated with multiple exposures, including dietary habits and common risk factors for malignant tumors. This method estimates the impact of each exposure on the same outcome. Furthermore, MVMR enables the analysis of multiple exposures in an equivalent manner, facilitating the estimation of the direct effect (the effect of exposure on the outcome solely through the exposure) and potential mediating effect (the effect of exposure through traditional risk factors as mediating variables on outcomes) (29). Statistical analyses were performed using R software version 4.3.0 with the following packages: “TwoSampleMR,” “MendelianRandomization,” and “MR-PRESSO.” The STROBE-MR checklist for this study is presented in Supplementary Table S3.

### 2.4 Prognostic evaluation of cancers by instrumental variables

The online platform (SUMMER, http://njmu-edu.cn:3838/SUMMER/) was used to evaluate the prognostic impact of genetic variants on corresponding cancer survival included overall survival (OS) and cancer-specific survival (CSS), additionally utilizing the results to draw Kaplan–Meier curves (37).

## 3 Results

### 3.1 UVMR analyzes the potential impact of 45 dietary habits on five common cancers

**Figure 2:**
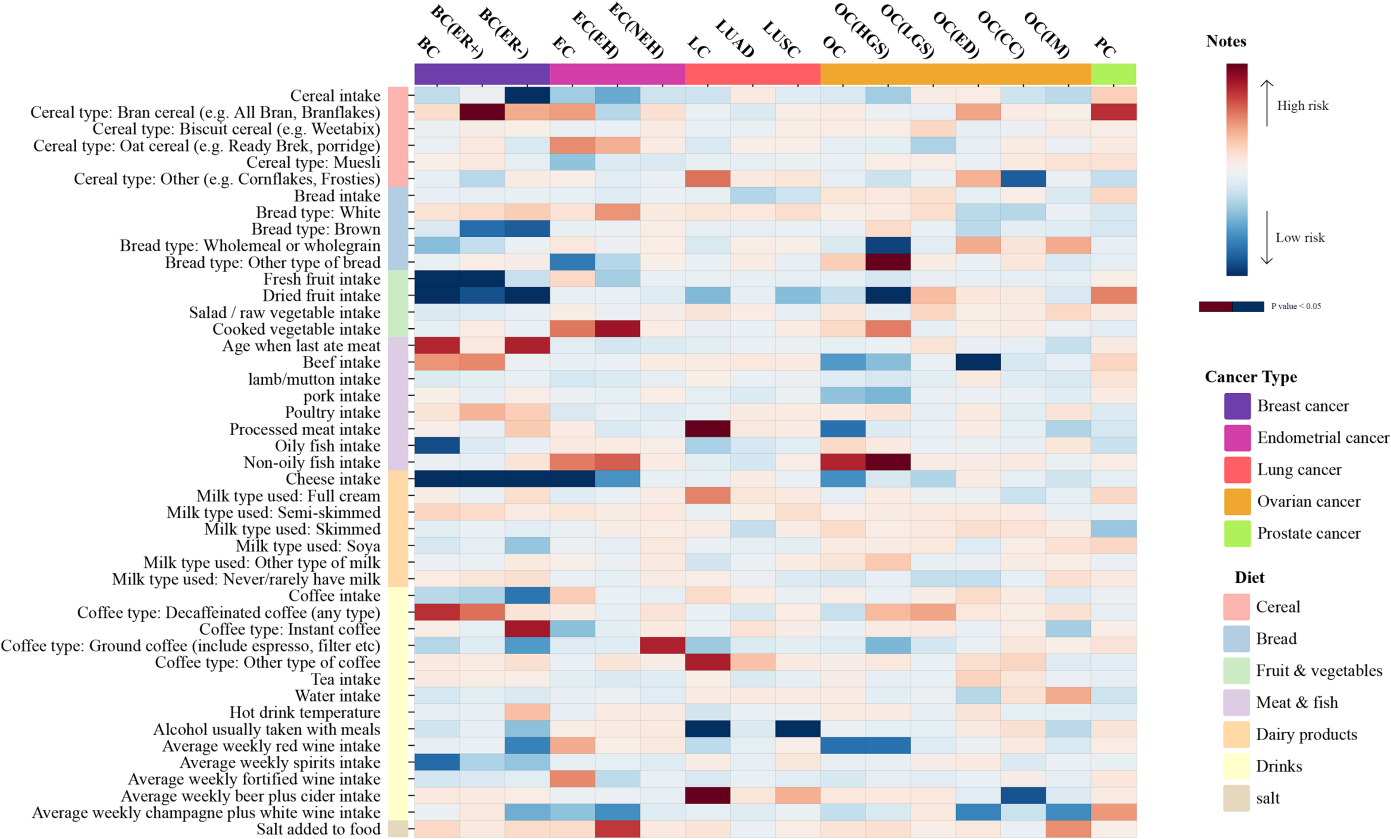
The heatmap illustrates the causal relationships between various dietary intakes and five common cancers determined by the IVW method. The 45 dietary habits and five cancers are categorized respectively. Dietary categories include cereal, bread, fruit and vegetables, meat and fish, dairy products, drinks, and salt. The cancer types comprise breast cancer, endometrial cancer, lung cancer, ovarian cancer, and prostate cancer. The greater the significance (*P-*value < 0.05), the deeper the color, with blue indicating protective factors and red risk factors.

Details of the extracted SNP information are provided in Supplementary Tables S29–S44. A univariate analysis was performed to investigate the potential causality between 45 common dietary habits and five common types of malignant tumors and their subtypes. Furthermore, FDR correction analysis was carried out on all *p*-values of the output results, as described in Figure 2 and Supplementary Tables S2–S9, S26. Sensitivity analysis models were employed to examine the robustness of the output results (Supplementary Tables S10–S24). The results identified 16 dietary intake habits with potential causal associations with common cancers. Among them, four dietary intake habits were positively associated with an increased risk of the corresponding types of cancer, while five dietary intake habits were negatively associated with the occurrence of different carcinomas. FDR correction was performed on the *p*-values of the IVW results with potential associations, revealing only two corrected positive results, which were defined as reliable positive evidence. In the analysis of the association between breast cancer (BC) and dietary intake habits, three protective factors were identified, including dried fruit (OR = 0.803, 95%CI: 0.684–0.943, *p* = 0.007), fresh fruit (OR = 0.779, 95%CI: 0.640–0.949, *p* = 0.013), and cheese (OR = 0.885, 95%CI: 0.799–0.981, *p* = 0.020). BC (ER+) is a subtype of BC, and fresh fruit (OR = 0.79, 95%CI: 0.641–0.972, *p* = 0.026) and cheese (OR = 0.881, 95%CI: 0.787–0.987, *p* = 0.029) can reduce the risk of its occurrence, while bran cereal (OR = 2.927, 95%CI: 1.052–8.142, *p* = 0.04) increases the risk of BC (ER+). As another subtype of breast cancer, BC (ER−) showed potential associations with three dietary intake habits; among these, cheese (OR = 0.784, 95%CI: 0.676–0.91, *p* = 0.001, FDR = 0.030165) was the only reliable positive evidence and acted as a protective factor against BC (ER−). The other positive results that were excluded by FDR correction comprised dried fruit (OR = 0.727, 95%CI: 0.58–0.91, *p* = 0.005) and cereal (OR = 0.768, 95%CI: 0.634–0.929, *p* = 0.006), which also showed negative correlations with the risk of BC (ER−). Additionally, cheese (OR = 0.705, 95%CI: 0.545–0.914, *p* = 0.008) was the only dietary intake habit associated with endometrial cancer (EC) and acted as a protective factor. Moreover, two dietary habits were positively associated with the risk of malignant tumors of the lung, including beer and cider intake (OR = 1.741, 95%CI: 1.095–2.768, *p* = 0.019) and processed meat intake (OR = 1.474, 95%CI: 1.045–2.079, *p* = 0.027). However, alcohol taken with meals (OR = 0.45, 95%CI: 0.286–0.709, *p* < 0.001, FDR = 0.025762) was identified as reliable positive evidence and was related to a lower risk of lung cancer. Similarly, alcohol taken with meals (OR = 0.461, 95%CI: 0.223–0.957, *p* = 0.038) also showed an inverse correlation with the risk of the lung cancer subtype LUSC. The risk of ovarian cancer (HGS) was found to be associated with two dietary intake habits: non-oily fish (OR = 0.746, 95%CI: 0.557–0.999, *p* = 0.049) as a risk factor and dried fruit (OR = 1.635, 95%CI: 1.001–2.672, *p* = 0.049) as a protective factor. The list of positive results is detailed in Table 2.

**Table 2:** Significant and nominal significant MR results of FDR correction.

Outcome	Exposure	SNP(n)	IVW			WM		MR-Egger	
			OR (95%CI)	*p*-value	FDR	OR (95%CI)	*P*-value	Intercept	*P*-value
BC	Dried fruit intake	149	0.80(0.68,0.94)	7.66E-03	2.31E-01	0.76(0.63,0.90)	2.20E-03	2.12E-03	5.40E-01
BC	Fresh fruit intake	134	0.78(0.64,0.95)	1.34E-02	2.31E-01	0.80(0.61,1.04)	9.40E-02	-5.36E-05	9.85E-01
BC	Cheese intake	187	0.89(0.80,0.98)	2.05E-02	2.31E-01	0.86(0.76,0.97)	1.53E-02	1.16E-03	6.71E-01
BC (ER+)	Fresh fruit intake	133	0.79(0.64,0.97)	2.61E-02	5.86E-01	0.76(0.55,1.05)	1.30E-01	2.60E-03	3.76E-01
BC (ER+)	Cheese intake	184	0.88(0.79,0.99)	2.88E-02	5.86E-01	0.90(0.78,1.03)	9.85E-02	−4.10E-04	8.91E-01
BC (ER+)	Cereal type: Bran cereal (e.g., All Bran, Branflakes)	11	2.93(1.05,8.14)	3.96E-02	5.86E-01	1.87(0.49,7.20)	3.61E-01	-8.00E-04	9.23E-01
BC (ER−)	Cheese intake	186	0.78(0.68,0.91)	1.34E-03	3.02E-02	0.91(0.74,1.12)	3.82E-01	3.16E-04	9.37E-01
BC (ER−)	Dried fruit intake	150	0.73(0.58,0.91)	5.38E-03	6.49E-02	0.70(0.51,0.95)	2.27E-02	−1.89E-03	6.90E-01
BC (ER−)	Cereal intake	165	0.77(0.63,0.93)	6.46E-03	6.49E-02	0.78(0.59,1.03)	8.46E-02	-1.48E-03	7.12E-01
EC (EH)	Cheese intake	203	0.71(0.54,0.91)	8.21E-03	1.85E-01	0.54(0.38,0.76)	4.62E-04	-9.71E-03	1.70E-01
LC	Alcohol usually taken with meals	130	0.45(0.29,0.71)	5.72E-04	2.58E-02	0.42(0.22,0.80)	7.73E-03	-1.04E-02	1.93E-01
LC	Average weekly beer plus cider intake	73	1.74(1.09,2.77)	1.92E-02	4.08E-01	1.71(0.94,3.09)	7.79E-02	-1.40E-02	1.37E-01
LC	Processed meat intake	88	1.47(1.04,2.08)	2.72E-02	4.08E-01	1.81(1.13,2.89)	1.29E-02	-4.48E-03	6.33E-01
LUSC	Alcohol usually taken with meals	119	0.46(0.22,0.96)	3.77E-02	9.27E-01	0.42(0.15,1.17)	9.66E-02	-1.53E-02	2.42E-01
OC (HGS)	Dried fruit intake	144	0.75(0.56,1.00)	4.94E-02	5.97E-01	0.84(0.55,1.28)	4.16E-01	4.53E-04	9.41E-01
OC (HGS)	Non-oily fish intake	57	1.64(1.00,2.67)	4.94E-02	5.97E-01	2.39(1.19,4.82)	1.48E-02	−3.69E-03	6.54E-01

### 3.2 MVMR searches for potential mediating confounders

**Figure 3:**
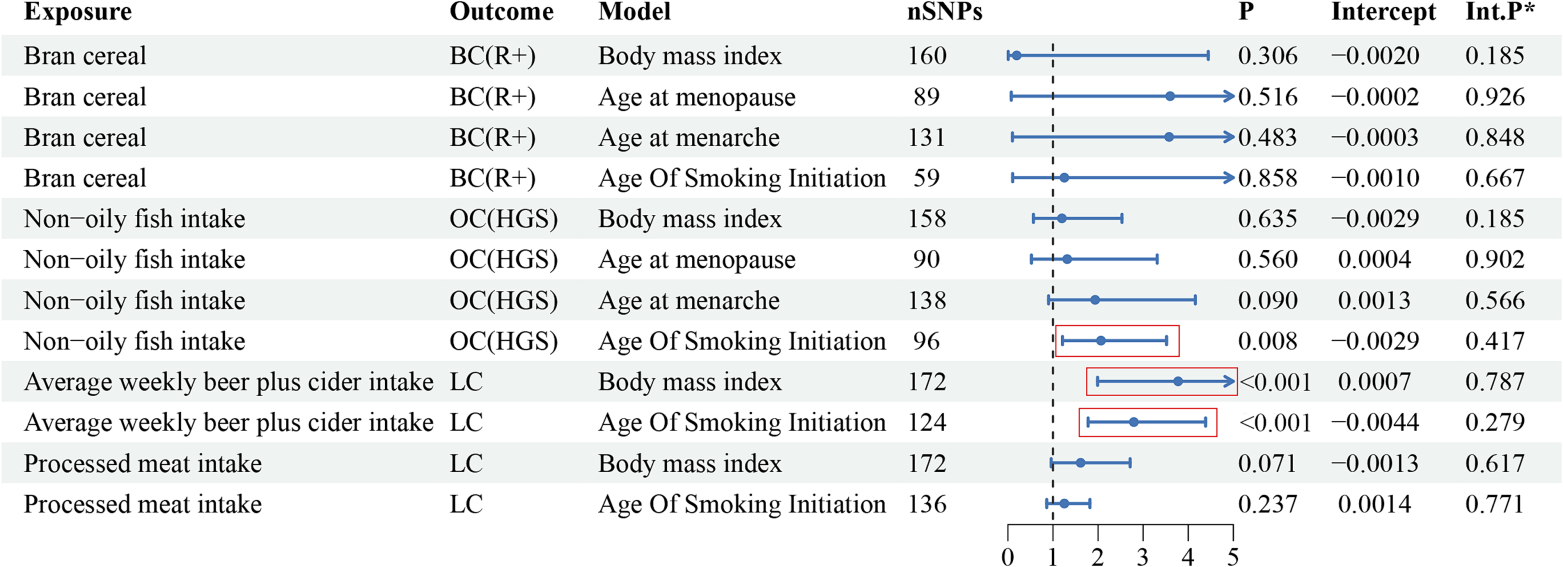
The multivariate analysis results of risk factor diets with traditional risk factor adjustment.

Figure 3 presents the multivariate analysis results. The findings revealed four eating habits that were linked to an increased risk of developing specific cancers. Therefore, multivariate analysis was conducted to determine whether these associations were direct risk factors for cancer occurrence or mediated through traditional risk factors (BMI, age at menarche, age at menopause, smoking). The detailed data can be found in Supplementary Table S27. After adjusting for the four traditional risk factors, the association between breast cancer and bran cereal intake became non-significant, suggesting that other factors may potentially mediate the impact of this dietary habit on the risk of breast cancer. Similarly, the causal relationship between lung cancer and processed meat consumption was not significant after adjusting for BMI and smoking, indicating that the effect of processed meat consumption on lung cancer risk may be mediated by factors such as BMI and smoking. However, the association between lung cancer and average weekly beer and cider intake remained significant even after incorporating BMI and smoking into the multivariate models. Moreover, after adjusting for smoking as a covariate, the relationship between non-oily fish intake and HGS remained significant; in contrast, the output results after adjusting for the other three variables did not show significance. This may suggest that BMI, age at menarche, and age at menopause may mediate the causal relationship between non-oily fish intake and ovarian cancer. Additionally, none of the *p*-values from the multivariate Egger intercept tests demonstrated statistical significance, indicating the absence of horizontal pleiotropy.

### 3.3 Influence of dietary intake habits on the prognosis of five common cancers

**Figure 4:**
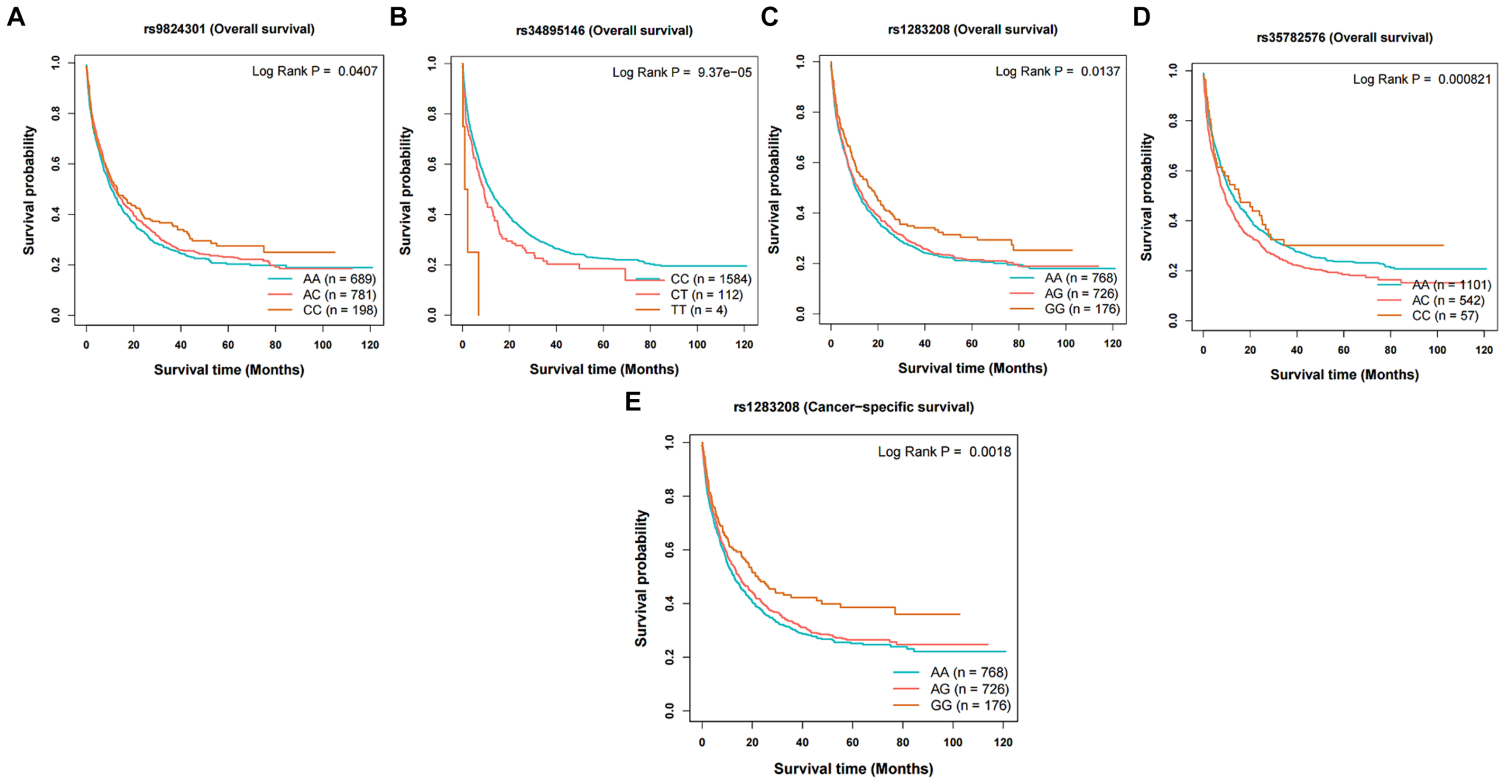
Kaplan–Meier plots of the effect of the independent risk factor on prognosis for survival in lung cancer. Association between variants **(A)** rs9824301, **(B)** rs34895146, **(C)** rs1283208, and **(D)** rs35782576 and overall survival in lung cancer. Association between variants **(E)** rs1283208 and cancer-specific survival in lung cancer.

The instrumental variables representing dietary intake habits that could independently influence the risk of cancer were screened and evaluated. The selected SNPs may have an impact on the occurrence and prognosis (overall survival and cancer-specific survival) of the corresponding cancers (Figure 4 and Supplementary Table S28). The instrumental variables related to average weekly beer and cider intake, including rs9824301 (HR: 0.91, *p* = 0.047) and rs1283208 (HR: 0.91, *p* = 0.033), were positively correlated with longer overall survival (OS) in lung cancer (LC). In addition, rs1283208 (HR: 0.88, *p* = 0.006) was significantly associated with longer cancer-specific survival (CSS). On the other hand, rs34895146 (HR: 1.29, *p* = 0.014) and rs35782576 (HR: 1.10, *p* = 0.042) had the opposite effect on OS in LC.

## 4 Discussion

This study investigated the causal relationship between 45 dietary intake habits and 5 common types of cancer and their subtypes. Previous research has explored the potential connections between diet and specific types of cancer, but a systematic and comprehensive examination of whether there is a definitive causal relationship between diet and common cancers remains lacking. The present study was designed to fill this gap. Dietary intake directly passes through the gastrointestinal tract and is closely related to the digestive system, with early research often focusing on diet and gastrointestinal tumors. Therefore, our study primarily investigated five common malignant tumors outside of the gastrointestinal tract.

### 4.1 Main findings and implications for public health

In this study, the causal correlations between 45 dietary habits and five prevalent types of cancer and their histological subtypes were investigated. A total of 16 potential causal relationships were identified. Among them, five dietary habits were associated with a lower risk of developing multiple cancers, including cheese, dried fruits, fresh fruits, cereal, and alcohol taken with meals. A significant positive connection was observed between four dietary habits and the risk of developing corresponding cancers. Specifically, the consumption of bran cereal, average weekly beer and cider, processed meat, and non-oily fish was found to be significantly related to an increased risk of these cancers. Furthermore, a multivariable analysis was conducted after adjusting for traditional risk factors of cancer; the results indicated the potential independent risk factors. Our findings revealed that beer and cider possibly becomes an independent potential risk of cancer occurrence. In contrast, the other three causal relationships identified in our study may be influenced by intermediate factors, indicating potential confounding. Moreover, as dietary factors are important lifestyle components, they may also impact the prognosis of cancer patients (38–41). By selecting tool variables from an independent risk factor (weekly consumption of beer and cider), Kaplan–Meier curves were plotted to provide a more intuitive representation of the prognostic impact. From a medical and public health standpoint, uncovering dietary patterns that may impact cancer risk provides the information necessary to craft tailored dietary recommendations designed to lower the prevalence of these diseases. Ensuring a sufficient intake of fresh fruits, and grains, and restricting the consumption of processed meats, as well as beer mixed with apple cider vinegar, could form an integral part of a multifaceted approach to cancer prevention. Furthermore, the application of pertinent instrumental variable methods to investigate the potential causal influences of dietary risk elements on cancer prognoses not only enhances our comprehension of the health implications of dietary practices but also provides a more refined insight into these relationships.

### 4.2 Comparison with other reports

In the current study, cheese was found to have a favorable protective effect on the risk of breast cancer and its two histological subcategories, endometrioid and serous cancers. A previous prospective cohort study revealed an association between long-term and premenopausal cheese intake and a lower risk of breast cancer (42). Another systematic review and meta-analysis demonstrated that cheese consumption was associated with lower overall mortality from various chronic diseases; however, no significant association was found between breast cancer and endometrial cancer (43). Additionally, previous studies did not support a correlation between cheese and endometrial cancer (44–46). Jin et al. (47) conducted a Mendelian randomization analysis and discovered that each elevation in dried fruit intake of one standard deviation was linked to a substantial reduction in the risk of developing cancer (excluding prostate cancer, bladder cancer, and brain cancer), ranging from 0.53 to 97.26%. These results were consistent with our findings, although the present study only found a positive association between dried fruit intake and breast cancer (including ER-negative breast cancer) and ovarian cancer. Furthermore, fresh fruit and cereal also demonstrated a negative causal relationship with the risk of breast cancer, which was supported by an MR analysis of breast cancer (48) and a systematic review exploring the correlations between dietary fiber consumption and cancer risk (49, 50). Nonetheless, our results indicated a positive association between bran cereal intake and the risk of breast cancer, which was contrary to the early research by (51) who reported that increasing bran cereal intake could somewhat reduce the risk of breast cancer. A study exploring the association between lung cancer and behavioral characteristics confirmed that weekly beer and cider intake could increase genetic susceptibility to lung cancer, while alcohol usually taken with meals had the opposite effect (52), which aligned with our findings. The latest meta-analysis on meat intake and cancer risk yielded similar results, indicating that processed meat consumption could increase the risk of lung cancer by 12% (53). Two prospective cohort studies on oily fish and non-oily fish consistently found that both types of fish could reduce overall mortality from cancer (54, 55). However, the present study yielded contrasting results: non-oily fish was significantly associated with a higher risk of ovarian cancer, whereas no significant causal relationship was found between oily fish and cancer. It is noteworthy that a limited number of previous studies have explored the correlation between genetic variants linked to dietary factors and cancer (56–59), but the nature of this association remains ambiguous. Our study’s findings possibly offer an opportunity to further validate and elucidate previously reported correlations.

### 4.3 Possible mechanisms of action

Next, the mechanisms by which food influences cancer were explored. Cheese is rich in nutrients, including proteins, minerals, and vitamins (60). Research revealed that cheese contains various bioactive peptides in casein, such as αS1-casein and β-casein, which exert anticancer effects. Other peptides in cheese also have antioxidant and immune-regulating activities, which indirectly contribute to the prevention of cancer and other chronic diseases (60, 61). Saffron, used in the production of cheese, contains bioactive compounds such as crocin, picrocrocin, and safranal, which have been found to effectively inhibit the proliferation of cancer cells, including cervical cancer, breast cancer, and leukemia (62). Walnuts are a common type of dried fruit and animal studies have shown that the melatonin and polyunsaturated fatty acids present in walnuts synergistically regulate the activity of cyclooxygenase and lipoxygenase in fatty acid metabolism, thereby inhibiting the growth, invasion, and metastasis of tumors in breast cancer mouse models (63). Fresh fruits contain beta-carotene, riboflavin, and vitamin C, and have been negatively associated with the risk of breast cancer, as mentioned in a review article (64). In addition, plant polyphenols found in fresh fruits, such as resveratrol, can inhibit signaling pathways like Wnt/β-catenin, induce autophagy, and suppress proliferation in breast cancer cells (65). Flavonoids found in citrus fruits have a similar structure to estrogen and can bind to estrogen receptors (ER), inhibiting the proliferation of estrogen-dependent breast cancer cells (66). Moreover, cereals also contain a large amount of bioactive phenolic compounds (67), and several studies have reported the anticancer activity of β-sitosterol, which is found in cereals and grains (68–71), and its promising applications in the treatment of breast cancer (72–75). The mechanism of action of β-SDG may involve the upregulation of the tumor suppressor gene miR-10a and the inactivation of the PI3K-Akt signaling pathway, leading to cell cycle arrest at the G0/G1 phase and inhibition of tumor growth and proliferation (70, 71). However, intriguingly, our study revealed that bran cereal had the opposite effect and lacked corresponding research to explain this phenomenon. The relationship between alcohol and lung cancer seems to be well-established (28). In the present study, after multivariable adjustment, alcohol emerged as an independent risk factor for lung cancer. However, the association was limited to beer and cider, and no significant association was found with other types of alcohol. Interestingly, the outcome of our study demonstrated a link between the consumption of alcohol with meals and a decreased likelihood of developing lung carcinoma. A genome-wide association study on drinking habits revealed that alcohol taken with meals was not genetically correlated with problematic alcohol use (PAU), a condition associated with alcohol-related diseases, compared to the maximum habitual alcohol intake (76). Furthermore, processed meat refers to meat products that have undergone processes such as curing to enhance flavor or extend shelf life (77). Processed meat products contain carcinogenic substances such as nitrites and heterocyclic aromatic amines, which can promote tumor development at multiple sites. Processed meat also contains high sodium and saturated fatty acid levels. On the other hand, our multivariable analysis indicated that BMI may mediate the effect of processed meat on lung cancer risk. Therefore, eating processed meat may indirectly affect lung cancer incidence through changes in BMI-related metabolic factors (78). Currently, a few studies have yielded negative reports on the intake of non-oily fish. After multivariable adjustment in our analysis, the relation between non-oily fish and ovarian carcinoma was no longer significant, suggesting the presence of other unidentified confounding factors in the research.

### 4.4 Strengths and limitations

A comprehensive and systematic study was conducted to explore the cause-and-effect connections between multiple eating habits and 5 common types of cancer, including their subtypes. This is a major strength of our study. Nonetheless, the limitations should be acknowledged. Firstly, the data were sourced from the UK Biobank, derived from individuals of European descent; since dietary habits show significant variation among different ethnicities and regions, our findings may not be applicable to other populations. Besides, the data may contain demographic errors, and the limited sample size inevitably results in selection bias, which may potentially affect the validity and reliability of the study’s findings. To enhance the generalizability of our findings, future research should aim to include a more diverse sample, drawing from collaborative studies and datasets from around the world, offering a broader racial sample and effectively reflecting global populations. A larger sample size is crucial for reliable and effective estimates. Future research should strive to expand the sample size to increase the statistical power to detect significant associations. Secondly, the menopausal status of women with breast, endometrial, and ovarian cancer was not stratified. However, traditional risk factors for these cancers were considered, including age at menarche and age at menopause. In addition, multivariable analysis was performed to assess the associations between dietary intake habits and these three cancers, considering the potential influence of menopausal status. Thirdly, FDR analysis was conducted to control for false positive events and only one statistically significant association was obtained after correction. However, this does not diminish the value of the positive causal associations, as they provide direction and insights for future scientific research. Furthermore, although our study did not delve into the reverse causal associations between diet and malignant tumors, it provided a direction for future research. For example, future research could employ a large-scale, longitudinal cohort design to track dietary habits and cancer development over time. This could involve investigating whether changes in nutritional habits precede cancer diagnoses or if cancer itself influences dietary choices. Finally, our study findings indicate that dietary habits have varying and sometimes opposite effects on different subtypes of the same cancer. These differences may be driven by unknown mechanisms related to biological markers. This could provide valuable insights for future clinical and basic research. In summary, by addressing these study limitations and exploring these research directions, future studies can build upon our findings to provide a more comprehensive understanding of the cause-and-effect relationships between dietary habits and cancers.

## 5 Conclusion

The connection between numerous dietary habits and five common types of cancer and their subtypes was assessed using Mendelian randomization (MR) analysis. Our study revealed potential causal associations between certain dietary intakes and corresponding cancers. Furthermore, beer and cider were found to directly influence the morbidity risk of lung carcinoma, which was independent of traditional risk factors. The study provides information to implement daily dietary habits as possible strategies for preventing cancer.

## Data availability statement

The datasets presented in this study can be found in online repositories. The names of the repository/repositories and accession number(s) can be found in the article/Supplementary material.

## Author contributions

LY: Conceptualization, Data curation, Methodology, Writing – original draft, Writing – review & editing. LW: Conceptualization, Supervision, Writing – review & editing. EB: Methodology, Writing – review & editing. JW: Software, Writing – review & editing. PZ: Writing – review & editing.
