# Supplementary material for: Causal association of dietary factors with five common cancers: univariate and multivariate Mendelian randomization studies
Source: Front Nutr. 2024 Jul 29;11:1428844. doi: 10.3389/fnut.2024.1428844 (PMC11317396; doi:10.3389/fnut.2024.1428844)
Supplement: Supplementary file 1 [file Table_1.docx]

**Supplementary Material**

**Table S1.** Data resources of 45 dietary habits.

| **Dietary habits** | **GWAS ID** | **Sample size** | **Types of diet** | **ACE touchscreen question** |
| --- | --- | --- | --- | --- |
| Cereal intake | ukb-b-15926 | 441,640 | Cereal | "How many bowls of cereal do you eat a WEEK?" |
| Cereal type: Bran cereal (e.g. All Bran, Branflakes) | ukb-d-1468_1 | 299,898 | Cereal | "What type of cereal do you mainly eat?"(If you eat more than one type of cereal, please select the one that you eat the most.) |
| Cereal type: Biscuit cereal (e.g. Weetabix) | ukb-d-1468_2 | 299,898 | Cereal |  |
| Cereal type: Oat cereal (e.g. Ready Brek, porridge) | ukb-d-1468_3 | 299,898 | Cereal |  |
| Cereal type: Muesli | ukb-d-1468_4 | 299,898 | Cereal |  |
| Cereal type: Other (e.g. Cornflakes, Frosties) | ukb-d-1468_5 | 299,898 | Cereal |  |
| Bread intake | ukb-b-11348 | 452,236 | Bread | "How many slices of bread do you eat each WEEK?" (For other types of bread: - one bread roll = 2 slices; - one pitta bread = 2 slices) |
| Bread type: White | ukb-d-1448_1 | 348,424 | Bread | "What type of bread do you mainly eat?"(If you eat more than one type of bread, please select the one that you eat the most.) |
| Bread type: Brown | ukb-d-1448_2 | 348,424 | Bread |  |
| Bread type: Wholemeal or wholegrain | ukb-d-1448_3 | 348,424 | Bread |  |
| Bread type: Other type of bread | ukb-d-1448_4 | 348,424 | Bread |  |
| Fresh fruit intake | ukb-b-3881 | 446,462 | Fruit and vegetables | "About how many pieces of FRESH fruit would you eat per DAY? (Count one apple, one banana, 10 grapes etc as one piece; put '0' if you do not eat any)" |
| Dried fruit intake | ukb-b-16576 | 421,764 | Fruit and vegetables | "About how many pieces of DRIED fruit would you eat per DAY? (Count one prune, one dried apricot, 10 raisins as one piece; put '0' if you do not eat any)" |
| Salad / raw vegetable intake | ukb-b-1996 | 435,435 | Fruit and vegetables | "On average how many heaped tablespoons of SALAD or RAW vegetables would you eat per DAY? (Include lettuce, tomato in sandwiches; put '0' if you do not eat any)" |
| Cooked vegetable intake | ukb-b-8089 | 448,651 | Fruit and vegetables | "On average how many heaped tablespoons of COOKED vegetables would you eat per DAY? (Do not include potatoes; put '0' if you do not eat any)" |
| Age when last ate meat | ukb-b-9791 | 17,236 | Meat and fish | "How old were you when you last ate any kind of meat? (Enter "0" if you have never eaten meat in your lifetime)" |
| Beef intake | ukb-b-2862 | 461,053 | Meat and fish | "How often do you eat beef? (Do not count processed meats)" |
| lamb/mutton intake | ukb-b-14179 | 460,006 | Meat and fish | "How often do you eat lamb/mutton? (Do not count processed meats)" |
| pork intake | ukb-b-5640 | 460,162 | Meat and fish | "How often do you eat pork? (Do not count processed meats such as bacon or ham)" |
| Poultry intake | ukb-b-8006 | 461,900 | Meat and fish | "How often do you eat chicken, turkey or other poultry? (Do not count processed meats)" |
| Processed meat intake | ukb-b-6324 | 461,981 | Meat and fish | "How often do you eat processed meats (such as bacon, ham, sausages, meat pies, kebabs, burgers, chicken nuggets)?" |
| Oily fish intake | ukb-b-2209 | 460,443 | Meat and fish | "How often do you eat oily fish? (e.g. sardines, salmon, mackerel, herring)" |
| Non-oily fish intake | ukb-b-17627 | 460,880 | Meat and fish | "How often do you eat other types of fish? (e.g. cod, tinned tuna, haddock)" |
| Cheese intake | ukb-b-1489 | 451,486 | Diary products | "How often do you eat cheese? (Include cheese in pizzas, quiches, cheese sauce etc)" |
| Milk type used: Full cream | ukb-d-1418_1 | 360,806 | Diary products | "What type of milk do you mainly use?"If you use more than one type of milk, please select the one that you drink the most. |
| Milk type used: Semi-skimmed | ukb-d-1418_2 | 360,806 | Diary products |  |
| Milk type used: Skimmed | ukb-d-1418_3 | 360,806 | Diary products |  |
| Milk type used: Soya | ukb-d-1418_4 | 360,806 | Diary products |  |
| Milk type used: Other type of milk | ukb-d-1418_5 | 360,806 | Diary products |  |
| Milk type used: Never/rarely have milk | ukb-d-1418_6 | 360,806 | Diary products |  |
| Coffee intake | ukb-b-5237 | 428,860 | Drinks | "How many cups of coffee do you drink each DAY? (Include decaffeinated coffee)" |
| Coffee type: Decaffeinated coffee (any type) | ukb-d-1508_1 | 283,449 | Drinks | "What type of coffee do you usually drink?"If you drink more than one type of coffee, please select the one that you drink the most. |
| Coffee type: Instant coffee | ukb-d-1508_2 | 283,449 | Drinks |  |
| Coffee type: Ground coffee (include espresso, filter etc) | ukb-d-1508_3 | 283,449 | Drinks |  |
| Coffee type: Other type of coffee | ukb-d-1508_4 | 283,449 | Drinks |  |
| Tea intake | ukb-b-6066 | 447,485 | Drinks | "How many cups of tea do you drink each DAY? (Include black and green tea)" |
| Water intake | ukb-b-14898 | 427,588 | Drinks | "How many glasses of water do you drink each DAY? " |
| Hot drink temperature | ukb-b-14203 | 457,873 | Drinks | "How do you like your hot drinks? (Such as coffee or tea)" |
| Alcohol usually taken with meals | ukb-b-16878 | 235,645 | Drinks | "When you drink alcohol is it usually with meals?" |
| Average weekly red wine intake | ukb-b-5239 | 327,026 | Drinks | "In an average WEEK, how many glasses of RED wine would you drink? (There are six glasses in an average bottle)" |
| Average weekly spirits intake | ukb-b-1707 | 326,565 | Drinks | "In an average WEEK, how many measures of spirits or liqueurs would you drink? (there are 25 standard measures in a normal sized bottle; spirits include drinks such as whisky, gin, rum, vodka, brandy)" |
| Average weekly fortified wine intake | ukb-b-1070 | 327,563 | Drinks | "In an average WEEK, how many glasses of fortified wine would you drink? (There are 12 glasses in an average bottle) (Fortified wines include drinks such as sherry, port, vermouth)" |
| Average weekly beer plus cider intake | ukb-b-5174 | 327,634 | Drinks | "In an average WEEK, how many pints of beer or cider would you drink? (Include bitter, lager, stout, ale, Guinness)" |
| Average weekly champagne plus white wine intake | ukb-b-5716 | 326,801 | Drinks | "In an average WEEK, how many glasses of WHITE wine or champagne would you drink? (There are six glasses in an average bottle)" |
| Salt added to food | ukb-b-8121 | 462,630 | Salt | "Do you add salt to your food? (Do not include salt used in cooking)" |

**Table S2.** Causality of genetically determined 45 dietary habits on breast cancer and endometrial cancer using IVW.

| **Exposure** | **breast cancer** | | | | | **endometrial cancer** | | | | |
| --- | --- | --- | --- | --- | --- | --- | --- | --- | --- | --- |
|  | **N snps** | **OR** | **95%CI** | **Pval** | **Q_pval** | **N snps** | **OR** | **95%CI** | **Pval** | **Q_pval** |
| Age when last ate meat | 11 | 1.092709 | 0.001-0.176 | 4.65E-01 | 9.43E-01 | 12 | 0.902148 | 0.680-1.197 | 4.76E-01 | 2.17E-02 |
| Average weekly fortified wine intake | 22 | 0.720209 | -0.934-0.278 | 2.89E-01 | 5.38E-02 | 20 | 2.883559 | 0.805-10.34 | 1.04E-01 | 4.02E-01 |
| Bread intake | 111 | 0.975762 | -0.151-0.102 | 7.04E-01 | 2.04E-14 | 117 | 0.927038 | 0.711-1.209 | 5.76E-01 | 6.55E-01 |
| lamb/mutton intake | 122 | 0.916618 | -0.279-0.105 | 3.73E-01 | 1.89E-04 | 126 | 0.799314 | 0.522-1.224 | 3.02E-01 | 6.49E-04 |
| Hot drink temperature | 202 | 0.960929 | -0.226-0.146 | 6.74E-01 | 5.38E-11 | 212 | 0.935292 | 0.682-1.283 | 6.78E-01 | 6.15E-01 |
| Cheese intake | 187 | 0.885604 | -0.224--0.019 | 2.05E-02 | 1.40E-02 | 197 | 0.763753 | 0.624-0.936 | 9.22E-03 | 9.05E-03 |
| Water intake | 153 | 0.936736 | -0.189-0.058 | 3.01E-01 | 5.56E-04 | 157 | 0.943607 | 0.712-1.250 | 6.86E-01 | 3.03E-03 |
| Cereal intake | 164 | 0.916129 | -0.221-0.046 | 1.98E-01 | 5.54E-06 | 169 | 0.826613 | 0.641-1.067 | 1.43E-01 | 1.75E-01 |
| Dried fruit intake | 149 | 0.803546 | -0.379--0.058 | 7.66E-03 | 1.38E-06 | 158 | 0.95368 | 0.717-1.269 | 7.45E-01 | 1.25E-01 |
| Alcohol usually taken with meals | 146 | 0.895573 | -0.306-0.085 | 2.69E-01 | 3.19E-02 | 150 | 1.037397 | 0.679-1.585 | 8.65E-01 | 3.23E-03 |
| Average weekly spirits intake | 41 | 0.807028 | -0.442-0.013 | 6.45E-02 | 1.12E-02 | 44 | 0.862082 | 0.521-1.426 | 5.63E-01 | 3.15E-02 |
| Non-oily fish intake | 63 | 0.994827 | -0.235-0.225 | 9.65E-01 | 2.27E-03 | 64 | 1.915353 | 1.171-3.132 | 9.61E-01 | 1.58E-01 |
| Salad / raw vegetable intake | 104 | 0.906236 | -0.310-0.113 | 3.61E-01 | 3.44E-02 | 104 | 1.04244 | 0.665-1.635 | 8.56E-01 | 6.59E-02 |
| Oily fish intake | 160 | 0.890388 | -0.235-0.003 | 5.58E-02 | 9.47E-01 | 170 | 1.065004 | 0.842-1.347 | 5.99E-01 | 6.11E-02 |
| Beef intake | 92 | 1.178078 | -0.038-0.366 | 1.12E-01 | 1.07E-02 | 102 | 0.922848 | 0.627-1.358 | 6.84E-01 | 5.06E-02 |
| Fresh fruit intake | 134 | 0.779801 | -0.446--0.052 | 1.34E-02 | 4.19E-01 | 139 | 1.261881 | 0.837-1.902 | 2.66E-01 | 3.57E-02 |
| Average weekly beer plus cider intake | 96 | 1.068442 | -0.130-0.263 | 5.09E-01 | 5.22E-03 | 103 | 0.967335 | 0.652-1.435 | 8.69E-01 | 7.22E-03 |
| Coffee intake | 108 | 0.886969 | -0.297-0.057 | 1.85E-01 | 1.17E-07 | 113 | 1.213191 | 0.902-1.632 | 2.02E-01 | 1.44E-01 |
| Average weekly red wine intake | 91 | 0.976174 | -0.165-0.117 | 7.37E-01 | 8.92E-01 | 97 | 1.263349 | 0.929-1.718 | 1.36E-01 | 1.60E-01 |
| pork intake | 77 | 1.006761 | -0.256-0.270 | 9.60E-01 | 1.08E-06 | 82 | 0.980656 | 0.596-1.613 | 9.39E-01 | 6.20E-02 |
| Average weekly champagne plus white wine intake | 49 | 0.99093 | -0.229-0.210 | 9.35E-01 | 2.87E-01 | 48 | 0.702569 | 0.448-1.103 | 1.25E-01 | 3.17E-01 |
| Tea intake | 130 | 1.033263 | -0.082-0.147 | 5.75E-01 | 9.48E-02 | 136 | 0.927437 | 0.746-1.153 | 4.97E-01 | 2.12E-01 |
| Processed meat intake | 115 | 1.012384 | -0.118-0.142 | 8.53E-01 | 5.39E-02 | 121 | 1.052775 | 0.804-1.379 | 7.09E-01 | 2.17E-01 |
| Poultry intake | 79 | 1.09617 | -0.119-0.303 | 3.93E-01 | 5.95E-03 | 81 | 0.830791 | 0.566-1.219 | 3.43E-01 | 4.44E-01 |
| Cooked vegetable intake | 89 | 0.947474 | -0.245-0.137 | 5.80E-01 | 4.85E-06 | 92 | 1.405812 | 0.943-2.095 | 9.43E-01 | 5.49E-01 |
| Salt added to food | 237 | 1.054148 | -0.040-0.145 | 2.64E-01 | 8.67E-08 | 246 | 1.246573 | 1.024-1.518 | 2.83E-01 | 7.71E-02 |
| Milk type used: Full cream | 26 | 1.038526 | -0.859-0.935 | 9.34E-01 | 3.51E-02 | 27 | 0.388788 | 0.038-3.988 | 4.26E-01 | 1.84E-01 |
| Milk type used: Semi-skimmed | 13 | 1.665983 | -0.345-1.366 | 2.42E-01 | 1.28E-03 | 15 | 2.154078 | 0.328-14.17 | 4.25E-01 | 8.22E-02 |
| Milk type used: Skimmed | 26 | 0.770097 | -0.957-0.435 | 4.62E-01 | 3.34E-10 | 29 | 0.909216 | 0.226-3.657 | 8.93E-01 | 6.42E-02 |
| Milk type used: Soya | 25 | 0.51904 | -1.930-0.618 | 3.13E-01 | 6.04E-02 | 24 | 0.863277 | 0.026-29.00 | 9.35E-01 | 1.23E-01 |
| Milk type used: Other type of milk | 13 | 0.880824 | -3.200-2.946 | 9.35E-01 | 9.43E-01 | 12 | 1.211455 | 0.001-2549 | 9.61E-01 | 4.46E-01 |
| Milk type used: Never/rarely have milk | 21 | 1.304967 | -1.700-2.232 | 7.91E-01 | 5.38E-02 | 22 | 0.759502 | 0.029-20.24 | 8.70E-01 | 6.83E-01 |
| Bread type: White | 110 | 1.154917 | -0.164-0.452 | 3.60E-01 | 2.04E-14 | 117 | 1.878588 | 1.035-3.409 | 3.81E-01 | 2.27E-02 |
| Bread type: Brown | 17 | 0.652528 | -1.294-0.44 | 3.34E-01 | 1.89E-04 | 19 | 0.712563 | 0.103-4.951 | 7.32E-01 | 7.59E-01 |
| Bread type: Wholemeal or wholegrain | 87 | 0.782333 | -0.555-0.064 | 1.20E-01 | 5.38E-11 | 91 | 1.215914 | 0.699-2.115 | 4.89E-01 | 2.35E-01 |
| Bread type: Other type of bread | 20 | 0.870777 | -1.399-1.123 | 8.30E-01 | 1.40E-02 | 22 | 0.007516 | 0.0002-0.264 | 7.07E-01 | 1.33E-02 |
| Cereal type: Bran cereal (e.g. All Bran, Branflakes) | 11 | 1.736773 | -0.493-1.597 | 3.00E-01 | 5.56E-04 | 12 | 4.551808 | 0.675-30.70 | 1.20E-01 | 6.84E-01 |
| Cereal type: Biscuit cereal (e.g. Weetabix) | 27 | 0.975566 | -0.658-0.609 | 9.39E-01 | 5.54E-06 | 27 | 0.820651 | 0.218-3.091 | 7.70E-01 | 2.94E-01 |
| Cereal type: Oat cereal (e.g. Ready Brek, porridge) | 21 | 0.926763 | -0.84-0.688 | 8.45E-01 | 1.38E-06 | 21 | 3.925515 | 0.747-20.62 | 1.06E-01 | 1.99E-02 |
| Cereal type: Muesli | 52 | 1.008991 | -0.469-0.486 | 9.71E-01 | 3.19E-02 | 58 | 0.49975 | 0.205-1.219 | 1.27E-01 | 2.37E-02 |
| Cereal type: Other (e.g. Cornflakes, Frosties) | 49 | 0.881416 | -0.682-0.43 | 6.56E-01 | 1.12E-02 | 55 | 1.078656 | 0.376-3.098 | 8.88E-01 | 6.14E-04 |
| Coffee type: Decaffeinated coffee (any type) | 18 | 2.495934 | 0.165-1.664 | 1.67E-01 | 2.27E-03 | 17 | 1.346654 | 0.265-6.850 | 7.20E-01 | 1.58E-01 |
| Coffee type: Instant coffee | 33 | 1.046862 | -0.415-0.506 | 8.45E-01 | 3.44E-02 | 32 | 0.484674 | 0.194-1.212 | 1.21E-01 | 1.38E-01 |
| Coffee type: Ground coffee (include espresso, filter etc) | 99 | 0.799785 | -0.544-0.098 | 1.72E-01 | 9.47E-01 | 104 | 0.418563 | 0.231-0.758 | 4.06E-01 | 2.13E-02 |
| Coffee type: Other type of coffee | 6 | 3.964266 | -3.338-6.093 | 5.67E-01 | 1.07E-02 | 5 | 0.249973 | 5E-05-1334 | 7.52E-01 | 6.00E-01 |

**Table S3.** Causality of genetically determined 45 dietary habits on EH and NEH of endometrial cancer using IVW.

| **Exposure** | **endometrial cancer(endometrioid)** | | | | | **endometrial cancer(non-endometrioid)** | | | | |
| --- | --- | --- | --- | --- | --- | --- | --- | --- | --- | --- |
|  | **N snps** | **OR** | **95%CI** | **Pval** | **Q_pval** | **N snps** | **OR** | **95%CI** | **Pval** | **Q_pval** |
| Age when last ate meat | 12 | 0.847768 | 0.624-1.151 | 2.90E-01 | 6.82E-02 | 12 | 0.76067 | 0.415-1.396 | 3.77E-01 | 3.82E-01 |
| Average weekly fortified wine intake | 20 | 5.844611 | 1.333-25.632 | 1.92E-02 | 5.59E-01 | 18 | 0.585934 | 0.014-25.12 | 7.80E-01 | 9.93E-01 |
| Bread intake | 119 | 0.874535 | 0.63-1.214 | 4.24E-01 | 1.87E-01 | 92 | 0.955743 | 0.408-2.239 | 9.17E-01 | 1.00E+00 |
| lamb/mutton intake | 129 | 0.791464 | 0.475-1.318 | 3.69E-01 | 1.04E-04 | 112 | 0.783788 | 0.267-2.297 | 6.57E-01 | 1.00E+00 |
| Hot drink temperature | 215 | 1.058709 | 0.724-1.547 | 7.68E-01 | 2.89E-01 | 187 | 1.178327 | 0.451-3.077 | 7.38E-01 | 1.00E+00 |
| Cheese intake | 203 | 0.705487 | 0.545-0.914 | 8.21E-03 | 5.00E-01 | 170 | 0.90199 | 0.515-1.579 | 7.18E-01 | 1.00E+00 |
| Water intake | 161 | 0.992461 | 0.707-1.393 | 9.65E-01 | 2.98E-01 | 128 | 0.750379 | 0.347-1.624 | 4.66E-01 | 1.00E+00 |
| Cereal intake | 175 | 0.762234 | 0.552-1.052 | 9.87E-02 | 4.76E-01 | 143 | 0.642537 | 0.300-1.376 | 2.55E-01 | 1.00E+00 |
| Dried fruit intake | 158 | 0.966484 | 0.686-1.362 | 8.46E-01 | 7.55E-02 | 135 | 0.707018 | 0.307-1.626 | 4.15E-01 | 1.00E+00 |
| Alcohol usually taken with meals | 151 | 1.087985 | 0.650-1.820 | 7.48E-01 | 4.15E-01 | 141 | 1.337934 | 0.453-3.955 | 5.99E-01 | 7.79E-01 |
| Average weekly spirits intake | 44 | 0.830975 | 0.457-1.511 | 5.44E-01 | 2.68E-01 | 40 | 0.265264 | 0.074-0.945 | 4.07E-01 | 9.96E-01 |
| Non-oily fish intake | 67 | 1.838947 | 0.924-3.659 | 8.27E-02 | 2.43E-01 | 62 | 1.348501 | 0.356-5.114 | 6.60E-01 | 9.86E-01 |
| Salad / raw vegetable intake | 108 | 0.973254 | 0.551-1.718 | 9.26E-01 | 2.24E-01 | 87 | 1.042781 | 0.289-3.763 | 9.49E-01 | 9.99E-01 |
| Oily fish intake | 172 | 1.067626 | 0.809-1.409 | 6.43E-01 | 5.38E-02 | 154 | 1.064021 | 0.553-2.046 | 8.52E-01 | 1.00E+00 |
| Beef intake | 102 | 0.932064 | 0.578-1.503 | 7.73E-01 | 9.02E-01 | 79 | 1.304339 | 0.42-4.051 | 6.46E-01 | 1.00E+00 |
| Fresh fruit intake | 142 | 1.44551 | 0.877-2.383 | 1.49E-01 | 5.40E-01 | 119 | 0.830026 | 0.268-2.575 | 7.47E-01 | 9.99E-01 |
| Average weekly beer plus cider intake | 103 | 1.022746 | 0.641-1.632 | 9.25E-01 | 6.71E-01 | 93 | 0.799567 | 0.284-2.251 | 6.72E-01 | 9.89E-01 |
| Coffee intake | 116 | 1.059157 | 0.737-1.521 | 7.56E-01 | 4.15E-01 | 100 | 0.796905 | 0.345-1.84 | 5.95E-01 | 1.00E+00 |
| Average weekly red wine intake | 99 | 1.030855 | 0.693-1.534 | 8.81E-01 | 5.94E-03 | 84 | 1.351092 | 0.549-3.323 | 5.12E-01 | 9.86E-01 |
| pork intake | 83 | 0.822926 | 0.447-1.514 | 5.31E-01 | 1.58E-01 | 70 | 1.149621 | 0.287-4.597 | 8.44E-01 | 9.97E-01 |
| Average weekly champagne plus white wine intake | 48 | 0.623062 | 0.366-1.06 | 8.07E-02 | 3.33E-01 | 42 | 0.568329 | 0.149-2.173 | 4.09E-01 | 9.99E-01 |
| Tea intake | 139 | 0.879637 | 0.667-1.16 | 3.64E-01 | 1.13E-01 | 118 | 0.779088 | 0.413-1.47 | 4.41E-01 | 9.99E-01 |
| Processed meat intake | 127 | 1.174656 | 0.841-1.641 | 3.46E-01 | 2.19E-01 | 105 | 0.832298 | 0.375-1.849 | 6.52E-01 | 1.00E+00 |
| Poultry intake | 83 | 0.905966 | 0.554-1.482 | 6.94E-01 | 7.61E-02 | 64 | 0.596415 | 0.174-2.043 | 4.11E-01 | 1.00E+00 |
| Cooked vegetable intake | 96 | 1.687959 | 0.98-2.907 | 5.91E-02 | 8.08E-01 | 73 | 1.192382 | 0.332-4.287 | 7.88E-01 | 1.00E+00 |
| Salt added to food | 250 | 1.247093 | 0.982-1.583 | 6.99E-02 | 1.44E-01 | 213 | 1.17667 | 0.668-2.071 | 5.73E-01 | 1.00E+00 |
| Milk type used: Full cream | 28 | 0.681501 | 0.028-16.593 | 8.14E-01 | 1.00E-01 | 26 | 2.47317 | 0.005-1235 | 7.75E-01 | 9.96E-01 |
| Milk type used: Semi-skimmed | 15 | 1.368644 | 0.186-10.054 | 7.58E-01 | 2.30E-01 | 14 | 4.673611 | 0.047-462.8 | 5.11E-01 | 7.05E-01 |
| Milk type used: Skimmed | 29 | 1.063355 | 0.194-5.842 | 9.44E-01 | 3.36E-01 | 23 | 1.608086 | 0.038-68.0 | 8.04E-01 | 9.74E-01 |
| Milk type used: Soya | 24 | 0.306238 | 0.004-23.457 | 5.93E-01 | 6.93E-02 | 18 | 26234.02 | 1-6.4E+08 | 4.85E-01 | 9.74E-01 |
| Milk type used: Other type of milk | 12 | 0.987965 | 0-179675.654 | 9.98E-01 | 4.85E-01 | 12 | 576.5668 | 1E-07-3E+12 | 5.75E-01 | 9.42E-01 |
| Milk type used: Never/rarely have milk | 22 | 0.302904 | 0.006-14.702 | 5.47E-01 | 6.32E-01 | 17 | 0.215221 | 4E-06-9827 | 7.79E-01 | 9.56E-01 |
| Bread type: White | 120 | 1.827819 | 0.868-3.848 | 1.12E-01 | 7.00E-01 | 107 | 1.509598 | 0.308-7.389 | 6.11E-01 | 1.00E+00 |
| Bread type: Brown | 19 | 0.842031 | 0.085-8.361 | 8.83E-01 | 9.23E-01 | 17 | 1.600601 | 0.004-598.3 | 8.76E-01 | 8.58E-01 |
| Bread type: Wholemeal or wholegrain | 93 | 0.996014 | 0.5-1.985 | 9.91E-01 | 4.74E-01 | 83 | 0.807786 | 0.165-3.961 | 7.92E-01 | 9.98E-01 |
| Bread type: Other type of bread | 22 | 0.005018 | 6E-05-0.393 | 1.73E-02 | 7.53E-02 | 21 | 1.219087 | 0.001-2616 | 9.60E-01 | 8.76E-01 |
| Cereal type: Bran cereal (e.g. All Bran, Branflakes) | 12 | 4.724344 | 0.495-45.053 | 1.77E-01 | 4.79E-01 | 9 | 0.043394 | 7.3E-05-26 | 3.36E-01 | 7.92E-01 |
| Cereal type: Biscuit cereal (e.g. Weetabix) | 27 | 0.914846 | 0.209-4.008 | 9.06E-01 | 6.43E-01 | 23 | 0.555075 | 0.011-28.51 | 7.70E-01 | 9.40E-01 |
| Cereal type: Oat cereal (e.g. Ready Brek, porridge) | 21 | 4.435458 | 0.611-32.188 | 1.41E-01 | 1.75E-01 | 18 | 2.366442 | 0.047-118.4 | 6.66E-01 | 8.43E-01 |
| Cereal type: Muesli | 59 | 0.332108 | 0.117-0.943 | 3.84E-02 | 2.34E-01 | 50 | 0.328399 | 0.032-3.37 | 3.49E-01 | 9.96E-01 |
| Cereal type: Other (e.g. Cornflakes, Frosties) | 55 | 1.575231 | 0.458-5.416 | 4.71E-01 | 9.48E-02 | 45 | 0.723576 | 0.058-9.058 | 8.02E-01 | 9.59E-01 |
| Coffee type: Decaffeinated coffee (any type) | 17 | 0.649295 | 0.1-4.198 | 6.50E-01 | 2.14E-01 | 16 | 6.629885 | 0.099-445.3 | 3.78E-01 | 7.46E-01 |
| Coffee type: Instant coffee | 33 | 0.691996 | 0.226-2.115 | 5.18E-01 | 6.28E-02 | 28 | 0.490259 | 0.04-5.967 | 5.76E-01 | 8.39E-01 |
| Coffee type: Ground coffee (include espresso, filter etc) | 104 | 0.369316 | 0.186-0.733 | 4.42E-03 | 4.55E-01 | 94 | 0.225525 | 0.047-1.085 | 6.31E-02 | 9.94E-01 |
| Coffee type: Other type of coffee | 6 | 570.1296 | 0.0001-1.9E+09 | 4.07E-01 | 1.68E-01 | 4 | 3.645748 | 2E-12-7E+12 | 9.28E-01 | 5.27E-01 |

**Table S4.** Causality of genetically determined 45 dietary habits on BC(ER+) and BC(ER-) of breast cancer using IVW.

| **Exposure** | **estrogen receptor positive breast cancer(ER+)** | | | | | **estrogen receptor negative breast cancer(ER-)** | | | | |
| --- | --- | --- | --- | --- | --- | --- | --- | --- | --- | --- |
|  | **N snps** | **OR** | **95%CI** | **Pval** | **Q_pval** | **N snps** | **OR** | **95%CI** | **Pval** | **Q_pval** |
| Age when last ate meat | 11 | 1.034305 | 0.932-1.148 | 5.25E-01 | 8.25E-01 | 11 | 1.322145 | 1.127-1.552 | 6.30E-01 | 6.86E-01 |
| Average weekly fortified wine intake | 22 | 0.747498 | 0.415-1.348 | 3.33E-01 | 6.80E-01 | 22 | 0.663524 | 0.211-2.083 | 4.82E-01 | 4.36E-02 |
| Bread intake | 110 | 0.972679 | 0.847-1.117 | 6.96E-01 | 4.13E-01 | 112 | 0.959521 | 0.761-1.21 | 7.27E-01 | 3.78E-02 |
| lamb/mutton intake | 122 | 0.930876 | 0.753-1.151 | 5.09E-01 | 8.20E-04 | 124 | 0.958034 | 0.719-1.276 | 7.70E-01 | 1.15E-01 |
| Hot drink temperature | 204 | 0.959403 | 0.778-1.183 | 6.98E-01 | 1.84E-10 | 200 | 1.224735 | 0.919-1.632 | 1.66E-01 | 2.09E-04 |
| Cheese intake | 184 | 0.881447 | 0.787-0.987 | 2.88E-02 | 1.31E-05 | 186 | 0.784146 | 0.676-0.91 | 1.34E-03 | 1.17E-01 |
| Water intake | 154 | 0.952411 | 0.828-1.096 | 4.95E-01 | 5.26E-03 | 153 | 0.908605 | 0.739-1.117 | 3.62E-01 | 2.91E-02 |
| Cereal intake | 163 | 0.993924 | 0.861-1.147 | 9.34E-01 | 1.33E-03 | 165 | 0.767532 | 0.634-0.929 | 6.46E-03 | 3.05E-01 |
| Dried fruit intake | 147 | 0.842615 | 0.706-1.005 | 5.69E-02 | 2.70E-06 | 150 | 0.726505 | 0.58-0.91 | 5.38E-03 | 6.69E-02 |
| Alcohol usually taken with meals | 147 | 0.950222 | 0.756-1.194 | 6.61E-01 | 1.64E-05 | 147 | 0.767969 | 0.55-1.073 | 1.22E-01 | 2.98E-04 |
| Average weekly spirits intake | 41 | 0.838168 | 0.656-1.071 | 1.58E-01 | 1.19E-01 | 42 | 0.701481 | 0.444-1.109 | 1.29E-01 | 3.38E-04 |
| Non-oily fish intake | 63 | 0.931875 | 0.711-1.221 | 6.09E-01 | 1.38E-02 | 63 | 1.169254 | 0.814-1.679 | 3.97E-01 | 2.74E-01 |
| Salad / raw vegetable intake | 104 | 0.897282 | 0.699-1.151 | 3.94E-01 | 1.05E-03 | 105 | 0.935437 | 0.667-1.313 | 6.99E-01 | 8.10E-02 |
| Oily fish intake | 161 | 0.940562 | 0.818-1.081 | 3.89E-01 | 2.14E-05 | 163 | 0.963411 | 0.789-1.176 | 7.14E-01 | 6.43E-04 |
| Beef intake | 93 | 1.194976 | 0.965-1.481 | 1.03E-01 | 4.62E-03 | 92 | 0.97037 | 0.691-1.362 | 8.62E-01 | 1.13E-03 |
| Fresh fruit intake | 133 | 0.789573 | 0.641-0.972 | 2.61E-02 | 4.37E-02 | 131 | 0.825854 | 0.605-1.127 | 2.28E-01 | 1.23E-01 |
| Average weekly beer plus cider intake | 96 | 1.069198 | 0.85-1.345 | 5.68E-01 | 8.91E-06 | 96 | 1.037726 | 0.754-1.429 | 8.21E-01 | 2.20E-03 |
| Coffee intake | 108 | 0.869749 | 0.715-1.057 | 1.61E-01 | 6.77E-08 | 107 | 0.790053 | 0.613-1.018 | 6.85E-02 | 8.57E-03 |
| Average weekly red wine intake | 93 | 0.970947 | 0.808-1.166 | 7.53E-01 | 2.55E-04 | 94 | 0.80469 | 0.634-1.021 | 7.38E-02 | 1.25E-01 |
| pork intake | 76 | 0.93183 | 0.716-1.213 | 6.00E-01 | 2.99E-02 | 80 | 1.035144 | 0.72-1.488 | 8.52E-01 | 1.99E-01 |
| Average weekly champagne plus white wine intake | 49 | 1.091523 | 0.851-1.4 | 4.91E-01 | 4.22E-02 | 49 | 0.727303 | 0.496-1.065 | 1.02E-01 | 3.96E-02 |
| Tea intake | 131 | 1.01339 | 0.897-1.145 | 8.31E-01 | 5.06E-03 | 129 | 1.017525 | 0.839-1.234 | 8.60E-01 | 1.26E-03 |
| Processed meat intake | 117 | 0.968123 | 0.829-1.131 | 6.82E-01 | 1.26E-03 | 119 | 1.278618 | 1.04-1.572 | 1.97E-01 | 1.91E-01 |
| Poultry intake | 79 | 1.177267 | 0.945-1.467 | 1.46E-01 | 2.66E-02 | 77 | 1.214754 | 0.898-1.643 | 2.07E-01 | 3.73E-01 |
| Cooked vegetable intake | 89 | 1.02947 | 0.832-1.274 | 7.89E-01 | 1.96E-01 | 91 | 0.957127 | 0.702-1.305 | 7.82E-01 | 3.65E-01 |
| Salt added to food | 237 | 1.029422 | 0.928-1.142 | 5.85E-01 | 4.10E-03 | 234 | 1.092521 | 0.928-1.287 | 2.89E-01 | 6.11E-04 |
| Milk type used: Full cream | 26 | 0.917628 | 0.316-2.667 | 8.75E-01 | 8.65E-01 | 26 | 2.331339 | 0.454-11.98 | 3.11E-01 | 7.97E-01 |
| Milk type used: Semi-skimmed | 13 | 1.63267 | 0.672-3.967 | 2.79E-01 | 2.81E-01 | 13 | 1.304314 | 0.375-4.534 | 6.76E-01 | 5.63E-01 |
| Milk type used: Skimmed | 26 | 0.931539 | 0.461-1.882 | 8.43E-01 | 1.70E-01 | 25 | 0.621715 | 0.178-2.171 | 4.56E-01 | 2.34E-02 |
| Milk type used: Soya | 25 | 0.592617 | 0.118-2.985 | 5.26E-01 | 2.42E-01 | 25 | 0.17376 | 0.018-1.689 | 1.32E-01 | 4.66E-01 |
| Milk type used: Other type of milk | 13 | 0.536187 | 0.008-36.078 | 7.72E-01 | 1.71E-01 | 13 | 4.160983 | 0.016-1075.647 | 6.15E-01 | 7.59E-01 |
| Milk type used: Never/rarely have milk | 21 | 2.237867 | 0.281-17.796 | 4.46E-01 | 5.27E-02 | 21 | 3.98321 | 0.245-64.851 | 3.32E-01 | 2.32E-01 |
| Bread type: White | 111 | 1.202395 | 0.858-1.685 | 2.84E-01 | 8.12E-05 | 111 | 1.72162 | 1.087-2.725 | 2.05E-01 | 3.14E-02 |
| Bread type: Brown | 18 | 0.388406 | 0.142-1.061 | 6.51E-02 | 5.51E-01 | 19 | 0.095556 | 0.013-0.695 | 2.04E-01 | 2.36E-02 |
| Bread type: Wholemeal or wholegrain | 89 | 0.798448 | 0.561-1.137 | 2.12E-01 | 9.04E-06 | 87 | 0.536646 | 0.338-0.853 | 8.49E-01 | 3.64E-02 |
| Bread type: Other type of bread | 20 | 1.135957 | 0.279-4.618 | 8.59E-01 | 8.96E-01 | 20 | 1.106526 | 0.08-15.356 | 9.40E-01 | 8.10E-02 |
| Cereal type: Bran cereal (e.g. All Bran, Branflakes) | 11 | 2.927092 | 1.052-8.142 | 3.96E-02 | 3.86E-01 | 11 | 3.124696 | 0.702-13.903 | 1.35E-01 | 5.48E-01 |
| Cereal type: Biscuit cereal (e.g. Weetabix) | 27 | 1.039735 | 0.447-2.416 | 9.28E-01 | 5.09E-03 | 27 | 1.021371 | 0.392-2.664 | 9.66E-01 | 4.37E-01 |
| Cereal type: Oat cereal (e.g. Ready Brek, porridge) | 21 | 1.332267 | 0.568-3.123 | 5.09E-01 | 2.36E-02 | 21 | 0.571043 | 0.181-1.804 | 3.40E-01 | 1.53E-01 |
| Cereal type: Muesli | 52 | 1.181873 | 0.716-1.95 | 5.13E-01 | 2.44E-03 | 53 | 0.800275 | 0.385-1.665 | 5.51E-01 | 6.56E-03 |
| Cereal type: Other (e.g. Cornflakes, Frosties) | 49 | 0.6699 | 0.372-1.205 | 1.81E-01 | 1.26E-04 | 51 | 1.157904 | 0.493-2.718 | 7.36E-01 | 1.88E-04 |
| Coffee type: Decaffeinated coffee (any type) | 18 | 2.296148 | 0.878-6.007 | 9.02E-02 | 1.08E-02 | 18 | 1.721632 | 0.497-5.969 | 3.92E-01 | 1.21E-01 |
| Coffee type: Instant coffee | 33 | 0.854767 | 0.496-1.472 | 5.71E-01 | 1.91E-03 | 33 | 1.958499 | 0.971-3.95 | 6.04E-02 | 9.35E-02 |
| Coffee type: Ground coffee (include espresso, filter etc) | 100 | 0.857342 | 0.594-1.238 | 4.11E-01 | 1.76E-08 | 99 | 0.53901 | 0.34-0.855 | 8.65E-03 | 1.50E-02 |
| Coffee type: Other type of coffee | 6 | 3.782502 | 0.01-1469.789 | 6.62E-01 | 3.56E-02 | 6 | 28.01616 | 0.033-23628.59 | 3.32E-01 | 2.55E-01 |

**Table S5.** Causality of genetically determined 45 dietary habits on lung cancer and ovarian cancer using IVW.

| **Exposure** | **lung cancer** | | | | | **ovarian cancer** | | | | |
| --- | --- | --- | --- | --- | --- | --- | --- | --- | --- | --- |
|  | **N snps** | **OR** | **95%CI** | **Pval** | **Q_pval** | **N snps** | **OR** | **95%CI** | **Pval** | **Q_pval** |
| Age when last ate meat | 9 | 0.911194 | 0.696-1.193 | 4.98E-01 | 5.82E-01 | 11 | 0.949916 | 0.791-1.14 | 5.81E-01 | 4.08E-01 |
| Average weekly fortified wine intake | 14 | 0.447926 | 0.083-2.406 | 3.49E-01 | 9.59E-01 | 22 | 0.591843 | 0.209-1.678 | 3.24E-01 | 5.49E-01 |
| Bread intake | 84 | 0.940489 | 0.652-1.357 | 7.43E-01 | 9.99E-01 | 105 | 1.108552 | 0.866-1.42 | 4.14E-01 | 9.84E-01 |
| lamb/mutton intake | 95 | 1.008475 | 0.631-1.611 | 9.72E-01 | 1.00E+00 | 115 | 0.881878 | 0.641-1.213 | 4.40E-01 | 9.82E-01 |
| Hot drink temperature | 151 | 0.790465 | 0.517-1.208 | 2.78E-01 | 1.00E+00 | 199 | 1.069542 | 0.807-1.418 | 6.40E-01 | 9.71E-01 |
| Cheese intake | 143 | 0.94096 | 0.737-1.201 | 6.25E-01 | 1.00E+00 | 182 | 0.864834 | 0.735-1.018 | 8.10E-02 | 9.90E-01 |
| Water intake | 110 | 1.069907 | 0.768-1.49 | 6.89E-01 | 1.00E+00 | 147 | 1.038625 | 0.835-1.291 | 7.33E-01 | 8.66E-01 |
| Cereal intake | 119 | 0.824659 | 0.59-1.152 | 2.58E-01 | 1.00E+00 | 151 | 0.899008 | 0.72-1.122 | 3.47E-01 | 9.92E-01 |
| Dried fruit intake | 113 | 0.739882 | 0.51-1.074 | 1.13E-01 | 1.00E+00 | 144 | 0.857357 | 0.67-1.096 | 2.20E-01 | 9.99E-01 |
| Alcohol usually taken with meals | 130 | 0.450243 | 0.286-0.709 | 5.72E-04 | 9.98E-01 | 147 | 0.956773 | 0.694-1.32 | 7.88E-01 | 5.14E-01 |
| Average weekly spirits intake | 36 | 1.086038 | 0.636-1.854 | 7.62E-01 | 9.83E-01 | 42 | 0.936653 | 0.645-1.361 | 7.31E-01 | 6.83E-01 |
| Non-oily fish intake | 51 | 0.823774 | 0.464-1.462 | 5.08E-01 | 9.95E-01 | 58 | 1.472261 | 0.978-2.216 | 6.38E-02 | 9.90E-01 |
| Salad / raw vegetable intake | 74 | 1.271983 | 0.728-2.221 | 3.98E-01 | 1.00E+00 | 96 | 1.161428 | 0.801-1.684 | 4.30E-01 | 9.84E-01 |
| Oily fish intake | 121 | 0.807761 | 0.602-1.083 | 1.54E-01 | 1.00E+00 | 151 | 1.11972 | 0.914-1.371 | 2.74E-01 | 8.65E-01 |
| Beef intake | 70 | 1.110866 | 0.687-1.796 | 6.68E-01 | 1.00E+00 | 92 | 0.757916 | 0.552-1.041 | 8.67E-02 | 9.34E-01 |
| Fresh fruit intake | 98 | 0.953219 | 0.576-1.577 | 8.52E-01 | 1.00E+00 | 125 | 0.914057 | 0.649-1.287 | 6.07E-01 | 9.35E-01 |
| Average weekly beer plus cider intake | 73 | 1.74067 | 1.095-2.768 | 1.92E-02 | 1.00E+00 | 92 | 1.104464 | 0.809-1.507 | 5.31E-01 | 4.19E-01 |
| Coffee intake | 83 | 1.218219 | 0.85-1.746 | 2.83E-01 | 1.00E+00 | 104 | 1.027424 | 0.8-1.319 | 8.32E-01 | 9.31E-01 |
| Average weekly red wine intake | 75 | 0.773964 | 0.526-1.14 | 1.94E-01 | 9.99E-01 | 92 | 0.781295 | 0.6-1.017 | 6.68E-02 | 3.04E-01 |
| pork intake | 57 | 0.818076 | 0.44-1.521 | 5.26E-01 | 1.00E+00 | 72 | 0.725878 | 0.481-1.095 | 1.27E-01 | 5.98E-01 |
| Average weekly champagne plus white wine intake | 36 | 0.829078 | 0.463-1.484 | 5.28E-01 | 9.82E-01 | 45 | 0.779296 | 0.528-1.15 | 2.10E-01 | 5.66E-01 |
| Tea intake | 89 | 1.000862 | 0.751-1.333 | 9.95E-01 | 9.90E-01 | 128 | 1.007295 | 0.836-1.214 | 9.39E-01 | 7.15E-01 |
| Processed meat intake | 88 | 1.473782 | 1.045-2.079 | 2.72E-02 | 1.00E+00 | 114 | 0.806429 | 0.641-1.015 | 6.72E-02 | 9.85E-01 |
| Poultry intake | 50 | 0.969948 | 0.552-1.706 | 9.16E-01 | 1.00E+00 | 73 | 1.074943 | 0.759-1.523 | 6.84E-01 | 9.15E-01 |
| Cooked vegetable intake | 63 | 0.893712 | 0.514-1.553 | 6.90E-01 | 1.00E+00 | 89 | 1.220971 | 0.858-1.737 | 2.67E-01 | 9.04E-01 |
| Salt added to food | 183 | 1.10941 | 0.868-1.417 | 4.06E-01 | 1.00E+00 | 227 | 1.076256 | 0.911-1.271 | 3.86E-01 | 9.92E-01 |
| Milk type used: Full cream | 19 | 10.63901 | 0.634-178.457 | 1.00E-01 | 9.85E-01 | 25 | 0.673991 | 0.103-4.402 | 6.80E-01 | 7.21E-01 |
| Milk type used: Semi-skimmed | 9 | 0.693613 | 0.071-6.774 | 7.53E-01 | 9.92E-01 | 13 | 1.124528 | 0.154-8.223 | 9.08E-01 | 2.40E-02 |
| Milk type used: Skimmed | 24 | 1.318999 | 0.307-5.666 | 7.10E-01 | 9.87E-01 | 26 | 1.800141 | 0.62-5.224 | 2.79E-01 | 5.07E-01 |
| Milk type used: Soya | 16 | 0.736451 | 0.009-59.50 | 8.91E-01 | 9.99E-01 | 25 | 1.922119 | 0.138-26.834 | 6.27E-01 | 4.91E-01 |
| Milk type used: Other type of milk | 9 | 0.006123 | 0-59.964 | 2.77E-01 | 9.81E-01 | 13 | 15.22303 | 0.025-9457 | 4.07E-01 | 6.11E-01 |
| Milk type used: Never/rarely have milk | 12 | 1.336845 | 0.009-200.3 | 9.10E-01 | 7.82E-01 | 21 | 0.211032 | 0.009-4.725 | 3.27E-01 | 3.01E-01 |
| Bread type: White | 86 | 2.087144 | 1.025-4.25 | 4.25E-02 | 9.97E-01 | 108 | 1.001447 | 0.623-1.609 | 9.95E-01 | 9.19E-01 |
| Bread type: Brown | 9 | 0.416086 | 0.016-10.918 | 5.99E-01 | 8.51E-01 | 18 | 0.910258 | 0.16-5.185 | 9.16E-01 | 9.50E-01 |
| Bread type: Wholemeal or wholegrain | 59 | 0.69792 | 0.329-1.481 | 3.49E-01 | 9.97E-01 | 88 | 0.802694 | 0.504-1.278 | 3.55E-01 | 9.73E-01 |
| Bread type: Other type of bread | 12 | 0.472338 | 0.005-43.561 | 7.45E-01 | 9.99E-01 | 19 | 4.797338 | 0.419-54.928 | 2.07E-01 | 5.05E-01 |
| Cereal type: Bran cereal (e.g. All Bran, Branflakes) | 9 | 0.717085 | 0.057-9.008 | 7.97E-01 | 8.41E-01 | 11 | 1.378613 | 0.25-7.59 | 7.12E-01 | 5.57E-01 |
| Cereal type: Biscuit cereal (e.g. Weetabix) | 13 | 0.791927 | 0.101-6.19 | 8.24E-01 | 9.61E-01 | 27 | 1.144309 | 0.382-3.429 | 8.10E-01 | 7.99E-01 |
| Cereal type: Oat cereal (e.g. Ready Brek, porridge) | 10 | 0.379516 | 0.051-2.827 | 3.44E-01 | 9.09E-01 | 22 | 0.798149 | 0.273-2.331 | 6.80E-01 | 5.59E-01 |
| Cereal type: Muesli | 42 | 0.829115 | 0.299-2.298 | 7.19E-01 | 9.67E-01 | 53 | 0.898855 | 0.457-1.768 | 7.57E-01 | 6.39E-01 |
| Cereal type: Other (e.g. Cornflakes, Frosties) | 35 | 2.728515 | 0.851-8.746 | 9.12E-02 | 9.96E-01 | 49 | 0.843992 | 0.405-1.761 | 6.51E-01 | 5.25E-01 |
| Coffee type: Decaffeinated coffee (any type) | 17 | 0.906731 | 0.183-4.49 | 9.05E-01 | 6.92E-01 | 17 | 2.116888 | 0.624-7.18 | 2.29E-01 | 5.37E-01 |
| Coffee type: Instant coffee | 26 | 0.888022 | 0.308-2.562 | 8.26E-01 | 9.98E-01 | 34 | 0.957115 | 0.481-1.905 | 9.01E-01 | 7.32E-01 |
| Coffee type: Ground coffee (include espresso, filter etc) | 80 | 0.594713 | 0.298-1.186 | 1.40E-01 | 9.76E-01 | 98 | 0.820874 | 0.511-1.319 | 4.15E-01 | 3.59E-01 |
| Coffee type: Other type of coffee | 3 | 543552.4 | 0.508-5.81E+11 | 6.23E-02 | 8.43E-01 | 6 | 1.233937 | 0.002-987.37 | 9.51E-01 | 9.77E-01 |

**Table S6.** Causality of genetically determined 45 dietary habits on LUAD and LUSC of lung cancer using IVW.

| **Exposure** | **lung adenocarcinoma (LUAD)** | | | | | **lung squamous carcinoma (LUSC)** | | | | |
| --- | --- | --- | --- | --- | --- | --- | --- | --- | --- | --- |
|  | **N snps** | **OR** | **95%CI** | **Pval** | **Q_pval** | **N snps** | **OR** | **95%CI** | **Pval** | **Q_pval** |
| Age when last ate meat | 9 | 0.97718 | 0.648-1.474 | 9.12E-01 | 5.61E-01 | 9 | 0.940558 | 0.624-1.417 | 7.69E-01 | 6.86E-01 |
| Average weekly fortified wine intake | 12 | 0.737984 | 0.048-11.371 | 8.28E-01 | 8.69E-01 | 13 | 0.368091 | 0.026-5.222 | 4.60E-01 | 7.75E-01 |
| Bread intake | 79 | 0.669732 | 0.378-1.188 | 1.70E-01 | 1.00E+00 | 72 | 0.704543 | 0.388-1.281 | 2.51E-01 | 9.48E-01 |
| lamb/mutton intake | 86 | 0.946446 | 0.443-2.022 | 8.87E-01 | 1.00E+00 | 81 | 0.976368 | 0.449-2.122 | 9.52E-01 | 9.64E-01 |
| Hot drink temperature | 139 | 0.943061 | 0.481-1.848 | 8.64E-01 | 1.00E+00 | 134 | 0.99867 | 0.502-1.988 | 9.97E-01 | 1.00E+00 |
| Cheese intake | 129 | 1.069729 | 0.721-1.587 | 7.38E-01 | 1.00E+00 | 140 | 0.959144 | 0.658-1.398 | 8.28E-01 | 1.00E+00 |
| Water intake | 101 | 1.158885 | 0.686-1.958 | 5.82E-01 | 1.00E+00 | 93 | 1.09144 | 0.642-1.856 | 7.47E-01 | 1.00E+00 |
| Cereal intake | 116 | 1.153008 | 0.691-1.925 | 5.86E-01 | 1.00E+00 | 112 | 0.804434 | 0.469-1.38 | 4.29E-01 | 1.00E+00 |
| Dried fruit intake | 108 | 0.864678 | 0.489-1.528 | 6.17E-01 | 1.00E+00 | 99 | 0.621129 | 0.342-1.128 | 1.17E-01 | 1.00E+00 |
| Alcohol usually taken with meals | 121 | 0.715926 | 0.352-1.457 | 3.57E-01 | 1.00E+00 | 119 | 0.461484 | 0.223-0.957 | 3.77E-02 | 1.00E+00 |
| Average weekly spirits intake | 34 | 0.796935 | 0.341-1.861 | 6.00E-01 | 1.00E+00 | 29 | 1.426048 | 0.566-3.595 | 4.52E-01 | 1.00E+00 |
| Non-oily fish intake | 44 | 0.603495 | 0.236-1.545 | 2.92E-01 | 1.00E+00 | 34 | 1.147578 | 0.379-3.477 | 8.08E-01 | 1.00E+00 |
| Salad / raw vegetable intake | 57 | 1.106459 | 0.419-2.923 | 8.38E-01 | 1.00E+00 | 62 | 0.799394 | 0.314-2.035 | 6.39E-01 | 1.00E+00 |
| Oily fish intake | 118 | 0.791513 | 0.502-1.247 | 3.14E-01 | 1.00E+00 | 104 | 0.837888 | 0.517-1.358 | 4.73E-01 | 1.00E+00 |
| Beef intake | 67 | 1.299655 | 0.619-2.73 | 4.89E-01 | 1.00E+00 | 63 | 1.254297 | 0.576-2.732 | 5.68E-01 | 1.00E+00 |
| Fresh fruit intake | 93 | 0.878097 | 0.404-1.908 | 7.43E-01 | 1.00E+00 | 87 | 0.804303 | 0.359-1.8 | 5.96E-01 | 1.00E+00 |
| Average weekly beer plus cider intake | 64 | 1.361022 | 0.647-2.865 | 4.17E-01 | 1.00E+00 | 65 | 1.72252 | 0.835-3.555 | 1.41E-01 | 1.00E+00 |
| Coffee intake | 75 | 1.135826 | 0.644-2.005 | 6.60E-01 | 1.00E+00 | 75 | 0.917527 | 0.518-1.625 | 7.68E-01 | 1.00E+00 |
| Average weekly red wine intake | 70 | 0.828123 | 0.45-1.522 | 5.44E-01 | 1.00E+00 | 64 | 1.035386 | 0.561-1.911 | 9.11E-01 | 1.00E+00 |
| pork intake | 52 | 0.648922 | 0.249-1.694 | 3.77E-01 | 1.00E+00 | 45 | 0.863167 | 0.299-2.494 | 7.86E-01 | 1.00E+00 |
| Average weekly champagne plus white wine intake | 33 | 0.687067 | 0.274-1.722 | 4.23E-01 | 9.99E-01 | 30 | 0.87724 | 0.331-2.325 | 7.92E-01 | 9.95E-01 |
| Tea intake | 91 | 0.816143 | 0.528-1.261 | 3.60E-01 | 1.00E+00 | 79 | 0.870052 | 0.549-1.378 | 5.53E-01 | 9.97E-01 |
| Processed meat intake | 74 | 1.205976 | 0.683-2.129 | 5.19E-01 | 1.00E+00 | 80 | 1.096053 | 0.624-1.925 | 7.50E-01 | 1.00E+00 |
| Poultry intake | 51 | 1.24081 | 0.537-2.87 | 6.14E-01 | 1.00E+00 | 43 | 1.23394 | 0.496-3.069 | 6.51E-01 | 1.00E+00 |
| Cooked vegetable intake | 58 | 0.897874 | 0.38-2.124 | 8.06E-01 | 1.00E+00 | 61 | 1.228775 | 0.514-2.94 | 6.43E-01 | 1.00E+00 |
| Salt added to food | 163 | 0.979206 | 0.661-1.45 | 9.16E-01 | 1.00E+00 | 164 | 1.043778 | 0.702-1.552 | 8.32E-01 | 1.00E+00 |
| Milk type used: Full cream | 18 | 5.579043 | 0.061-511.8 | 4.56E-01 | 9.97E-01 | 17 | 3.693841 | 0.035-386.2 | 5.82E-01 | 1.00E+00 |
| Milk type used: Semi-skimmed | 11 | 1.058866 | 0.046-24.466 | 9.72E-01 | 9.55E-01 | 6 | 9.411488 | 0.117-756.2 | 3.16E-01 | 1.00E+00 |
| Milk type used: Skimmed | 21 | 0.226667 | 0.022-2.359 | 2.14E-01 | 9.76E-01 | 19 | 1.545986 | 0.119-20.00 | 7.39E-01 | 1.00E+00 |
| Milk type used: Soya | 14 | 0.19164 | 0-202.413 | 6.42E-01 | 9.90E-01 | 13 | 0.087485 | 5.7E-05-133 | 5.15E-01 | 1.00E+00 |
| Milk type used: Other type of milk | 7 | 0.128438 | 0-1561107 | 8.05E-01 | 9.79E-01 | 9 | 24.63286 | 2E-05-2E+07 | 6.50E-01 | 1.00E+00 |
| Milk type used: Never/rarely have milk | 11 | 0.875858 | 0-2449.464 | 9.74E-01 | 9.54E-01 | 12 | 0.032907 | 1.72E-05 | 3.76E-01 | 1.00E+00 |
| Bread type: White | 82 | 1.440599 | 0.47-4.413 | 5.23E-01 | 1.00E+00 | 75 | 1.831926 | 0.576-5.822 | 3.05E-01 | 1.00E+00 |
| Bread type: Brown | 9 | 0.314499 | 0.002-50.148 | 6.55E-01 | 8.90E-01 | 7 | 0.591977 | 0.002-184.78 | 8.58E-01 | 1.00E+00 |
| Bread type: Wholemeal or wholegrain | 62 | 1.059086 | 0.341-3.285 | 9.21E-01 | 1.00E+00 | 56 | 1.031459 | 0.315-3.372 | 9.59E-01 | 1.00E+00 |
| Bread type: Other type of bread | 11 | 3.737478 | 0.003-5315 | 7.22E-01 | 9.88E-01 | 10 | 0.199769 | 0.0001-343.8 | 6.72E-01 | 1.00E+00 |
| Cereal type: Bran cereal (e.g. All Bran, Branflakes) | 9 | 0.16249 | 0.003-7.731 | 3.56E-01 | 9.26E-01 | 8 | 1.249352 | 0.022-70.95 | 9.14E-01 | 1.00E+00 |
| Cereal type: Biscuit cereal (e.g. Weetabix) | 11 | 0.654303 | 0.023-18.845 | 8.05E-01 | 9.00E-01 | 14 | 1.44102 | 0.066-31.349 | 8.16E-01 | 1.00E+00 |
| Cereal type: Oat cereal (e.g. Ready Brek, porridge) | 10 | 1.066054 | 0.049-23.371 | 9.68E-01 | 9.46E-01 | 8 | 1.004924 | 0.031-32.503 | 9.98E-01 | 1.00E+00 |
| Cereal type: Muesli | 43 | 0.686553 | 0.151-3.12 | 6.26E-01 | 1.00E+00 | 38 | 0.959728 | 0.193-4.78 | 9.60E-01 | 1.00E+00 |
| Cereal type: Other (e.g. Cornflakes, Frosties) | 41 | 1.645637 | 0.325-8.335 | 5.47E-01 | 9.97E-01 | 33 | 6.892497 | 1.08-43.975 | 4.12E-01 | 1.00E+00 |
| Coffee type: Decaffeinated coffee (any type) | 15 | 0.269588 | 0.02-3.559 | 3.19E-01 | 9.98E-01 | 14 | 1.423567 | 0.101-20.11 | 7.94E-01 | 1.00E+00 |
| Coffee type: Instant coffee | 26 | 2.077055 | 0.425-10.154 | 3.67E-01 | 9.89E-01 | 23 | 1.058904 | 0.189-5.923 | 9.48E-01 | 1.00E+00 |
| Coffee type: Ground coffee (include espresso, filter etc) | 71 | 0.602398 | 0.198-1.835 | 3.72E-01 | 1.00E+00 | 72 | 0.674514 | 0.217-2.095 | 4.96E-01 | 1.00E+00 |
| Coffee type: Other type of coffee | 4 | 208244.3 | 0.005-9E+12 | 1.72E-01 | 9.14E-01 | 3 | 497.5906 | 7E-08-3E+12 | 5.90E-01 | 1.00E+00 |

**Table S7.** Causality of genetically determined 45 dietary habits on HGS and LGS of ovarian cancer using IVW.

| **Exposure** | **high grade serous ovarian cancer (HGS)** | | | | | **low grade serous ovarian cancer (LGS)** | | | | |
| --- | --- | --- | --- | --- | --- | --- | --- | --- | --- | --- |
|  | **N snps** | **OR** | **95%CI** | **Pval** | **Q_pval** | **N snps** | **OR** | **95%CI** | **Pval** | **Q_pval** |
| Age when last ate meat | 11 | 0.98702 | 0.797-1.222 | 9.04E-01 | 0.637167 | 11 | 1.340571 | 0.711-2.529 | 3.65E-01 | 6.34E-01 |
| Average weekly fortified wine intake | 21 | 0.740801 | 0.207-2.647 | 6.44E-01 | 0.975851 | 17 | 0.161206 | 0.001-19.26 | 4.55E-01 | 2.59E-01 |
| Bread intake | 104 | 1.107815 | 0.824-1.489 | 4.97E-01 | 0.978957 | 101 | 1.576578 | 0.635-3.914 | 3.26E-01 | 1.00E+00 |
| lamb/mutton intake | 113 | 0.822443 | 0.562-1.204 | 3.15E-01 | 0.981441 | 113 | 0.667783 | 0.211-2.114 | 4.92E-01 | 9.95E-01 |
| Hot drink temperature | 190 | 1.070778 | 0.762-1.506 | 6.94E-01 | 0.948495 | 187 | 0.817207 | 0.286-2.336 | 7.06E-01 | 9.73E-01 |
| Cheese intake | 174 | 0.905901 | 0.743-1.104 | 3.28E-01 | 0.998922 | 166 | 0.648026 | 0.35-1.198 | 1.67E-01 | 8.70E-01 |
| Water intake | 143 | 0.91543 | 0.705-1.189 | 5.08E-01 | 0.905075 | 136 | 0.96607 | 0.427-2.183 | 9.34E-01 | 9.16E-01 |
| Cereal intake | 148 | 0.819433 | 0.628-1.07 | 1.43E-01 | 0.994492 | 149 | 1.134564 | 0.507-2.541 | 7.59E-01 | 9.16E-01 |
| Dried fruit intake | 144 | 0.746056 | 0.557-0.999 | 4.94E-02 | 0.999553 | 132 | 1.930449 | 0.769-4.845 | 1.61E-01 | 9.87E-01 |
| Alcohol usually taken with meals | 144 | 0.888328 | 0.604-1.306 | 5.47E-01 | 0.830288 | 143 | 0.687846 | 0.212-2.231 | 5.33E-01 | 9.99E-01 |
| Average weekly spirits intake | 40 | 0.910349 | 0.578-1.434 | 6.85E-01 | 0.985763 | 39 | 1.051085 | 0.257-4.3 | 9.45E-01 | 9.06E-01 |
| Non-oily fish intake | 57 | 1.63548 | 1.001-2.672 | 4.94E-02 | 0.870623 | 56 | 1.314496 | 0.286-6.043 | 7.25E-01 | 9.99E-01 |
| Salad / raw vegetable intake | 99 | 0.902243 | 0.583-1.395 | 6.44E-01 | 0.998303 | 93 | 2.238712 | 0.578-8.666 | 2.43E-01 | 9.98E-01 |
| Oily fish intake | 146 | 1.068735 | 0.838-1.364 | 5.93E-01 | 0.949816 | 149 | 0.928753 | 0.449-1.92 | 8.42E-01 | 1.00E+00 |
| Beef intake | 90 | 0.739616 | 0.506-1.081 | 1.19E-01 | 0.998566 | 88 | 0.916779 | 0.285-2.947 | 8.84E-01 | 9.78E-01 |
| Fresh fruit intake | 124 | 0.808377 | 0.538-1.215 | 3.06E-01 | 0.947997 | 122 | 0.785215 | 0.226-2.725 | 7.03E-01 | 9.87E-01 |
| Average weekly beer plus cider intake | 87 | 1.13179 | 0.779-1.644 | 5.16E-01 | 0.628732 | 95 | 1.442268 | 0.481-4.324 | 5.13E-01 | 9.77E-01 |
| Coffee intake | 99 | 0.975144 | 0.721-1.318 | 8.70E-01 | 0.989907 | 97 | 1.158504 | 0.457-2.936 | 7.56E-01 | 9.99E-01 |
| Average weekly red wine intake | 91 | 0.752483 | 0.555-1.02 | 6.69E-02 | 0.530072 | 86 | 0.679056 | 0.263-1.755 | 4.24E-01 | 9.73E-01 |
| pork intake | 73 | 0.672728 | 0.414-1.093 | 1.10E-01 | 0.883263 | 63 | 0.888193 | 0.184-4.288 | 8.83E-01 | 9.16E-01 |
| Average weekly champagne plus white wine intake | 46 | 0.799743 | 0.505-1.266 | 3.41E-01 | 0.793716 | 41 | 1.664041 | 0.385-7.187 | 4.95E-01 | 7.97E-01 |
| Tea intake | 122 | 0.950667 | 0.758-1.192 | 6.61E-01 | 0.954918 | 122 | 0.93869 | 0.472-1.866 | 8.57E-01 | 1.00E+00 |
| Processed meat intake | 113 | 0.883253 | 0.671-1.163 | 3.76E-01 | 0.966019 | 110 | 0.91555 | 0.394-2.127 | 8.37E-01 | 1.00E+00 |
| Poultry intake | 70 | 1.194907 | 0.784-1.822 | 4.08E-01 | 0.998193 | 72 | 0.711262 | 0.201-2.52 | 5.98E-01 | 9.99E-01 |
| Cooked vegetable intake | 87 | 1.431055 | 0.938-2.184 | 9.65E-02 | 0.988916 | 85 | 0.736383 | 0.199-2.722 | 6.46E-01 | 9.97E-01 |
| Salt added to food | 223 | 1.102233 | 0.904-1.345 | 3.37E-01 | 0.987117 | 226 | 1.056765 | 0.579-1.928 | 8.57E-01 | 9.16E-01 |
| Milk type used: Full cream | 25 | 1.743872 | 0.189-16.063 | 6.24E-01 | 0.958819 | 25 | 0.897258 | 0.001-831.5 | 9.75E-01 | 7.70E-01 |
| Milk type used: Semi-skimmed | 12 | 1.420988 | 0.208-9.706 | 7.20E-01 | 0.268736 | 10 | 337.5506 | 1.015-1.1E+05 | 4.94E-01 | 1.00E+00 |
| Milk type used: Skimmed | 26 | 1.122873 | 0.32-3.935 | 8.56E-01 | 0.644474 | 24 | 3.793285 | 0.068-210 | 5.15E-01 | 9.84E-01 |
| Milk type used: Soya | 23 | 1.81147 | 0.064-50.924 | 7.27E-01 | 0.402645 | 23 | 30.15233 | 0.001-6E+05 | 5.01E-01 | 9.81E-01 |
| Milk type used: Other type of milk | 13 | 165.1799 | 0.079-346533 | 1.91E-01 | 0.729345 | 12 | 0.013743 | 2E-13-8E+08 | 7.35E-01 | 9.26E-01 |
| Milk type used: Never/rarely have milk | 21 | 0.501576 | 0.013-19.629 | 7.12E-01 | 0.318785 | 20 | 0.001 | 0-52.081 | 2.13E-01 | 9.15E-01 |
| Bread type: White | 101 | 1.093495 | 0.612-1.954 | 7.63E-01 | 0.990449 | 107 | 2.458623 | 0.441-13.715 | 3.05E-01 | 9.99E-01 |
| Bread type: Brown | 18 | 3.0582 | 0.386-24.213 | 2.90E-01 | 0.918072 | 18 | 0.443492 | 0.001-222.372 | 7.98E-01 | 8.67E-01 |
| Bread type: Wholemeal or wholegrain | 85 | 0.575071 | 0.328-1.009 | 5.38E-02 | 0.993934 | 85 | 0.523564 | 0.094-2.905 | 4.59E-01 | 9.95E-01 |
| Bread type: Other type of bread | 19 | 33.84671 | 1.869-612.9 | 1.72E-02 | 0.551688 | 16 | 4.25865 | 0.0003-5E+04 | 7.62E-01 | 8.60E-01 |
| Cereal type: Bran cereal (e.g. All Bran, Branflakes) | 11 | 0.940284 | 0.124-7.144 | 9.53E-01 | 0.629288 | 9 | 0.25351 | 0.0002-2E+02 | 6.99E-01 | 5.67E-01 |
| Cereal type: Biscuit cereal (e.g. Weetabix) | 27 | 1.111372 | 0.302-4.084 | 8.74E-01 | 0.909521 | 25 | 10.93461 | 0.176-680.312 | 2.56E-01 | 9.99E-01 |
| Cereal type: Oat cereal (e.g. Ready Brek, porridge) | 22 | 0.640679 | 0.179-2.289 | 4.93E-01 | 0.756187 | 20 | 0.053665 | 0.001-3.115 | 1.58E-01 | 9.98E-01 |
| Cereal type: Muesli | 53 | 1.055606 | 0.473-2.358 | 8.95E-01 | 0.75344 | 51 | 1.253105 | 0.103-15.249 | 8.60E-01 | 9.16E-01 |
| Cereal type: Other (e.g. Cornflakes, Frosties) | 48 | 0.58182 | 0.233-1.451 | 2.45E-01 | 0.366355 | 48 | 0.796819 | 0.057-11.13 | 8.66E-01 | 7.50E-01 |
| Coffee type: Decaffeinated coffee (any type) | 18 | 2.784759 | 0.676-11.467 | 1.56E-01 | 0.707415 | 16 | 33.4956 | 0.367-3056 | 1.27E-01 | 8.62E-01 |
| Coffee type: Instant coffee | 34 | 1.130169 | 0.499-2.561 | 7.69E-01 | 0.613483 | 33 | 2.120396 | 0.168-26.73 | 5.61E-01 | 9.84E-01 |
| Coffee type: Ground coffee (include espresso, filter etc) | 97 | 0.637948 | 0.367-1.109 | 1.11E-01 | 0.547837 | 96 | 0.405727 | 0.075-2.209 | 2.97E-01 | 9.86E-01 |
| Coffee type: Other type of coffee | 6 | 16.08823 | 0.006-43940 | 4.91E-01 | 0.952339 | 6 | 0.088903 | 1E-12-4E+09 | 8.47E-01 | 9.39E-01 |

**Table S8.** Causality of genetically determined 45 dietary habits on ED and IM of ovarian cancer using IVW.

| **Exposure** | **endometrioid ovarian cancer (ED)** | | | | | **invasive mucinous ovarian cancer (IM)** | | | | |
| --- | --- | --- | --- | --- | --- | --- | --- | --- | --- | --- |
|  | **N snps** | **OR** | **95%CI** | **Pval** | **Q_pval** | **N snps** | **OR** | **95%CI** | **Pval** | **Q_pval** |
| Age when last ate meat | 11 | 0.924937 | 0.628-1.362 | 6.93E-01 | 6.93E-01 | 11 | 0.690305 | 0.384-1.242 | 2.16E-01 | 2.81E-01 |
| Average weekly fortified wine intake | 21 | 0.724088 | 0.073-7.186 | 7.83E-01 | 7.53E-01 | 19 | 0.413321 | 0.014-11.79 | 6.05E-01 | 9.99E-01 |
| Bread intake | 98 | 0.83593 | 0.483-1.447 | 5.22E-01 | 9.98E-01 | 101 | 0.701509 | 0.331-1.487 | 3.55E-01 | 9.94E-01 |
| lamb/mutton intake | 111 | 1.206682 | 0.597-2.441 | 6.01E-01 | 9.99E-01 | 112 | 0.629104 | 0.24-1.647 | 3.45E-01 | 1.00E+00 |
| Hot drink temperature | 182 | 1.320897 | 0.699-2.497 | 3.92E-01 | 1.00E-02 | 187 | 0.775747 | 0.327-1.839 | 5.64E-01 | 1.00E+00 |
| Cheese intake | 164 | 1.058858 | 0.73-1.536 | 7.63E-01 | 1.00E-01 | 173 | 0.755743 | 0.46-1.242 | 2.69E-01 | 1.00E+00 |
| Water intake | 132 | 0.712102 | 0.435-1.165 | 1.77E-01 | 9.99E-01 | 142 | 1.655604 | 0.856-3.201 | 1.34E-01 | 1.00E+00 |
| Cereal intake | 150 | 1.061979 | 0.656-1.719 | 8.07E-01 | 1.00E-01 | 148 | 0.63917 | 0.327-1.249 | 1.90E-01 | 1.00E+00 |
| Dried fruit intake | 131 | 1.241947 | 0.715-2.157 | 4.42E-01 | 9.88E-01 | 138 | 0.701654 | 0.333-1.478 | 3.51E-01 | 1.00E+00 |
| Alcohol usually taken with meals | 144 | 1.195342 | 0.592-2.413 | 6.18E-01 | 5.85E-01 | 141 | 0.519232 | 0.195-1.384 | 1.90E-01 | 8.93E-01 |
| Average weekly spirits intake | 37 | 1.248888 | 0.522-2.986 | 6.17E-01 | 9.75E-01 | 39 | 0.874748 | 0.274-2.794 | 8.21E-01 | 9.25E-01 |
| Non-oily fish intake | 57 | 1.140062 | 0.466-2.789 | 7.74E-01 | 9.29E-01 | 60 | 0.909935 | 0.271-3.057 | 8.79E-01 | 9.77E-01 |
| Salad / raw vegetable intake | 94 | 1.023073 | 0.454-2.305 | 9.56E-01 | 1.00E-01 | 94 | 1.877762 | 0.618-5.701 | 2.66E-01 | 1.00E+00 |
| Oily fish intake | 146 | 0.989459 | 0.638-1.534 | 9.62E-01 | 9.71E-01 | 147 | 1.244697 | 0.681-2.275 | 4.77E-01 | 1.00E+00 |
| Beef intake | 85 | 0.408608 | 0.201-0.829 | 1.32E-01 | 9.96E-01 | 85 | 0.797905 | 0.301-2.115 | 6.50E-01 | 1.00E+00 |
| Fresh fruit intake | 122 | 0.803841 | 0.383-1.686 | 5.63E-01 | 8.83E-01 | 126 | 0.737236 | 0.269-2.024 | 5.54E-01 | 1.00E+00 |
| Average weekly beer plus cider intake | 90 | 0.773967 | 0.395-1.516 | 4.55E-01 | 9.90E-01 | 90 | 0.658964 | 0.26-1.672 | 3.80E-01 | 6.54E-01 |
| Coffee intake | 99 | 1.3502 | 0.779-2.34 | 2.84E-01 | 9.97E-01 | 99 | 0.717026 | 0.336-1.531 | 3.90E-01 | 9.96E-01 |
| Average weekly red wine intake | 84 | 0.989781 | 0.558-1.756 | 9.72E-01 | 9.34E-01 | 89 | 0.691201 | 0.318-1.503 | 3.52E-01 | 9.28E-01 |
| pork intake | 65 | 0.923902 | 0.362-2.357 | 8.68E-01 | 9.52E-01 | 70 | 0.605249 | 0.173-2.112 | 4.31E-01 | 9.91E-01 |
| Average weekly champagne plus white wine intake | 45 | 0.462321 | 0.198-1.08 | 7.47E-02 | 6.04E-01 | 46 | 0.356602 | 0.113-1.127 | 7.89E-02 | 9.82E-01 |
| Tea intake | 122 | 1.288742 | 0.854-1.945 | 2.27E-01 | 1.00E-01 | 119 | 0.966844 | 0.546-1.711 | 9.08E-01 | 9.92E-01 |
| Processed meat intake | 111 | 1.090949 | 0.657-1.811 | 7.36E-01 | 9.99E-01 | 105 | 0.602813 | 0.295-1.232 | 1.65E-01 | 1.00E+00 |
| Poultry intake | 66 | 1.232638 | 0.555-2.739 | 6.08E-01 | 9.87E-01 | 68 | 1.60173 | 0.546-4.703 | 3.91E-01 | 1.00E+00 |
| Cooked vegetable intake | 79 | 1.056448 | 0.47-2.374 | 8.94E-01 | 9.93E-01 | 84 | 0.836967 | 0.284-2.47 | 7.47E-01 | 9.99E-01 |
| Salt added to food | 221 | 1.135352 | 0.789-1.633 | 4.94E-01 | 1.00E-01 | 213 | 1.515364 | 0.913-2.514 | 1.08E-01 | 1.00E+00 |
| Milk type used: Full cream | 24 | 0.93338 | 0.014-61.208 | 9.74E-01 | 6.71E-01 | 26 | 0.167827 | 0.001-42.68 | 5.28E-01 | 5.13E-01 |
| Milk type used: Semi-skimmed | 11 | 2.553625 | 0.09-72.813 | 5.83E-01 | 4.60E-01 | 10 | 0.748858 | 0.005-102.91 | 9.08E-01 | 6.22E-01 |
| Milk type used: Skimmed | 26 | 3.316467 | 0.328-33.496 | 3.10E-01 | 8.68E-01 | 24 | 1.670152 | 0.06-46.48 | 7.62E-01 | 7.04E-01 |
| Milk type used: Soya | 23 | 0.089184 | 0-32.697 | 4.22E-01 | 9.91E-01 | 21 | 58.9429 | 0.012-3E+05 | 3.47E-01 | 9.82E-01 |
| Milk type used: Other type of milk | 11 | 0.484115 | 0-2.24E+06 | 9.26E-01 | 6.41E-01 | 12 | 3.975051 | 7E-09-2E+09 | 8.93E-01 | 9.00E-01 |
| Milk type used: Never/rarely have milk | 21 | 0.017619 | 0-9.839 | 2.11E-01 | 9.28E-01 | 18 | 108.5538 | 0.008-1E+06 | 3.31E-01 | 9.66E-01 |
| Bread type: White | 105 | 0.286261 | 0.101-0.811 | 1.86E-01 | 8.85E-01 | 108 | 0.826176 | 0.200-3.408 | 7.92E-01 | 9.99E-01 |
| Bread type: Brown | 15 | 0.063969 | 0.001-3.939 | 1.91E-01 | 7.02E-01 | 16 | 0.111046 | 0-27.357 | 4.34E-01 | 9.58E-01 |
| Bread type: Wholemeal or wholegrain | 83 | 2.205857 | 0.78-6.235 | 1.36E-01 | 9.47E-01 | 82 | 2.995855 | 0.714-12.58 | 1.34E-01 | 9.84E-01 |
| Bread type: Other type of bread | 19 | 0.712291 | 0.003-147.279 | 9.01E-01 | 8.15E-01 | 19 | 0.028825 | 0-48.251 | 3.49E-01 | 8.09E-01 |
| Cereal type: Bran cereal (e.g. All Bran, Branflakes) | 10 | 20.65831 | 0.42-1017.119 | 1.28E-01 | 9.48E-01 | 9 | 1.152203 | 0.002-753.67 | 9.66E-01 | 2.14E-01 |
| Cereal type: Biscuit cereal (e.g. Weetabix) | 25 | 0.548901 | 0.047-6.349 | 6.31E-01 | 8.14E-01 | 24 | 2.499797 | 0.078-80.522 | 6.05E-01 | 9.59E-01 |
| Cereal type: Oat cereal (e.g. Ready Brek, porridge) | 20 | 0.591376 | 0.052-6.674 | 6.71E-01 | 9.44E-01 | 20 | 0.708673 | 0.026-19.072 | 8.38E-01 | 5.93E-01 |
| Cereal type: Muesli | 48 | 0.813754 | 0.175-3.792 | 7.93E-01 | 8.28E-01 | 50 | 2.599869 | 0.328-20.639 | 3.66E-01 | 9.10E-01 |
| Cereal type: Other (e.g. Cornflakes, Frosties) | 50 | 3.230328 | 0.67-15.582 | 1.44E-01 | 9.74E-01 | 50 | 0.843238 | 0.097-7.368 | 8.77E-01 | 7.11E-01 |
| Coffee type: Decaffeinated coffee (any type) | 16 | 2.376024 | 0.155-36.508 | 5.35E-01 | 9.57E-01 | 16 | 6.122034 | 0.142-263.8 | 3.45E-01 | 7.03E-01 |
| Coffee type: Instant coffee | 33 | 0.848815 | 0.187-3.858 | 8.32E-01 | 8.47E-01 | 33 | 0.215603 | 0.027-1.730 | 1.49E-01 | 8.40E-01 |
| Coffee type: Ground coffee (include espresso, filter etc) | 91 | 0.917063 | 0.319-2.639 | 8.72E-01 | 4.00E-01 | 92 | 1.037397 | 0.249-4.328 | 9.60E-01 | 9.87E-01 |
| Coffee type: Other type of coffee | 5 | 3521.271 | 0.0003-3E+10 | 3.19E-01 | 7.19E-01 | 6 | 0.000203 | 0-1.25E+05 | 4.10E-01 | 8.10E-01 |

**Table S9.** Causality of genetically determined 45 dietary habits on CC of ovarian cancer and prostate cancer and using IVW.

| **Exposure** | **clear-cell ovarian cancer (CC)** | | | | | **prostate cancer** | | | | |
| --- | --- | --- | --- | --- | --- | --- | --- | --- | --- | --- |
|  | **N snps** | **OR** | **95%CI** | **Pval** | **Q_pval** | **N snps** | **OR** | **95%CI** | **Pval** | **Q_pval** |
| Age when last ate meat | 11 | 0.847417 | 0.447-1.606 | 6.12E-01 | 1.69E-01 | 12 | 1.038574 | 0.898-1.201 | 6.10E-01 | 4.06E-02 |
| Average weekly fortified wine intake | 21 | 1.139214 | 0.044-29.37 | 9.37E-01 | 8.12E-01 | 20 | 1.295734 | 0.673-2.493 | 4.38E-01 | 8.31E-01 |
| Bread intake | 103 | 1.036764 | 0.491-2.189 | 9.25E-01 | 1.00E+00 | 97 | 1.094322 | 0.936-1.279 | 2.57E-01 | 6.50E-01 |
| lamb/mutton intake | 114 | 0.685551 | 0.261-1.801 | 4.44E-01 | 9.98E-01 | 109 | 1.099482 | 0.890-1.359 | 3.80E-01 | 1.91E-01 |
| Hot drink temperature | 171 | 0.757257 | 0.306-1.874 | 5.48E-01 | 1.00E+00 | 181 | 0.922413 | 0.762-1.117 | 4.08E-01 | 1.15E-01 |
| Cheese intake | 169 | 0.845231 | 0.507-1.410 | 5.20E-01 | 1.00E+00 | 166 | 0.969373 | 0.870-1.080 | 5.72E-01 | 2.53E-01 |
| Water intake | 143 | 1.37933 | 0.707-2.690 | 3.45E-01 | 9.98E-01 | 132 | 0.920874 | 0.801-1.059 | 2.49E-01 | 4.59E-01 |
| Cereal intake | 146 | 0.669381 | 0.338-1.324 | 2.49E-01 | 1.00E+00 | 156 | 1.097060 | 0.947-1.271 | 2.18E-01 | 6.11E-02 |
| Dried fruit intake | 134 | 1.214821 | 0.565-2.611 | 6.18E-01 | 9.99E-01 | 130 | 1.145657 | 0.974-1.347 | 9.97E-02 | 3.42E-01 |
| Alcohol usually taken with meals | 145 | 1.611379 | 0.607-4.281 | 3.39E-01 | 5.32E-01 | 137 | 1.094652 | 0.886-1.353 | 4.03E-01 | 2.75E-01 |
| Average weekly spirits intake | 42 | 0.589762 | 0.189-1.842 | 3.64E-01 | 9.14E-01 | 38 | 0.956134 | 0.750-1.218 | 7.17E-01 | 4.03E-01 |
| Non-oily fish intake | 53 | 1.437192 | 0.394-5.247 | 5.83E-01 | 9.80E-01 | 59 | 1.026337 | 0.800-1.317 | 8.38E-01 | 8.98E-01 |
| Salad / raw vegetable intake | 92 | 1.193732 | 0.378-3.774 | 7.63E-01 | 9.96E-01 | 89 | 1.040967 | 0.819-1.323 | 7.43E-01 | 4.73E-01 |
| Oily fish intake | 148 | 0.890349 | 0.481-1.647 | 7.11E-01 | 9.97E-01 | 141 | 0.924786 | 0.813-1.051 | 2.32E-01 | 3.91E-01 |
| Beef intake | 78 | 0.594001 | 0.213-1.656 | 3.19E-01 | 9.90E-01 | 83 | 1.130417 | 0.921-1.388 | 2.42E-01 | 8.39E-01 |
| Fresh fruit intake | 116 | 0.784708 | 0.272-2.260 | 6.53E-01 | 9.86E-01 | 120 | 1.003206 | 0.811-1.241 | 9.77E-01 | 4.22E-01 |
| Average weekly beer plus cider intake | 87 | 0.396956 | 0.153-1.030 | 5.76E-02 | 9.95E-01 | 93 | 1.045913 | 0.856-1.277 | 6.60E-01 | 1.79E-01 |
| Coffee intake | 97 | 1.136506 | 0.525-2.461 | 7.45E-01 | 9.89E-01 | 102 | 0.993019 | 0.840-1.174 | 9.34E-01 | 9.55E-02 |
| Average weekly red wine intake | 84 | 1.24441 | 0.556-2.783 | 5.94E-01 | 9.74E-01 | 82 | 1.018441 | 0.862-1.203 | 8.29E-01 | 7.55E-01 |
| pork intake | 71 | 0.813454 | 0.232-2.856 | 7.47E-01 | 9.86E-01 | 64 | 1.05831 | 0.808-1.386 | 6.80E-01 | 7.75E-01 |
| Average weekly champagne plus white wine intake | 45 | 0.491886 | 0.151-1.599 | 2.38E-01 | 9.71E-01 | 43 | 1.260356 | 0.945-1.681 | 1.15E-01 | 4.47E-02 |
| Tea intake | 126 | 1.259484 | 0.713-2.226 | 4.27E-01 | 1.00E+00 | 117 | 0.974410 | 0.866-1.096 | 6.66E-01 | 5.29E-01 |
| Processed meat intake | 104 | 0.806627 | 0.391-1.662 | 5.60E-01 | 1.00E+00 | 111 | 0.926157 | 0.801-1.071 | 3.02E-01 | 3.99E-01 |
| Poultry intake | 72 | 0.663557 | 0.230-1.913 | 4.48E-01 | 1.00E+00 | 68 | 0.905039 | 0.725-1.130 | 3.78E-01 | 7.45E-01 |
| Cooked vegetable intake | 75 | 1.192385 | 0.375-3.795 | 7.66E-01 | 9.93E-01 | 80 | 0.919036 | 0.732-1.153 | 4.66E-01 | 7.77E-01 |
| Salt added to food | 215 | 0.956784 | 0.574-1.594 | 8.65E-01 | 1.00E+00 | 203 | 1.001557 | 0.898-1.117 | 9.78E-01 | 5.75E-01 |
| Milk type used: Full cream | 24 | 0.031086 | 9.8E-05-9.822 | 2.37E-01 | 9.25E-01 | 23 | 3.906263 | 1.181-12.92 | 2.55E-01 | 6.41E-01 |
| Milk type used: Semi-skimmed | 12 | 2.436537 | 0.027-221.77 | 6.99E-01 | 9.38E-01 | 12 | 0.917059 | 0.330-2.545 | 8.68E-01 | 2.58E-01 |
| Milk type used: Skimmed | 25 | 32.82606 | 1.248-863.519 | 3.64E-01 | 4.55E-01 | 23 | 0.424855 | 0.216-0.837 | 1.33E-01 | 6.16E-01 |
| Milk type used: Soya | 25 | 1.714909 | 0.001-5025 | 8.95E-01 | 9.18E-01 | 21 | 3.034799 | 0.438-21.02 | 2.61E-01 | 2.09E-01 |
| Milk type used: Other type of milk | 12 | 38.06127 | 7.4E-08-1.95E+10 | 7.22E-01 | 6.73E-01 | 11 | 0.580014 | 0.011-32.01 | 7.90E-01 | 5.03E-01 |
| Milk type used: Never/rarely have milk | 18 | 0.050251 | 4.0E-06-622.5 | 5.34E-01 | 9.52E-01 | 19 | 1.727517 | 0.27-11.062 | 5.64E-01 | 5.71E-01 |
| Bread type: White | 102 | 0.360723 | 0.083-1.576 | 1.75E-01 | 9.98E-01 | 113 | 0.847643 | 0.608-1.181 | 3.29E-01 | 8.78E-03 |
| Bread type: Brown | 16 | 0.157958 | 0.001-40.115 | 5.14E-01 | 9.16E-01 | 15 | 0.54507 | 0.168-1.770 | 3.13E-01 | 4.61E-01 |
| Bread type: Wholemeal or wholegrain | 81 | 1.866814 | 0.431-8.088 | 4.04E-01 | 9.54E-01 | 64 | 0.928534 | 0.651-1.324 | 6.82E-01 | 2.42E-01 |
| Bread type: Other type of bread | 18 | 20.23047 | 0.011-38552 | 4.35E-01 | 6.93E-01 | 16 | 0.508311 | 0.061-4.208 | 5.30E-01 | 1.64E-01 |
| Cereal type: Bran cereal (e.g. All Bran, Branflakes) | 9 | 2.995166 | 0.009-995.5 | 7.11E-01 | 6.67E-01 | 12 | 2.646535 | 0.934-7.502 | 6.71E-02 | 3.84E-01 |
| Cereal type: Biscuit cereal (e.g. Weetabix) | 25 | 0.897625 | 0.03-26.682 | 9.50E-01 | 9.04E-01 | 27 | 1.043869 | 0.536-2.032 | 8.99E-01 | 8.33E-01 |
| Cereal type: Oat cereal (e.g. Ready Brek, porridge) | 21 | 1.772857 | 0.064-49.136 | 7.35E-01 | 6.47E-01 | 17 | 1.277409 | 0.508-3.214 | 6.03E-01 | 9.23E-02 |
| Cereal type: Muesli | 49 | 1.913433 | 0.226-16.19 | 5.51E-01 | 9.96E-01 | 48 | 1.219822 | 0.789-1.885 | 3.71E-01 | 9.75E-01 |
| Cereal type: Other (e.g. Cornflakes, Frosties) | 47 | 0.11546 | 0.012-1.096 | 6.01E-02 | 8.93E-01 | 45 | 0.70037 | 0.402-1.220 | 2.08E-01 | 4.49E-02 |
| Coffee type: Decaffeinated coffee (any type) | 17 | 1.439438 | 0.035-58.48 | 8.47E-01 | 4.89E-01 | 16 | 0.810707 | 0.346-1.901 | 6.29E-01 | 2.64E-01 |
| Coffee type: Instant coffee | 28 | 1.245364 | 0.092-16.88 | 8.69E-01 | 1.17E-01 | 31 | 1.029041 | 0.620-1.709 | 9.12E-01 | 1.12E-01 |
| Coffee type: Ground coffee (include espresso, filter etc) | 94 | 1.729252 | 0.412-7.256 | 4.54E-01 | 8.85E-01 | 88 | 1.17115 | 0.826-1.660 | 3.75E-01 | 1.72E-02 |
| Coffee type: Other type of coffee | 6 | 162991.6 | 0.0002-9.63E+13 | 2.44E-01 | 6.19E-01 | 5 | 0.008545 | 7.6E-05-0.956 | 4.79E-01 | 3.54E-01 |

**Table S10.** Causality of genetically determined 45 dietary habits on breast cancer in sensitivity analysis.

| **Exposure** | **Weighted median** | | | **MR Egger** | | | | | **MR Presso** | | |
| --- | --- | --- | --- | --- | --- | --- | --- | --- | --- | --- | --- |
|  | **OR** | **95%CI** | **Pval** | **OR** | **95%CI** | **Pval** | **Intercept** | **Intercept**  **Pval** | **OR** | **Pval** | **N outliers** |
| Age when last ate meat | 1.215184 | 0.908-1.626 | 2.22E-01 | 1.215184 | 0.908-1.626 | 2.22E-01 | -8.39E-03 | 0.473 | 1.092709 | 1.10E-02 | 0 |
| Average weekly fortified wine intake | 0.39033 | 0.077-1.98 | 2.70E-01 | 0.39033 | 0.077-1.98 | 2.70E-01 | 4.62E-03 | 0.434 | 0.720209 | 3.01E-01 | 0 |
| Bread intake | 1.310375 | 0.838-2.049 | 2.39E-01 | 1.310375 | 0.838-2.049 | 2.39E-01 | -4.00E-03 | 0.181 | 0.87739 | 1.22E-02 | 1 |
| lamb/mutton intake | 1.454514 | 0.777-2.721 | 2.43E-01 | 1.454514 | 0.777-2.721 | 2.43E-01 | -4.56E-03 | 0.132 | 0.807028 | 7.19E-02 | 0 |
| Hot drink temperature | 1.345689 | 0.665-2.725 | 4.10E-01 | 1.345689 | 0.665-2.725 | 4.10E-01 | -2.67E-03 | 0.333 | 0.873634 | 1.96E-01 | 1 |
| Cheese intake | 0.820118 | 0.567-1.186 | 2.93E-01 | 0.820118 | 0.567-1.186 | 2.93E-01 | 1.16E-03 | 0.671 | 0.894407 | 5.75E-02 | 2 |
| Water intake | 0.980913 | 0.678-1.419 | 9.19E-01 | 0.980913 | 0.678-1.419 | 9.19E-01 | -5.79E-04 | 0.795 | 1.177575 | 9.83E-02 | 2 |
| Cereal intake | 1.308896 | 0.819-2.091 | 2.62E-01 | 1.308896 | 0.819-2.091 | 2.62E-01 | -4.26E-03 | 0.122 | 0.779801 | 1.47E-02 | 1 |
| Dried fruit intake | 0.659931 | 0.345-1.262 | 2.11E-01 | 0.659931 | 0.345-1.262 | 2.11E-01 | 2.12E-03 | 0.54 | 1.052782 | 5.89E-01 | 2 |
| Alcohol usually taken with meals | 0.849358 | 0.355-2.033 | 7.14E-01 | 0.849358 | 0.355-2.033 | 7.14E-01 | 4.30E-04 | 0.903 | 0.886969 | 1.87E-01 | 7 |
| Average weekly spirits intake | 1.332773 | 0.662-2.685 | 4.26E-01 | 1.332773 | 0.662-2.685 | 4.26E-01 | -7.17E-03 | 0.146 | 0.998067 | 9.78E-01 | 1 |
| Non-oily fish intake | 1.601644 | 0.808-3.177 | 1.83E-01 | 1.601644 | 0.808-3.177 | 1.83E-01 | -5.33E-03 | 0.153 | 0.966968 | 7.97E-01 | 1 |
| Salad / raw vegetable intake | 0.975162 | 0.485-1.959 | 9.44E-01 | 0.975162 | 0.485-1.959 | 9.44E-01 | -7.08E-04 | 0.829 | 0.88853 | 2.20E-01 | 2 |
| Oily fish intake | 0.935974 | 0.578-1.515 | 7.88E-01 | 0.935974 | 0.578-1.515 | 7.88E-01 | -6.29E-04 | 0.834 | 1.033263 | 5.76E-01 | 0 |
| Beef intake | 0.940772 | 0.482-1.837 | 8.59E-01 | 0.940772 | 0.482-1.837 | 8.59E-01 | 2.57E-03 | 0.491 | 1.012384 | 8.53E-01 | 0 |
| Fresh fruit intake | 0.78492 | 0.388-1.586 | 5.01E-01 | 0.78492 | 0.388-1.586 | 5.01E-01 | -5.36E-05 | 0.985 | 1.061672 | 5.68E-01 | 1 |
| Average weekly beer plus cider intake | 1.279325 | 0.688-2.38 | 4.39E-01 | 1.279325 | 0.688-2.38 | 4.39E-01 | -2.04E-03 | 0.55 | 0.947474 | 5.81E-01 | 0 |
| Coffee intake | 1.284965 | 0.887-1.861 | 1.87E-01 | 1.284965 | 0.887-1.861 | 1.87E-01 | -5.06E-03 | 0.028 | 1.046614 | 3.11E-01 | 3 |
| Average weekly red wine intake | 1.141249 | 0.742-1.755 | 5.49E-01 | 1.141249 | 0.742-1.755 | 5.49E-01 | -2.18E-03 | 0.453 | 0.975762 | 7.05E-01 | 0 |
| pork intake | 0.705642 | 0.303-1.641 | 4.21E-01 | 0.705642 | 0.303-1.641 | 4.21E-01 | 3.42E-03 | 0.388 | 0.937572 | 5.03E-01 | 1 |
| Average weekly champagne plus white wine intake | 0.49561 | 0.224-1.096 | 8.96E-02 | 0.49561 | 0.224-1.096 | 8.96E-02 | 8.78E-03 | 0.082 | 0.933741 | 4.46E-01 | 5 |
| Tea intake | 1.04837 | 0.78-1.409 | 7.55E-01 | 1.04837 | 0.78-1.409 | 7.55E-01 | -2.30E-04 | 0.917 | 0.951571 | 4.25E-01 | 1 |
| Processed meat intake | 1.054966 | 0.654-1.701 | 8.27E-01 | 1.054966 | 0.654-1.701 | 8.27E-01 | -5.78E-04 | 0.861 | 0.887769 | 6.35E-02 | 4 |
| Poultry intake | 1.013201 | 0.495-2.075 | 9.71E-01 | 1.013201 | 0.495-2.075 | 9.71E-01 | 9.14E-04 | 0.822 | 0.798215 | 4.51E-03 | 3 |
| Cooked vegetable intake | 0.510378 | 0.259-1.008 | 5.59E-02 | 0.510378 | 0.259-1.008 | 5.59E-02 | 6.31E-03 | 0.067 | 0.891215 | 2.29E-01 | 2 |
| Salt added to food | 0.814448 | 0.608-1.091 | 1.70E-01 | 0.814448 | 0.608-1.091 | 1.70E-01 | 3.34E-03 | 0.07 | 1.080567 | 4.66E-01 | 2 |
| Milk type used: Full cream | 0.69018 | 0.049-9.711 | 7.86E-01 | 0.69018 | 0.049-9.711 | 7.86E-01 | 1.60E-03 | 0.75 | 1.038526 | 9.15E-01 | 0 |
| Milk type used: Semi-skimmed | 6.329093 | 0.503-79.668 | 1.81E-01 | 6.329093 | 0.503-79.668 | 1.81E-01 | -9.33E-03 | 0.296 | 1.665983 | 2.65E-01 | 0 |
| Milk type used: Skimmed | 2.286618 | 0.265-19.72 | 4.59E-01 | 2.286618 | 0.265-19.72 | 4.59E-01 | -6.70E-03 | 0.306 | 0.770097 | 4.69E-01 | 0 |
| Milk type used: Soya | 0.227993 | 0.007-7.683 | 4.19E-01 | 0.227993 | 0.007-7.683 | 4.19E-01 | 2.63E-03 | 0.627 | 0.51904 | 3.23E-01 | 0 |
| Milk type used: Other type of milk | 1.869551 | 0.004-907.46 | 8.46E-01 | 1.869551 | 0.004-907.46 | 8.46E-01 | -1.84E-03 | 0.786 | 0.880824 | 9.37E-01 | 0 |
| Milk type used: Never/rarely have milk | 0.467161 | 0.002-95.018 | 7.82E-01 | 0.467161 | 0.002-95.018 | 7.82E-01 | 3.15E-03 | 0.687 | 0.778144 | 7.85E-01 | 1 |
| Bread type: White | 1.736736 | 0.439-6.873 | 4.33E-01 | 1.736736 | 0.439-6.873 | 4.33E-01 | -2.68E-03 | 0.552 | 1.157008 | 3.39E-01 | 2 |
| Bread type: Brown | 7.181974 | 0.598-86.309 | 1.41E-01 | 7.181974 | 0.598-86.309 | 1.41E-01 | -1.25E-02 | 0.062 | 0.652528 | 2.28E-01 | 0 |
| Bread type: Wholemeal or wholegrain | 2.036531 | 0.45-9.226 | 3.59E-01 | 2.036531 | 0.45-9.226 | 3.59E-01 | -6.72E-03 | 0.208 | 0.744954 | 5.69E-02 | 1 |
| Bread type: Other type of bread | 0.108292 | 0.004-2.74 | 1.94E-01 | 0.108292 | 0.004-2.74 | 1.94E-01 | 8.31E-03 | 0.188 | 0.870777 | 8.32E-01 | 0 |
| Cereal type: Bran cereal (e.g. All Bran, Branflakes) | 1.947578 | 0.175-21.64 | 6.01E-01 | 1.947578 | 0.175-21.64 | 6.01E-01 | -8.53E-04 | 0.919 | 1.736773 | 3.25E-01 | 0 |
| Cereal type: Biscuit cereal (e.g. Weetabix) | 0.646997 | 0.129-3.245 | 6.01E-01 | 0.646997 | 0.129-3.245 | 6.01E-01 | 3.12E-03 | 0.591 | 0.975566 | 9.40E-01 | 0 |
| Cereal type: Oat cereal (e.g. Ready Brek, porridge) | 0.984073 | 0.144-6.733 | 9.87E-01 | 0.984073 | 0.144-6.733 | 9.87E-01 | -4.94E-04 | 0.947 | 0.752223 | 4.37E-01 | 1 |
| Cereal type: Muesli | 0.959273 | 0.211-4.367 | 9.57E-01 | 0.959273 | 0.211-4.367 | 9.57E-01 | 3.45E-04 | 0.945 | 0.919673 | 7.18E-01 | 1 |
| Cereal type: Other (e.g. Cornflakes, Frosties) | 1.494334 | 0.234-9.556 | 6.73E-01 | 1.494334 | 0.234-9.556 | 6.73E-01 | -3.59E-03 | 0.561 | 0.966961 | 9.02E-01 | 1 |
| Coffee type: Decaffeinated coffee (any type) | 1.127704 | 0.163-7.82 | 9.05E-01 | 1.127704 | 0.163-7.82 | 9.05E-01 | 7.40E-03 | 0.395 | 3.139398 | 2.37E-03 | 1 |
| Coffee type: Instant coffee | 1.953532 | 0.4-9.536 | 4.14E-01 | 1.953532 | 0.4-9.536 | 4.14E-01 | -5.36E-03 | 0.426 | 1.046862 | 8.47E-01 | 0 |
| Coffee type: Ground coffee (include espresso, filter etc) | 0.33387 | 0.107-1.045 | 6.26E-02 | 0.33387 | 0.107-1.045 | 6.26E-02 | 6.40E-03 | 0.121 | 0.799785 | 1.76E-01 | 2 |
| Coffee type: Other type of coffee | 3.327053 | 0.908-1.626 | 8.24E-01 | 3.327053 | 0.0001-6.77E+04 | 8.24E-01 | 6.98E-04 | 0.969 | 3.964266 | 5.92E-01 | 0 |

**Table S11.** Causality of genetically determined 45 dietary habits on BC(ER+) of breast cancer in sensitivity analysis.

| **Exposure** | **Weighted median** | | | **MR Egger** | | | | | **MR Presso** | | |
| --- | --- | --- | --- | --- | --- | --- | --- | --- | --- | --- | --- |
|  | **OR** | **95%CI** | **Pval** | **OR** | **95%CI** | **Pval** | **Intercept** | **Intercept**  **Pval** | **OR** | **Pval** | **N outliers** |
| Age when last ate meat | 1.019969 | 0.891-1.168 | 7.74E-01 | 1.167428 | 0.826-1.65 | 4.04E-01 | -9.56E-03 | 4.91E-01 | 1.034305 | 4.27E-01 | 0 |
| Average weekly fortified wine intake | 0.563998 | 0.251-1.27 | 1.67E-01 | 0.407705 | 0.087-1.913 | 2.69E-01 | 4.59E-03 | 4.15E-01 | 0.747498 | 3.14E-02 | 0 |
| Bread intake | 0.978835 | 0.798-1.201 | 8.37E-01 | 1.37635 | 0.845-2.243 | 2.03E-01 | -4.70E-03 | 1.49E-01 | 0.871057 | 1.06E-01 | 1 |
| lamb/mutton intake | 1.010304 | 0.765-1.334 | 9.42E-01 | 1.336079 | 0.666-2.681 | 4.17E-01 | -3.57E-03 | 2.88E-01 | 0.838168 | 1.66E-01 | 0 |
| Hot drink temperature | 0.972178 | 0.747-1.266 | 8.34E-01 | 1.616282 | 0.73-3.580 | 2.38E-01 | -4.12E-03 | 1.84E-01 | 0.897282 | 3.96E-01 | 0 |
| Cheese intake | 0.895462 | 0.776-1.033 | 1.30E-01 | 0.905615 | 0.605-1.356 | 6.31E-01 | -4.11E-04 | 8.91E-01 | 0.940562 | 3.90E-01 | 0 |
| Water intake | 0.964753 | 0.791-1.176 | 7.22E-01 | 0.971944 | 0.639-1.479 | 8.95E-01 | -2.54E-04 | 9.20E-01 | 1.194976 | 1.07E-01 | 0 |
| Cereal intake | 0.954389 | 0.785-1.16 | 6.39E-01 | 1.523042 | 0.924-2.511 | 1.01E-01 | -5.10E-03 | 8.27E-02 | 0.789573 | 2.78E-02 | 0 |
| Dried fruit intake | 0.833139 | 0.671-1.035 | 9.87E-02 | 0.679373 | 0.334-1.38 | 2.87E-01 | 2.33E-03 | 5.40E-01 | 1.069198 | 5.69E-01 | 0 |
| Alcohol usually taken with meals | 0.954832 | 0.726-1.256 | 7.41E-01 | 0.754464 | 0.274-2.075 | 5.86E-01 | 1.87E-03 | 6.47E-01 | 0.869932 | 1.47E-01 | 2 |
| Average weekly spirits intake | 0.853303 | 0.621-1.172 | 3.27E-01 | 1.314351 | 0.617-2.799 | 4.83E-01 | -6.44E-03 | 2.25E-01 | 1.041709 | 6.36E-01 | 2 |
| Non-oily fish intake | 0.90634 | 0.635-1.294 | 5.88E-01 | 1.18101 | 0.526-2.65 | 6.88E-01 | -2.66E-03 | 5.44E-01 | 0.93183 | 6.01E-01 | 0 |
| Salad / raw vegetable intake | 0.916671 | 0.665-1.263 | 5.95E-01 | 0.928723 | 0.41-2.105 | 8.60E-01 | -3.33E-04 | 9.31E-01 | 1.03997 | 7.49E-01 | 1 |
| Oily fish intake | 0.932412 | 0.782-1.112 | 4.37E-01 | 1.039923 | 0.591-1.831 | 8.92E-01 | -1.27E-03 | 7.20E-01 | 1.028514 | 6.46E-01 | 1 |
| Beef intake | 1.12178 | 0.849-1.482 | 4.19E-01 | 0.7169 | 0.355-1.449 | 3.56E-01 | 5.84E-03 | 1.39E-01 | 0.968123 | 6.83E-01 | 0 |
| Fresh fruit intake | 0.762728 | 0.553-1.052 | 9.85E-02 | 0.576506 | 0.279-1.19 | 1.39E-01 | 2.60E-03 | 3.76E-01 | 1.177267 | 1.50E-01 | 0 |
| Average weekly beer plus cider intake | 0.839632 | 0.635-1.111 | 2.21E-01 | 1.231412 | 0.597-2.541 | 5.75E-01 | -1.60E-03 | 6.88E-01 | 1.02947 | 7.90E-01 | 0 |
| Coffee intake | 0.833421 | 0.644-1.079 | 1.66E-01 | 1.220666 | 0.807-1.846 | 3.47E-01 | -4.64E-03 | 7.21E-02 | 1.029422 | 5.85E-01 | 0 |
| Average weekly red wine intake | 1.037577 | 0.824-1.307 | 7.54E-01 | 1.282711 | 0.728-2.26 | 3.91E-01 | -3.89E-03 | 3.11E-01 | 0.972679 | 6.96E-01 | 0 |
| pork intake | 0.926426 | 0.659-1.301 | 6.59E-01 | 0.651726 | 0.282-1.505 | 3.19E-01 | 3.46E-03 | 3.81E-01 | 0.930876 | 5.10E-01 | 0 |
| Average weekly champagne plus white wine intake | 0.892886 | 0.652-1.223 | 4.80E-01 | 0.444024 | 0.183-1.075 | 7.83E-02 | 1.14E-02 | 4.38E-02 | 0.954024 | 6.51E-01 | 3 |
| Tea intake | 1.088273 | 0.914-1.296 | 3.43E-01 | 1.009519 | 0.735-1.386 | 9.53E-01 | 6.08E-05 | 9.80E-01 | 0.952411 | 4.96E-01 | 0 |
| Processed meat intake | 0.889632 | 0.734-1.078 | 2.33E-01 | 0.864152 | 0.488-1.531 | 6.18E-01 | 1.59E-03 | 6.86E-01 | 0.976918 | 7.46E-01 | 1 |
| Poultry intake | 1.215421 | 0.911-1.622 | 1.85E-01 | 0.906303 | 0.43-1.909 | 7.96E-01 | 3.04E-03 | 4.73E-01 | 0.829338 | 3.61E-02 | 1 |
| Cooked vegetable intake | 0.945398 | 0.701-1.275 | 7.13E-01 | 0.427033 | 0.202-0.901 | 2.80E-02 | 8.98E-03 | 1.83E-02 | 0.97023 | 7.78E-01 | 3 |
| Salt added to food | 1.035756 | 0.897-1.196 | 6.31E-01 | 0.902292 | 0.648-1.257 | 5.44E-01 | 1.70E-03 | 4.12E-01 | 0.931875 | 6.11E-01 | 0 |
| Milk type used: Full cream | 1.069201 | 0.253-4.518 | 9.27E-01 | 0.3441 | 0.015-7.849 | 5.10E-01 | 3.85E-03 | 5.19E-01 | 0.917628 | 8.52E-01 | 0 |
| Milk type used: Semi-skimmed | 1.817355 | 0.556-5.936 | 3.23E-01 | 6.376497 | 0.466-87.297 | 1.93E-01 | -9.55E-03 | 3.02E-01 | 1.63267 | 3.00E-01 | 0 |
| Milk type used: Skimmed | 1.396761 | 0.541-3.603 | 4.90E-01 | 6.32561 | 0.818-48.916 | 8.99E-02 | -1.18E-02 | 6.40E-02 | 0.931539 | 8.45E-01 | 0 |
| Milk type used: Soya | 0.839778 | 0.092-7.7 | 8.77E-01 | 2.075769 | 0.024-177.423 | 7.51E-01 | -4.02E-03 | 5.58E-01 | 0.592617 | 5.32E-01 | 0 |
| Milk type used: Other type of milk | 0.194241 | 0.001-37.02 | 5.41E-01 | 0.927183 | 0.0002-4184 | 9.86E-01 | -1.35E-03 | 8.84E-01 | 0.536187 | 7.77E-01 | 0 |
| Milk type used: Never/rarely have milk | 1.087112 | 0.1-11.831 | 9.45E-01 | 0.514522 | 0.002-135.39 | 8.18E-01 | 4.51E-03 | 5.83E-01 | 2.237867 | 4.55E-01 | 0 |
| Bread type: White | 1.077421 | 0.718-1.618 | 7.19E-01 | 2.597258 | 0.584-11.56 | 2.13E-01 | -5.08E-03 | 3.02E-01 | 1.252057 | 1.85E-01 | 1 |
| Bread type: Brown | 0.479824 | 0.123-1.87 | 2.90E-01 | 2.70264 | 0.148-49.20 | 5.11E-01 | -1.02E-02 | 1.82E-01 | 0.388406 | 7.12E-02 | 0 |
| Bread type: Wholemeal or wholegrain | 0.898446 | 0.595-1.357 | 6.11E-01 | 2.645583 | 0.471-14.875 | 2.73E-01 | -8.45E-03 | 1.68E-01 | 0.810136 | 2.26E-01 | 2 |
| Bread type: Other type of bread | 0.509979 | 0.072-3.625 | 5.01E-01 | 0.361799 | 0.009-14.06 | 5.93E-01 | 4.57E-03 | 5.16E-01 | 1.135957 | 8.23E-01 | 0 |
| Cereal type: Bran cereal (e.g. All Bran, Branflakes) | 1.873766 | 0.487-7.205 | 3.61E-01 | 3.266118 | 0.292-36.494 | 3.62E-01 | -8.03E-04 | 9.23E-01 | 2.927092 | 6.66E-02 | 0 |
| Cereal type: Biscuit cereal (e.g. Weetabix) | 1.33188 | 0.505-3.51 | 5.62E-01 | 1.240049 | 0.146-10.55 | 8.45E-01 | -1.34E-03 | 8.62E-01 | 1.29669 | 5.24E-01 | 1 |
| Cereal type: Oat cereal (e.g. Ready Brek, porridge) | 0.698363 | 0.25-1.954 | 4.94E-01 | 1.496652 | 0.177-12.68 | 7.16E-01 | -9.61E-04 | 9.08E-01 | 1.332267 | 5.17E-01 | 0 |
| Cereal type: Muesli | 1.420481 | 0.757-2.666 | 2.74E-01 | 1.019556 | 0.211-4.922 | 9.81E-01 | 1.01E-03 | 8.47E-01 | 1.065198 | 7.92E-01 | 1 |
| Cereal type: Other (e.g. Cornflakes, Frosties) | 0.89335 | 0.455-1.752 | 7.43E-01 | 1.439117 | 0.206-10.074 | 7.16E-01 | -5.20E-03 | 4.23E-01 | 0.6699 | 1.87E-01 | 0 |
| Coffee type: Decaffeinated coffee (any type) | 2.793211 | 0.979-7.971 | 5.49E-02 | 1.413214 | 0.111-18.05 | 7.94E-01 | 4.51E-03 | 6.91E-01 | 2.437055 | 2.81E-02 | 2 |
| Coffee type: Instant coffee | 1.167218 | 0.62-2.197 | 6.32E-01 | 1.844873 | 0.285-11.93 | 5.25E-01 | -6.61E-03 | 4.05E-01 | 0.854767 | 5.75E-01 |  |
| Coffee type: Ground coffee (include espresso, filter etc) | 0.802188 | 0.522-1.232 | 3.14E-01 | 0.264816 | 0.072-0.971 | 4.77E-02 | 8.59E-03 | 6.79E-02 | 0.896903 | 5.30E-01 | 3 |
| Coffee type: Other type of coffee | 4.775221 | 0.018-1272 | 5.83E-01 | 0.735312 | 3.07E-06-1.76E+05 | 9.64E-01 | 6.54E-03 | 7.75E-01 | 3.782502 | 6.80E-01 | 0 |

**Table S12.** Causality of genetically determined 45 dietary habits on BC(ER-) of breast cancer in sensitivity analysis.

| **Exposure** | **Weighted median** | | | **MR Egger** | | | | | **MR Presso** | | |
| --- | --- | --- | --- | --- | --- | --- | --- | --- | --- | --- | --- |
|  | **OR** | **95%CI** | **Pval** | **OR** | **95%CI** | **Pval** | **Intercept** | **Intercept**  **Pval** | **OR** | **Pval** | **N outliers** |
| Age when last ate meat | 1.344745 | 1.085-1.666 | 6.80E-03 | 1.756181 | 1.022-3.017 | 7.18E-02 | -2.23E-02 | 3.10E-01 | 1.322145 | 2.64E-03 | 0 |
| Average weekly fortified wine intake | 0.520228 | 0.14-1.935 | 3.29E-01 | 0.117035 | 0.005-2.577 | 1.89E-01 | 1.29E-02 | 2.51E-01 | 0.463254 | 1.47E-01 | 1 |
| Bread intake | 0.985731 | 0.717-1.356 | 9.30E-01 | 1.239483 | 0.543-2.831 | 6.11E-01 | -3.46E-03 | 5.28E-01 | 0.784146 | 1.58E-03 | 0 |
| lamb/mutton intake | 1.014748 | 0.681-1.513 | 9.43E-01 | 1.350208 | 0.518-3.52 | 5.40E-01 | -3.38E-03 | 4.63E-01 | 0.619503 | 3.61E-02 | 1 |
| Hot drink temperature | 1.130779 | 0.765-1.671 | 5.37E-01 | 0.768692 | 0.258-2.293 | 6.38E-01 | 3.68E-03 | 3.88E-01 | 0.935437 | 7.00E-01 | 0 |
| Cheese intake | 0.911261 | 0.74-1.122 | 3.82E-01 | 0.767797 | 0.447-1.318 | 3.39E-01 | 3.16E-04 | 9.37E-01 | 0.922623 | 4.22E-01 | 1 |
| Water intake | 0.834572 | 0.601-1.158 | 2.79E-01 | 0.816299 | 0.439-1.519 | 5.23E-01 | 1.34E-03 | 7.21E-01 | 0.97037 | 8.62E-01 | 0 |
| Cereal intake | 0.779859 | 0.588-1.035 | 8.46E-02 | 0.869 | 0.438-1.725 | 6.89E-01 | -1.48E-03 | 7.12E-01 | 0.825854 | 2.30E-01 | 0 |
| Dried fruit intake | 0.697839 | 0.512-0.951 | 2.27E-02 | 0.86453 | 0.358-2.09 | 7.47E-01 | -1.89E-03 | 6.90E-01 | 1.037726 | 8.21E-01 | 0 |
| Alcohol usually taken with meals | 0.743473 | 0.487-1.134 | 1.69E-01 | 1.026701 | 0.228-4.627 | 9.73E-01 | -2.35E-03 | 6.99E-01 | 0.790053 | 7.13E-02 | 0 |
| Average weekly spirits intake | 0.515908 | 0.307-0.866 | 1.23E-02 | 1.454014 | 0.337-6.278 | 6.19E-01 | -1.03E-02 | 3.10E-01 | 0.80469 | 7.70E-02 | 0 |
| Non-oily fish intake | 1.232987 | 0.734-2.07 | 4.28E-01 | 3.222301 | 1.116-9.303 | 3.45E-02 | -1.13E-02 | 5.13E-02 | 1.035144 | 8.52E-01 | 0 |
| Salad / raw vegetable intake | 1.192161 | 0.743-1.912 | 4.66E-01 | 0.692949 | 0.224-2.147 | 5.26E-01 | 2.90E-03 | 5.87E-01 | 0.727303 | 1.09E-01 | 0 |
| Oily fish intake | 0.886074 | 0.675-1.162 | 3.82E-01 | 1.26931 | 0.58-2.779 | 5.52E-01 | -3.53E-03 | 4.77E-01 | 0.988744 | 9.07E-01 | 0 |
| Beef intake | 1.01004 | 0.671-1.52 | 9.62E-01 | 1.651003 | 0.53-5.139 | 3.89E-01 | -6.07E-03 | 3.39E-01 | 1.278618 | 2.14E-02 | 0 |
| Fresh fruit intake | 0.66889 | 0.424-1.054 | 8.30E-02 | 0.705907 | 0.24-2.078 | 5.28E-01 | 1.30E-03 | 7.67E-01 | 1.214754 | 2.11E-01 | 0 |
| Average weekly beer plus cider intake | 0.940458 | 0.615-1.438 | 7.77E-01 | 0.898772 | 0.328-2.466 | 8.36E-01 | 1.63E-03 | 7.69E-01 | 0.957127 | 7.82E-01 | 0 |
| Coffee intake | 1.125924 | 0.732-1.733 | 5.90E-01 | 1.333587 | 0.784-2.269 | 2.91E-01 | -7.12E-03 | 3.11E-02 | 1.124518 | 1.54E-01 | 1 |
| Average weekly red wine intake | 0.583731 | 0.42-0.811 | 1.36E-03 | 0.596666 | 0.285-1.249 | 1.74E-01 | 4.15E-03 | 4.04E-01 | 0.959521 | 7.28E-01 | 0 |
| pork intake | 1.363051 | 0.807-2.303 | 2.47E-01 | 0.551064 | 0.167-1.815 | 3.30E-01 | 6.00E-03 | 2.80E-01 | 0.958034 | 7.70E-01 | 0 |
| Average weekly champagne plus white wine intake | 0.604842 | 0.378-0.967 | 3.56E-02 | 1.058805 | 0.253-4.426 | 9.38E-01 | -4.75E-03 | 5.96E-01 | 1.224735 | 1.68E-01 | 0 |
| Tea intake | 1.136237 | 0.841-1.536 | 4.06E-01 | 1.22657 | 0.747-2.013 | 4.21E-01 | -2.98E-03 | 4.24E-01 | 0.908605 | 3.63E-01 | 0 |
| Processed meat intake | 1.438387 | 1.076-1.923 | 1.41E-02 | 1.895549 | 0.877-4.097 | 1.07E-01 | -5.46E-03 | 3.01E-01 | 0.767532 | 7.16E-03 | 0 |
| Poultry intake | 1.075688 | 0.696-1.663 | 7.43E-01 | 1.439108 | 0.510-4.061 | 4.94E-01 | -1.97E-03 | 7.39E-01 | 0.726505 | 6.08E-03 | 0 |
| Cooked vegetable intake | 0.997029 | 0.643-1.547 | 9.89E-01 | 0.32321 | 0.106-0.986 | 5.02E-02 | 1.10E-02 | 5.04E-02 | 0.74272 | 7.89E-02 | 1 |
| Salt added to food | 1.023768 | 0.813-1.288 | 8.41E-01 | 0.770719 | 0.458-1.296 | 3.27E-01 | 4.51E-03 | 1.67E-01 | 1.169254 | 4.00E-01 | 0 |
| Milk type used: Full cream | 3.102323 | 0.32-30.043 | 3.28E-01 | 2.732797 | 0.021-356.5 | 6.89E-01 | -6.20E-04 | 9.46E-01 | 2.331339 | 2.56E-01 | 0 |
| Milk type used: Semi-skimmed | 0.94671 | 0.162-5.536 | 9.52E-01 | 27.0049 | 0.663-1100 | 1.09E-01 | -2.12E-02 | 1.17E-01 | 1.304314 | 6.65E-01 | 0 |
| Milk type used: Skimmed | 0.403652 | 0.087-1.872 | 2.47E-01 | 0.544398 | 0.007-39.77 | 7.84E-01 | 7.96E-04 | 9.50E-01 | 0.621715 | 4.63E-01 | 0 |
| Milk type used: Soya | 0.337421 | 0.013-8.731 | 5.13E-01 | 0.147199 | 0-82.66 | 5.59E-01 | 5.30E-04 | 9.56E-01 | 0.17376 | 1.44E-01 | 0 |
| Milk type used: Other type of milk | 6.874649 | 0.003-15284 | 6.24E-01 | 8052.52 | 0.168-3.85E+08 | 1.30E-01 | -1.83E-02 | 1.36E-01 | 4.160983 | 5.57E-01 | 0 |
| Milk type used: Never/rarely have milk | 4.210488 | 0.112-157.81 | 4.37E-01 | 1.312746 | 0.001-258 | 9.45E-01 | 3.39E-03 | 7.60E-01 | 3.98321 | 3.43E-01 | 0 |
| Bread type: White | 1.733378 | 0.955-3.147 | 7.07E-02 | 0.515308 | 0.065-4.073 | 5.31E-01 | 7.91E-03 | 2.43E-01 | 1.72162 | 2.23E-02 | 0 |
| Bread type: Brown | 0.230939 | 0.021-2.489 | 2.27E-01 | 0.085007 | 0-42.063 | 4.47E-01 | 6.06E-04 | 9.69E-01 | 0.095556 | 3.23E-02 | 0 |
| Bread type: Wholemeal or wholegrain | 0.473763 | 0.254-0.883 | 1.87E-02 | 0.387604 | 0.039-3.829 | 4.20E-01 | 2.29E-03 | 7.77E-01 | 0.536646 | 1.01E-02 | 0 |
| Bread type: Other type of bread | 0.387043 | 0.016-9.521 | 5.61E-01 | 0.044097 | 0-48.728 | 3.94E-01 | 1.27E-02 | 3.44E-01 | 1.106526 | 9.41E-01 | 0 |
| Cereal type: Bran cereal (e.g. All Bran, Branflakes) | 6.43996 | 0.793-52.26 | 8.13E-02 | 7.637596 | 0.294-198 | 2.52E-01 | -6.66E-03 | 5.60E-01 | 3.124696 | 1.43E-01 | 0 |
| Cereal type: Biscuit cereal (e.g. Weetabix) | 0.957952 | 0.241-3.808 | 9.51E-01 | 0.790321 | 0.066-9.417 | 8.54E-01 | 1.93E-03 | 8.27E-01 | 1.021371 | 9.66E-01 | 0 |
| Cereal type: Oat cereal (e.g. Ready Brek, porridge) | 0.561809 | 0.131-2.405 | 4.37E-01 | 0.700756 | 0.038-13.026 | 8.14E-01 | -1.68E-03 | 8.82E-01 | 0.571043 | 3.51E-01 | 0 |
| Cereal type: Muesli | 0.529206 | 0.217-1.293 | 1.62E-01 | 0.813965 | 0.075-8.812 | 8.66E-01 | -1.15E-04 | 9.88E-01 | 0.704951 | 3.32E-01 | 0 |
| Cereal type: Other (e.g. Cornflakes, Frosties) | 1.972239 | 0.694-5.607 | 2.03E-01 | 0.39589 | 0.021-7.409 | 5.38E-01 | 7.33E-03 | 4.56E-01 | 1.385836 | 4.45E-01 | 1 |
| Coffee type: Decaffeinated coffee (any type) | 1.524431 | 0.313-7.426 | 6.02E-01 | 0.138436 | 0.007-2.80 | 2.16E-01 | 2.35E-02 | 9.35E-02 | 1.721632 | 4.04E-01 | 0 |
| Coffee type: Instant coffee | 1.601124 | 0.659-3.89 | 2.99E-01 | 23.79254 | 2.454-230.688 | 1.02E-02 | -2.14E-02 | 3.16E-02 | 1.958499 | 6.95E-02 | 0 |
| Coffee type: Ground coffee (include espresso, filter etc) | 0.748114 | 0.413-1.355 | 3.38E-01 | 0.807529 | 0.153-4.271 | 8.02E-01 | -2.96E-03 | 6.22E-01 | 0.53901 | 1.00E-02 | 0 |
| Coffee type: Other type of coffee | 22.49184 | 0.008-65500 | 4.44E-01 | 7948.453 | 0.028-2.25E+09 | 2.33E-01 | -2.23E-02 | 3.56E-01 | 28.01616 | 3.77E-01 | 0 |

**Table S13.** Causality of genetically determined 45 dietary habits on endometrial cancer in sensitivity analysis.

| **Exposure** | **Weighted median** | | | **MR Egger** | | | | | **MR Presso** | | |
| --- | --- | --- | --- | --- | --- | --- | --- | --- | --- | --- | --- |
|  | **OR** | **95%CI** | **Pval** | **OR** | **95%CI** | **Pval** | **Intercept** | **Intercept**  **Pval** | **OR** | **Pval** | **N outliers** |
| Age when last ate meat | 0.902148 | 0.680-1.197 | 4.76E-01 | 1.133198 | 0.427-3.007 | 8.07E-01 | -1.76E-02 | 6.41E-01 | 0.992831 | 9.54E-01 | 1 |
| Average weekly fortified wine intake | 2.883559 | 0.805-10.34 | 1.04E-01 | 0.511912 | 0.009-30.318 | 7.51E-01 | 1.23E-02 | 3.93E-01 | 2.883559 | 1.20E-01 | 0 |
| Bread intake | 0.927038 | 0.711-1.209 | 5.76E-01 | 0.647553 | 0.259-1.621 | 3.55E-01 | 4.92E-03 | 4.25E-01 | 0.763753 | 9.92E-03 | 0 |
| lamb/mutton intake | 0.799314 | 0.522-1.224 | 3.02E-01 | 1.101993 | 0.26-4.669 | 8.95E-01 | -3.15E-03 | 6.49E-01 | 0.74109 | 2.17E-01 | 0 |
| Hot drink temperature | 0.935292 | 0.682-1.283 | 6.78E-01 | 2.868599 | 0.847-9.711 | 9.18E-02 | -8.78E-03 | 6.35E-02 | 1.04244 | 8.57E-01 | 0 |
| Cheese intake | 0.763753 | 0.624-0.936 | 9.22E-03 | 0.882076 | 0.415-1.875 | 7.45E-01 | -2.16E-03 | 6.98E-01 | 1.065004 | 6.00E-01 | 0 |
| Water intake | 0.943607 | 0.712-1.250 | 6.86E-01 | 1.721518 | 0.732-4.05 | 2.15E-01 | -7.52E-03 | 1.47E-01 | 0.922848 | 6.85E-01 | 0 |
| Cereal intake | 0.826613 | 0.641-1.067 | 1.43E-01 | 1.27744 | 0.515-3.169 | 5.98E-01 | -5.18E-03 | 3.29E-01 | 1.261881 | 2.68E-01 | 0 |
| Dried fruit intake | 0.95368 | 0.717-1.269 | 7.45E-01 | 0.700783 | 0.226-2.17 | 5.38E-01 | 3.34E-03 | 5.82E-01 | 0.967335 | 8.69E-01 | 0 |
| Alcohol usually taken with meals | 1.037397 | 0.679-1.585 | 8.65E-01 | 0.442555 | 0.065-3.03 | 4.08E-01 | 6.85E-03 | 3.75E-01 | 1.213191 | 2.04E-01 | 0 |
| Average weekly spirits intake | 0.862082 | 0.521-1.426 | 5.63E-01 | 0.683959 | 0.127-3.684 | 6.61E-01 | 3.27E-03 | 7.79E-01 | 1.263349 | 1.39E-01 | 0 |
| Non-oily fish intake | 1.915353 | 1.171-3.132 | 9.61E-03 | 3.194998 | 0.616-16.58 | 1.72E-01 | -5.65E-03 | 5.25E-01 | 0.980656 | 9.39E-01 | 0 |
| Salad / raw vegetable intake | 1.04244 | 0.665-1.635 | 8.56E-01 | 0.502448 | 0.107-2.349 | 3.84E-01 | 6.98E-03 | 3.34E-01 | 0.702569 | 1.32E-01 | 0 |
| Oily fish intake | 1.065004 | 0.842-1.347 | 5.99E-01 | 1.446934 | 0.594-3.522 | 4.17E-01 | -3.97E-03 | 4.85E-01 | 0.927437 | 4.98E-01 | 0 |
| Beef intake | 0.922848 | 0.627-1.358 | 6.84E-01 | 0.476276 | 0.132-1.717 | 2.60E-01 | 7.57E-03 | 2.92E-01 | 1.052775 | 7.09E-01 | 0 |
| Fresh fruit intake | 1.261881 | 0.837-1.902 | 2.66E-01 | 0.865925 | 0.202-3.718 | 8.47E-01 | 3.13E-03 | 5.98E-01 | 0.830791 | 3.46E-01 | 0 |
| Average weekly beer plus cider intake | 0.967335 | 0.652-1.435 | 8.69E-01 | 0.351652 | 0.1-1.242 | 1.08E-01 | 1.15E-02 | 1.01E-01 | 1.405812 | 9.36E-02 | 0 |
| Coffee intake | 1.213191 | 0.902-1.632 | 2.02E-01 | 1.20319 | 0.633-2.288 | 5.74E-01 | 1.14E-04 | 9.77E-01 | 1.246573 | 2.93E-02 | 0 |
| Average weekly red wine intake | 1.263349 | 0.929-1.718 | 1.36E-01 | 1.198402 | 0.433-3.317 | 7.28E-01 | 7.21E-04 | 9.15E-01 | 0.927038 | 5.66E-01 | 0 |
| pork intake | 0.980656 | 0.596-1.613 | 9.39E-01 | 1.412493 | 0.279-7.156 | 6.78E-01 | -3.51E-03 | 6.44E-01 | 0.799314 | 3.04E-01 | 0 |
| Average weekly champagne plus white wine intake | 0.702569 | 0.448-1.103 | 1.25E-01 | 2.32655 | 0.412-13.138 | 3.44E-01 | -1.50E-02 | 1.67E-01 | 0.935292 | 6.74E-01 | 0 |
| Tea intake | 0.927437 | 0.746-1.153 | 4.97E-01 | 0.912428 | 0.514-1.618 | 7.54E-01 | 2.58E-04 | 9.52E-01 | 0.943607 | 6.86E-01 | 0 |
| Processed meat intake | 1.052775 | 0.804-1.379 | 7.09E-01 | 0.561612 | 0.2-1.574 | 2.75E-01 | 8.61E-03 | 2.18E-01 | 0.826613 | 1.45E-01 | 0 |
| Poultry intake | 0.830791 | 0.566-1.219 | 3.43E-01 | 0.622602 | 0.165-2.352 | 4.87E-01 | 3.32E-03 | 6.58E-01 | 0.95368 | 7.46E-01 | 0 |
| Cooked vegetable intake | 1.405812 | 0.943-2.095 | 9.43E-02 | 2.381945 | 0.532-10.655 | 2.59E-01 | -5.30E-03 | 4.76E-01 | 1.037397 | 8.65E-01 | 0 |
| Salt added to food | 1.246573 | 1.024-1.518 | 2.83E-02 | 0.858174 | 0.460-1.600 | 6.31E-01 | 4.83E-03 | 2.17E-01 | 1.915353 | 1.19E-02 | 0 |
| Milk type used: Full cream | 0.388788 | 0.038-3.988 | 4.26E-01 | 0.01602 | 6.8E-06-37.60 | 3.06E-01 | 1.20E-02 | 4.06E-01 | 0.388788 | 4.34E-01 | 0 |
| Milk type used: Semi-skimmed | 2.154078 | 0.328-14.17 | 4.25E-01 | 1.190592 | 0.004-315.969 | 9.52E-01 | 4.33E-03 | 8.28E-01 | 2.154078 | 4.38E-01 | 0 |
| Milk type used: Skimmed | 0.909216 | 0.226-3.657 | 8.93E-01 | 0.327106 | 0.001-76.74 | 6.91E-01 | 6.16E-03 | 7.07E-01 | 0.909216 | 8.94E-01 | 0 |
| Milk type used: Soya | 0.863277 | 0.026-29.00 | 9.35E-01 | 0.03145 | 1.6E-06-605.0 | 4.99E-01 | 1.05E-02 | 4.88E-01 | 0.863277 | 9.35E-01 | 0 |
| Milk type used: Other type of milk | 1.211455 | 0.001-2549 | 9.61E-01 | 0.00065 | 6.8E-11-6238 | 3.92E-01 | 1.70E-02 | 3.21E-01 | 1.211455 | 9.62E-01 | 0 |
| Milk type used: Never/rarely have milk | 0.759502 | 0.029-20.24 | 8.70E-01 | 64.38193 | 0.009-464325 | 3.69E-01 | -1.33E-02 | 3.04E-01 | 0.759502 | 8.59E-01 | 0 |
| Bread type: White | 1.878588 | 1.035-3.409 | 3.81E-02 | 0.115824 | 0.01-1.358 | 8.88E-02 | 1.86E-02 | 2.42E-02 | 1.878588 | 4.03E-02 | 0 |
| Bread type: Brown | 0.712563 | 0.103-4.951 | 7.32E-01 | 0.031966 | 0.0001-9.541 | 2.53E-01 | 1.64E-02 | 2.72E-01 | 0.712563 | 6.97E-01 | 0 |
| Bread type: Wholemeal or wholegrain | 1.215914 | 0.699-2.115 | 4.89E-01 | 1.457436 | 0.105-20.207 | 7.80E-01 | -1.29E-03 | 8.90E-01 | 1.215914 | 4.91E-01 | 0 |
| Bread type: Other type of bread | 0.007516 | 0.0002-0.264 | 7.07E-03 | 0.002945 | 1.6E-07-52.76 | 2.57E-01 | 3.77E-03 | 8.42E-01 | 0.007516 | 1.36E-02 | 0 |
| Cereal type: Bran cereal (e.g. All Bran, Branflakes) | 4.551808 | 0.675-30.70 | 1.20E-01 | 36.66046 | 0.348-3867 | 1.61E-01 | -1.45E-02 | 3.59E-01 | 4.551808 | 1.01E-01 | 0 |
| Cereal type: Biscuit cereal (e.g. Weetabix) | 0.820651 | 0.218-3.091 | 7.70E-01 | 3.257017 | 0.115-92.48 | 4.96E-01 | -1.05E-02 | 3.87E-01 | 0.820651 | 7.73E-01 | 0 |
| Cereal type: Oat cereal (e.g. Ready Brek, porridge) | 3.925515 | 0.747-20.62 | 1.06E-01 | 0.637221 | 0.005-75.33 | 8.55E-01 | 1.43E-02 | 4.35E-01 | 3.925515 | 1.22E-01 | 0 |
| Cereal type: Muesli | 0.49975 | 0.205-1.219 | 1.27E-01 | 0.641841 | 0.032-13.051 | 7.74E-01 | -1.67E-03 | 8.65E-01 | 0.413111 | 4.32E-02 | 1 |
| Cereal type: Other (e.g. Cornflakes, Frosties) | 1.078656 | 0.376-3.098 | 8.88E-01 | 3.766842 | 0.123-115.1 | 4.51E-01 | -8.66E-03 | 4.54E-01 | 1.340346 | 5.74E-01 | 1 |
| Coffee type: Decaffeinated coffee (any type) | 1.346654 | 0.265-6.850 | 7.20E-01 | 0.234975 | 0.003-19.72 | 5.31E-01 | 1.58E-02 | 4.19E-01 | 1.346654 | 7.25E-01 | 0 |
| Coffee type: Instant coffee | 0.484674 | 0.194-1.212 | 1.21E-01 | 0.485918 | 0.017-14.06 | 6.77E-01 | -2.17E-05 | 9.99E-01 | 0.484674 | 1.32E-01 | 0 |
| Coffee type: Ground coffee (include espresso, filter etc) | 0.418563 | 0.231-0.758 | 4.06E-03 | 0.365253 | 0.037-3.56 | 3.88E-01 | 9.76E-04 | 9.04E-01 | 0.418563 | 4.94E-03 | 0 |
| Coffee type: Other type of coffee | 0.249973 | 0.00005-1334 | 7.52E-01 | 0.000173 | 4.2E-12-7136 | 4.04E-01 | 2.49E-02 | 4.20E-01 | 0.249973 | 7.22E-01 | 0 |

**Table S14.** Causality of genetically determined 45 dietary habits on EH of endometrial cancer in sensitivity analysis.

| **Exposure** | **Weighted median** | | | **MR Egger** | | | | | **MR Presso** | | |
| --- | --- | --- | --- | --- | --- | --- | --- | --- | --- | --- | --- |
|  | **OR** | **95%CI** | **Pval** | **OR** | **95%CI** | **Pval** | **Intercept** | **Intercept**  **Pval** | **OR** | **Pval** | **N outliers** |
| Age when last ate meat | 0.873722 | 0.619-1.233 | 4.43E-01 | 0.982923 | 0.341-2.836 | 9.75E-01 | -1.14E-02 | 7.80E-01 | 0.847768 | 3.13E-01 | 0 |
| Average weekly fortified wine intake | 9.774113 | 1.158-82.474 | 3.62E-02 | 1.062203 | 0.009-128.9 | 9.81E-01 | 1.20E-02 | 4.74E-01 | 5.844611 | 2.45E-02 | 0 |
| Bread intake | 0.911605 | 0.574-1.447 | 6.95E-01 | 1.359696 | 0.437-4.229 | 5.97E-01 | -6.03E-03 | 4.27E-01 | 0.72487 | 1.42E-02 | 1 |
| lamb/mutton intake | 1.211942 | 0.617-2.38 | 5.77E-01 | 1.286315 | 0.225-7.342 | 7.77E-01 | -4.75E-03 | 5.69E-01 | 0.680722 | 1.72E-01 | 1 |
| Hot drink temperature | 0.872329 | 0.496-1.533 | 6.35E-01 | 2.532329 | 0.6-10.686 | 2.07E-01 | -6.85E-03 | 2.20E-01 | 0.973254 | 9.26E-01 | 0 |
| Cheese intake | 0.536101 | 0.378-0.76 | 4.62E-04 | 1.348003 | 0.518-3.51 | 5.41E-01 | -9.71E-03 | 1.70E-01 | 1.067626 | 6.44E-01 | 0 |
| Water intake | 1.326397 | 0.817-2.154 | 2.53E-01 | 1.826307 | 0.651-5.125 | 2.54E-01 | -7.63E-03 | 2.22E-01 | 0.932064 | 7.74E-01 | 0 |
| Cereal intake | 0.773532 | 0.5-1.197 | 2.49E-01 | 1.365608 | 0.43-4.335 | 5.98E-01 | -6.91E-03 | 3.04E-01 | 1.523456 | 9.40E-02 | 1 |
| Dried fruit intake | 1.053864 | 0.657-1.69 | 8.28E-01 | 0.827748 | 0.212-3.228 | 7.86E-01 | 1.68E-03 | 8.18E-01 | 1.022746 | 9.25E-01 | 0 |
| Alcohol usually taken with meals | 1.449931 | 0.754-2.788 | 2.65E-01 | 0.529761 | 0.051-5.541 | 5.97E-01 | 5.78E-03 | 5.39E-01 | 1.059157 | 7.56E-01 | 0 |
| Average weekly spirits intake | 0.598635 | 0.295-1.216 | 1.56E-01 | 0.470902 | 0.065-3.436 | 4.62E-01 | 8.04E-03 | 5.60E-01 | 0.953365 | 8.09E-01 | 0 |
| Non-oily fish intake | 2.635733 | 1.174-5.918 | 1.88E-02 | 7.599322 | 0.785-73.608 | 8.47E-02 | -1.57E-02 | 2.04E-01 | 0.822926 | 5.33E-01 | 0 |
| Salad / raw vegetable intake | 1.022759 | 0.489-2.138 | 9.52E-01 | 0.996255 | 0.147-6.764 | 9.97E-01 | -2.25E-04 | 9.80E-01 | 0.623062 | 8.72E-02 | 0 |
| Oily fish intake | 0.941722 | 0.643-1.379 | 7.58E-01 | 2.062846 | 0.724-5.876 | 1.77E-01 | -8.53E-03 | 2.03E-01 | 0.879637 | 3.66E-01 | 0 |
| Beef intake | 0.72443 | 0.386-1.358 | 3.15E-01 | 0.444276 | 0.09-2.185 | 3.21E-01 | 8.47E-03 | 3.41E-01 | 1.230198 | 2.12E-01 | 1 |
| Fresh fruit intake | 1.105577 | 0.56-2.182 | 7.72E-01 | 0.80512 | 0.136-4.75 | 8.11E-01 | 4.85E-03 | 5.02E-01 | 0.905966 | 6.95E-01 | 0 |
| Average weekly beer plus cider intake | 1.312803 | 0.696-2.475 | 4.00E-01 | 0.335671 | 0.075-1.496 | 1.55E-01 | 1.27E-02 | 1.27E-01 | 1.687959 | 6.21E-02 | 0 |
| Coffee intake | 0.80364 | 0.44-1.466 | 4.76E-01 | 0.902961 | 0.412-1.981 | 7.99E-01 | 2.19E-03 | 6.54E-01 | 1.247093 | 7.11E-02 | 0 |
| Average weekly red wine intake | 1.039257 | 0.626-1.724 | 8.81E-01 | 1.475841 | 0.393-5.548 | 5.66E-01 | -4.89E-03 | 5.79E-01 | 0.874535 | 4.25E-01 | 0 |
| pork intake | 0.579787 | 0.251-1.338 | 2.01E-01 | 2.718527 | 0.375-19.701 | 3.25E-01 | -1.15E-02 | 2.18E-01 | 0.791464 | 3.70E-01 | 0 |
| Average weekly champagne plus white wine intake | 0.582655 | 0.283-1.199 | 1.42E-01 | 5.960177 | 0.805-44.146 | 8.73E-02 | -2.83E-02 | 2.69E-02 | 1.058709 | 7.69E-01 | 0 |
| Tea intake | 0.799478 | 0.517-1.237 | 3.15E-01 | 0.674466 | 0.326-1.397 | 2.91E-01 | 4.19E-03 | 4.41E-01 | 0.945715 | 7.43E-01 | 1 |
| Processed meat intake | 1.228475 | 0.786-1.92 | 3.67E-01 | 0.654126 | 0.188-2.281 | 5.07E-01 | 8.04E-03 | 3.42E-01 | 0.762234 | 1.01E-01 |  |
| Poultry intake | 0.78366 | 0.403-1.525 | 4.73E-01 | 0.420288 | 0.078-2.271 | 3.17E-01 | 8.89E-03 | 3.54E-01 | 0.966484 | 8.46E-01 | 0 |
| Cooked vegetable intake | 1.631034 | 0.807-3.298 | 1.73E-01 | 3.535658 | 0.455-27.474 | 2.30E-01 | -7.44E-03 | 4.65E-01 | 1.082575 | 7.51E-01 | 2 |
| Salt added to food | 1.301934 | 0.923-1.837 | 1.33E-01 | 0.948932 | 0.444-2.027 | 8.92E-01 | 3.52E-03 | 4.58E-01 | 1.569962 | 1.95E-01 | 1 |
| Milk type used: Full cream | 0.080882 | 0.002-3.603 | 1.94E-01 | 2.944428 | 6.41E-05-1.4E+05 | 8.45E-01 | -5.58E-03 | 7.81E-01 | 2.196155 | 6.06E-01 | 1 |
| Milk type used: Semi-skimmed | 4.336705 | 0.297-63.28 | 2.83E-01 | 0.859587 | 0.002-320.024 | 9.61E-01 | 3.39E-03 | 8.72E-01 | 1.368644 | 7.62E-01 | 0 |
| Milk type used: Skimmed | 1.894565 | 0.222-16.203 | 5.60E-01 | 1.826375 | 0.002-1498 | 8.62E-01 | -3.26E-03 | 8.71E-01 | 0.44306 | 3.10E-01 | 1 |
| Milk type used: Soya | 0.299489 | 0.002-53.89 | 6.49E-01 | 2.383748 | 1.1E-05-5.3E+05 | 8.91E-01 | -6.50E-03 | 7.30E-01 | 0.306238 | 5.98E-01 | 0 |
| Milk type used: Other type of milk | 1.340893 | 3.7E-06-4.9E+05 | 9.64E-01 | 4.85E-05 | 3.1E-16-7.5E+06 | 4.67E-01 | 2.24E-02 | 4.11E-01 | 0.987965 | 9.98E-01 | 0 |
| Milk type used: Never/rarely have milk | 0.190992 | 0.001-38.918 | 5.42E-01 | 0.162123 | 4.3E-06-6175 | 7.39E-01 | 1.86E-03 | 9.02E-01 | 0.302904 | 5.25E-01 | 0 |
| Bread type: White | 2.441384 | 0.966-6.17 | 5.92E-02 | 0.077919 | 0.003-1.764 | 1.12E-01 | 2.10E-02 | 4.36E-02 | 2.163236 | 2.98E-02 | 3 |
| Bread type: Brown | 0.536722 | 0.024-12.147 | 6.96E-01 | 0.021355 | 2.4E-05-19.041 | 2.83E-01 | 1.94E-02 | 2.76E-01 | 0.842031 | 8.48E-01 | 0 |
| Bread type: Wholemeal or wholegrain | 1.279264 | 0.529-3.091 | 5.84E-01 | 6.461998 | 0.244-170.99 | 2.67E-01 | -1.33E-02 | 2.55E-01 | 0.821078 | 5.64E-01 | 1 |
| Bread type: Other type of bread | 0.004276 | 2.9E-05-0.631 | 3.23E-02 | 0.000138 | 8.3E-10-23.15 | 1.63E-01 | 1.43E-02 | 5.36E-01 | 0.008384 | 2.60E-02 | 0 |
| Cereal type: Bran cereal (e.g. All Bran, Branflakes) | 2.850619 | 0.122-66.336 | 5.14E-01 | 108.717 | 0.438-26973 | 1.27E-01 | -2.18E-02 | 2.50E-01 | 4.724344 | 1.96E-01 | 0 |
| Cereal type: Biscuit cereal (e.g. Weetabix) | 0.631506 | 0.077-5.152 | 6.68E-01 | 3.800328 | 0.09-159.938 | 4.91E-01 | -1.08E-02 | 4.24E-01 | 0.914846 | 9.01E-01 | 0 |
| Cereal type: Oat cereal (e.g. Ready Brek, porridge) | 1.253552 | 0.123-12.74 | 8.49E-01 | 1.016536 | 0.003-351.75 | 9.96E-01 | 1.15E-02 | 6.05E-01 | 2.049685 | 4.62E-01 | 1 |
| Cereal type: Muesli | 0.369414 | 0.103-1.322 | 1.26E-01 | 1.432695 | 0.042-49.164 | 8.43E-01 | -9.68E-03 | 4.00E-01 | 0.332108 | 4.29E-02 | 0 |
| Cereal type: Other (e.g. Cornflakes, Frosties) | 2.194399 | 0.491-9.817 | 3.04E-01 | 3.358359 | 0.06-188.155 | 5.58E-01 | -5.23E-03 | 7.00E-01 | 2.845211 | 6.00E-02 | 2 |
| Coffee type: Decaffeinated coffee (any type) | 0.309263 | 0.026-3.622 | 3.50E-01 | 0.059988 | 0.0004-9.17 | 2.90E-01 | 2.15E-02 | 3.33E-01 | 0.649295 | 6.56E-01 | 0 |
| Coffee type: Instant coffee | 0.53643 | 0.14-2.052 | 3.63E-01 | 0.196197 | 0.003-12.003 | 4.44E-01 | 1.06E-02 | 5.37E-01 | 0.691996 | 5.23E-01 | 0 |
| Coffee type: Ground coffee (include espresso, filter etc) | 0.278109 | 0.108-0.714 | 7.80E-03 | 0.442056 | 0.032-6.155 | 5.45E-01 | -1.29E-03 | 8.90E-01 | 0.369316 | 5.34E-03 | 0 |
| Coffee type: Other type of coffee | 1.506737 | 3.3E-06-6.8E+05 | 9.51E-01 | 8497679 | 1.3E-06-5.39E+19 | 3.49E-01 | -3.75E-02 | 4.92E-01 | 0.700979 | 9.56E-01 | 1 |

**Table S15.** Causality of genetically determined 45 dietary habits on NEH of endometrial cancer in sensitivity analysis.

| **Exposure** | **Weighted median** | | | **MR Egger** | | | | | **MR Presso** | | |
| --- | --- | --- | --- | --- | --- | --- | --- | --- | --- | --- | --- |
|  | **OR** | **95%CI** | **Pval** | **OR** | **95%CI** | **Pval** | **Intercept** | **Intercept**  **Pval** | **OR** | **Pval** | **N outliers** |
| Age when last ate meat | 0.682544 | 0.311-1.500 | 3.42E-01 | 2.721422 | 0.389-19.056 | 3.37E-01 | -9.73E-02 | 2.08E-01 | 0.76067 | 3.96E-01 | 0 |
| Average weekly fortified wine intake | 1.076362 | 0.009-135.9 | 9.76E-01 | 31.30474 | 0.00008-1.2E+07 | 6.06E-01 | -2.76E-02 | 5.34E-01 | 0.585934 | 6.45E-01 | 0 |
| Bread intake | 0.46229 | 0.137-1.557 | 2.13E-01 | 0.411156 | 0.02-8.61 | 5.68E-01 | 1.15E-02 | 5.73E-01 | 0.90199 | 6.05E-01 | 0 |
| lamb/mutton intake | 0.562324 | 0.119-2.655 | 4.67E-01 | 3.73669 | 0.096-146.073 | 4.82E-01 | -1.54E-02 | 3.84E-01 | 0.265264 | 6.13E-03 | 0 |
| Hot drink temperature | 1.064352 | 0.285-3.973 | 9.26E-01 | 5.888443 | 0.131-263.709 | 3.62E-01 | -1.27E-02 | 3.92E-01 | 1.042781 | 9.33E-01 | 0 |
| Cheese intake | 0.754608 | 0.352-1.618 | 4.69E-01 | 0.153471 | 0.018-1.282 | 8.54E-02 | 2.66E-02 | 9.19E-02 | 1.064021 | 8.18E-01 | 0 |
| Water intake | 1.008419 | 0.304-3.342 | 9.89E-01 | 6.06533 | 0.57-64.548 | 1.38E-01 | -2.60E-02 | 6.92E-02 | 1.304339 | 5.21E-01 | 0 |
| Cereal intake | 0.574389 | 0.194-1.702 | 3.17E-01 | 0.397054 | 0.024-6.612 | 5.21E-01 | 5.71E-03 | 7.28E-01 | 0.830026 | 6.89E-01 | 0 |
| Dried fruit intake | 0.714549 | 0.233-2.189 | 5.56E-01 | 0.18282 | 0.006-5.447 | 3.28E-01 | 1.47E-02 | 4.22E-01 | 0.799567 | 6.12E-01 | 0 |
| Alcohol usually taken with meals | 2.191221 | 0.459-10.45 | 3.25E-01 | 1.825688 | 0.009-380.6 | 8.25E-01 | -2.45E-03 | 9.07E-01 | 0.796905 | 4.83E-01 | 0 |
| Average weekly spirits intake | 0.23075 | 0.041-1.303 | 9.68E-02 | 0.419086 | 0.006-30.02 | 6.92E-01 | -6.41E-03 | 8.27E-01 | 1.351092 | 4.32E-01 | 0 |
| Non-oily fish intake | 1.033555 | 0.152-7.022 | 9.73E-01 | 1.230134 | 0.013-113.1 | 9.29E-01 | 1.01E-03 | 9.67E-01 | 1.149621 | 7.99E-01 | 0 |
| Salad / raw vegetable intake | 0.821606 | 0.142-4.760 | 8.26E-01 | 0.01943 | 0.0001-2.632 | 1.19E-01 | 3.71E-02 | 1.03E-01 | 0.568329 | 2.24E-01 | 0 |
| Oily fish intake | 1.106297 | 0.447-2.736 | 8.27E-01 | 0.390578 | 0.031-4.939 | 4.69E-01 | 1.30E-02 | 4.24E-01 | 0.779088 | 3.38E-01 | 0 |
| Beef intake | 0.789273 | 0.169-3.683 | 7.63E-01 | 0.478846 | 0.008-28.226 | 7.24E-01 | 1.12E-02 | 6.17E-01 | 0.832298 | 5.34E-01 | 0 |
| Fresh fruit intake | 0.558541 | 0.099-3.164 | 5.10E-01 | 0.133298 | 0.002-7.262 | 3.25E-01 | 1.53E-02 | 3.52E-01 | 0.596415 | 2.37E-01 | 0 |
| Average weekly beer plus cider intake | 0.576036 | 0.135-2.451 | 4.55E-01 | 0.130657 | 0.003-5.895 | 2.98E-01 | 1.99E-02 | 3.35E-01 | 1.192382 | 6.89E-01 | 0 |
| Coffee intake | 0.550387 | 0.117-2.580 | 4.49E-01 | 0.269604 | 0.044-1.636 | 1.57E-01 | 1.51E-02 | 1.87E-01 | 1.17667 | 4.51E-01 | 0 |
| Average weekly red wine intake | 2.255493 | 0.607-8.381 | 2.25E-01 | 3.275409 | 0.1-107.144 | 5.07E-01 | -1.18E-02 | 6.08E-01 | 0.955743 | 8.90E-01 | 0 |
| pork intake | 2.817295 | 0.429-18.50 | 2.81E-01 | 2.443399 | 0.021-284.8 | 7.14E-01 | -7.14E-03 | 7.46E-01 | 0.783788 | 5.53E-01 | 0 |
| Average weekly champagne plus white wine intake | 0.689064 | 0.112-4.243 | 6.88E-01 | 0.30815 | 0.002-58.272 | 6.62E-01 | 7.73E-03 | 8.14E-01 | 1.178327 | 6.53E-01 | 0 |
| Tea intake | 0.652931 | 0.214-1.994 | 4.54E-01 | 0.214877 | 0.042-1.107 | 6.85E-02 | 2.06E-02 | 9.75E-02 | 0.750379 | 3.50E-01 | 0 |
| Processed meat intake | 0.706488 | 0.234-2.129 | 5.37E-01 | 0.813815 | 0.033-20.102 | 9.00E-01 | 2.99E-04 | 9.89E-01 | 0.642537 | 1.37E-01 | 0 |
| Poultry intake | 0.450855 | 0.079-2.565 | 3.69E-01 | 0.729592 | 0.003-203.8 | 9.13E-01 | -2.20E-03 | 9.43E-01 | 0.707018 | 2.80E-01 | 0 |
| Cooked vegetable intake | 1.538283 | 0.263-8.988 | 6.33E-01 | 1.137546 | 0.003-386.3 | 9.66E-01 | 4.60E-04 | 9.87E-01 | 1.337934 | 5.81E-01 | 0 |
| Salt added to food | 1.168806 | 0.476-2.868 | 7.33E-01 | 0.668135 | 0.111-4.022 | 6.60E-01 | 7.34E-03 | 5.16E-01 | 1.348501 | 5.86E-01 | 0 |
| Milk type used: Full cream | 0.573155 | 0.0001-2847 | 8.98E-01 | 4416.281 | 2.9E-06-6.6E+12 | 4.44E-01 | -2.78E-02 | 4.74E-01 | 2.47317 | 6.60E-01 | 0 |
| Milk type used: Semi-skimmed | 60.38932 | 0.123-29670 | 1.95E-01 | 38.33757 | 3.6E-05-4.1E+07 | 6.16E-01 | -1.48E-02 | 7.58E-01 | 4.673611 | 4.64E-01 | 0 |
| Milk type used: Skimmed | 1.902037 | 0.013-287 | 8.02E-01 | 143.1582 | 5.8E-06-3.5E+09 | 5.74E-01 | -2.69E-02 | 6.02E-01 | 1.608086 | 7.29E-01 | 0 |
| Milk type used: Soya | 24454.37 | 0.05-1.2E+10 | 1.31E-01 | 20835458 | 2.3E-06-1.9E+20 | 2.85E-01 | -2.11E-02 | 6.48E-01 | 26234.02 | 9.03E-03 | 0 |
| Milk type used: Other type of milk | 16732186 | 4.0E-06-7.0E+19 | 2.62E-01 | 8617708 | 4.6E-14-1.6E+27 | 5.18E-01 | -2.16E-02 | 6.56E-01 | 576.5668 | 4.12E-01 | 0 |
| Milk type used: Never/rarely have milk | 0.023094 | 1.4E-08-36835 | 6.05E-01 | 7597.23 | 1.2E-11-4.7E+18 | 6.15E-01 | -3.00E-02 | 5.35E-01 | 0.215221 | 6.92E-01 | 0 |
| Bread type: White | 2.424394 | 0.275-21.389 | 4.25E-01 | 0.354505 | 0.0003-430.3 | 7.75E-01 | 9.58E-03 | 6.82E-01 | 1.509598 | 5.00E-01 | 0 |
| Bread type: Brown | 1.506534 | 0.001-3718 | 9.18E-01 | 3651.835 | 0.0001-1.1E+11 | 3.66E-01 | -4.14E-02 | 3.64E-01 | 1.600601 | 8.48E-01 | 0 |
| Bread type: Wholemeal or wholegrain | 1.419929 | 0.162-12.428 | 7.51E-01 | 1.871895 | 0.001-6251.242 | 8.80E-01 | -5.99E-03 | 8.37E-01 | 0.807786 | 7.36E-01 | 0 |
| Bread type: Other type of bread | 28.53328 | 0.001-1024779 | 5.31E-01 | 1.64E+08 | 0.174-1.5E+17 | 8.87E-02 | -7.55E-02 | 7.11E-02 | 1.219087 | 9.51E-01 | 0 |
| Cereal type: Bran cereal (e.g. All Bran, Branflakes) | 0.006733 | 1.5E-06-30.01 | 2.43E-01 | 0.024704 | 3.8E-10-1.6E+06 | 6.99E-01 | 3.61E-03 | 9.50E-01 | 0.043394 | 2.43E-01 | 0 |
| Cereal type: Biscuit cereal (e.g. Weetabix) | 0.19438 | 0.001-37.556 | 5.42E-01 | 157.5645 | 0.004-6.5E+06 | 3.61E-01 | -4.02E-02 | 2.75E-01 | 0.555075 | 7.04E-01 | 0 |
| Cereal type: Oat cereal (e.g. Ready Brek, porridge) | 3.390917 | 0.018-650.4 | 6.49E-01 | 11.96401 | 6.3E-06-2.3E+07 | 7.41E-01 | -1.17E-02 | 8.22E-01 | 2.366442 | 6.03E-01 | 0 |
| Cereal type: Muesli | 0.043242 | 0.002-1.051 | 5.36E-02 | 2.750336 | 0.0004-19649 | 8.24E-01 | -1.38E-02 | 6.29E-01 | 0.328399 | 2.10E-01 | 0 |
| Cereal type: Other (e.g. Cornflakes, Frosties) | 0.380885 | 0.012-12.02 | 5.84E-01 | 0.479065 | 6.4E-05-3608 | 8.72E-01 | 2.75E-03 | 9.25E-01 | 0.723576 | 7.59E-01 | 0 |
| Coffee type: Decaffeinated coffee (any type) | 44.76502 | 0.148-13499 | 1.92E-01 | 60.42971 | 0.001-4.8E+06 | 4.88E-01 | -1.98E-02 | 6.86E-01 | 6.629885 | 3.22E-01 | 0 |
| Coffee type: Instant coffee | 0.597633 | 0.018-20.08 | 7.74E-01 | 0.199795 | 1.3E-05-3097 | 7.46E-01 | 7.42E-03 | 8.52E-01 | 0.490259 | 5.19E-01 | 0 |
| Coffee type: Ground coffee (include espresso, filter etc) | 0.184402 | 0.021-1.601 | 1.25E-01 | 0.107412 | 0.0001-58.51 | 4.89E-01 | 5.27E-03 | 8.12E-01 | 0.225525 | 2.55E-02 | 0 |
| Coffee type: Other type of coffee | 85.83825 | 1.7E-13-4.3E+16 | 7.97E-01 | 79563584 | 1.2E-37-5.2E+52 | 7.63E-01 | -1.04E-01 | 7.70E-01 | 3.645748 | 9.24E-01 | 0 |

**Table S16.** Causality of genetically determined 45 dietary habits on LC in sensitivity analysis.

| **Exposure** | **Weighted median** | | | **MR Egger** | | | | | **MR Presso** | | |
| --- | --- | --- | --- | --- | --- | --- | --- | --- | --- | --- | --- |
|  | **OR** | **95%CI** | **Pval** | **OR** | **95%CI** | **Pval** | **Intercept** | **Intercept**  **Pval** | **OR** | **Pval** | **N outliers** |
| Age when last ate meat | 0.830288 | 0.676-1.369 | 8.30E-01 | 0.622372 | 0.199-1.950 | 4.43E-01 | 2.87E-02 | 5.22E-01 | 0.911194 | 4.77E-01 | 0 |
| Average weekly fortified wine intake | 0.415891 | 0.052-3.405 | 4.16E-01 | 0.751085 | 0.013-42.56 | 8.92E-01 | -3.92E-03 | 7.87E-01 | 0.447926 | 1.78E-01 | 0 |
| Bread intake | 0.577426 | 0.513-1.451 | 5.77E-01 | 0.721073 | 0.193-2.690 | 6.28E-01 | 3.60E-03 | 6.82E-01 | 0.94096 | 4.69E-01 | 0 |
| lamb/mutton intake | 0.905511 | 0.502-1.843 | 9.06E-01 | 1.253293 | 0.222-7.077 | 7.99E-01 | -2.07E-03 | 7.99E-01 | 1.086038 | 6.89E-01 | 0 |
| Hot drink temperature | 0.33674 | 0.407-1.36 | 3.37E-01 | 2.976518 | 0.567-15.61 | 1.99E-01 | -1.07E-02 | 1.07E-01 | 1.271983 | 2.20E-01 | 0 |
| Cheese intake | 0.419065 | 0.619-1.221 | 4.19E-01 | 0.594487 | 0.240-1.472 | 2.63E-01 | 7.00E-03 | 3.04E-01 | 0.807761 | 4.45E-02 | 0 |
| Water intake | 0.992052 | 0.577-1.724 | 9.92E-01 | 1.795989 | 0.661-4.882 | 2.54E-01 | -6.50E-03 | 2.84E-01 | 1.110866 | 5.26E-01 | 0 |
| Cereal intake | 0.337254 | 0.496-1.271 | 3.37E-01 | 0.642733 | 0.187-2.207 | 4.84E-01 | 2.92E-03 | 6.82E-01 | 0.953219 | 7.90E-01 | 0 |
| Dried fruit intake | 0.024343 | 0.313-0.923 | 2.43E-02 | 0.722724 | 0.166-3.140 | 6.66E-01 | 2.56E-04 | 9.74E-01 | 1.74067 | 4.76E-04 | 0 |
| Alcohol usually taken with meals | 0.007731 | 0.222-0.795 | 7.73E-03 | 1.629114 | 0.225-11.80 | 6.30E-01 | -1.04E-02 | 1.93E-01 | 1.218219 | 1.54E-01 | 0 |
| Average weekly spirits intake | 0.622084 | 0.390-1.757 | 6.22E-01 | 1.044033 | 0.168-6.487 | 9.63E-01 | 5.51E-04 | 9.65E-01 | 0.773964 | 9.17E-02 | 0 |
| Non-oily fish intake | 0.34936 | 0.312-1.509 | 3.49E-01 | 2.950371 | 0.564-15.42 | 2.06E-01 | -1.46E-02 | 1.14E-01 | 0.818076 | 2.65E-01 | 0 |
| Salad / raw vegetable intake | 0.369345 | 0.657-3.095 | 3.69E-01 | 2.767556 | 0.422-18.16 | 2.92E-01 | -7.51E-03 | 3.99E-01 | 0.829078 | 4.08E-01 | 0 |
| Oily fish intake | 0.321293 | 0.539-1.225 | 3.21E-01 | 0.814789 | 0.277-2.394 | 7.10E-01 | -1.14E-04 | 9.87E-01 | 1.000862 | 9.94E-01 | 0 |
| Beef intake | 0.256821 | 0.761-2.776 | 2.57E-01 | 4.184313 | 0.788-22.21 | 9.74E-02 | -1.50E-02 | 1.09E-01 | 1.473782 | 1.42E-03 | 0 |
| Fresh fruit intake | 0.862775 | 0.513-2.218 | 8.63E-01 | 3.898033 | 0.661-22.98 | 1.36E-01 | -1.18E-02 | 1.08E-01 | 0.969948 | 8.62E-01 | 0 |
| Average weekly beer plus cider intake | 0.077884 | 0.942-3.093 | 7.79E-02 | 6.08875 | 1.117-33.19 | 4.04E-02 | -1.40E-02 | 1.37E-01 | 0.893712 | 5.73E-01 | 0 |
| Coffee intake | 0.947632 | 0.515-2.031 | 9.48E-01 | 0.617643 | 0.289-1.319 | 2.17E-01 | 9.70E-03 | 4.95E-02 | 1.10941 | 2.78E-01 | 0 |
| Average weekly red wine intake | 0.478979 | 0.478-1.414 | 4.79E-01 | 0.837567 | 0.176-3.977 | 8.24E-01 | -1.03E-03 | 9.19E-01 | 0.940489 | 6.65E-01 | 0 |
| pork intake | 0.468151 | 0.324-1.678 | 4.68E-01 | 1.366365 | 0.195-9.592 | 7.55E-01 | -4.91E-03 | 5.89E-01 | 1.008475 | 9.58E-01 | 0 |
| Average weekly champagne plus white wine intake | 0.691158 | 0.383-1.890 | 6.91E-01 | 0.717207 | 0.066-7.833 | 7.87E-01 | 1.79E-03 | 9.03E-01 | 0.790465 | 9.98E-02 | 0 |
| Tea intake | 0.959568 | 0.613-1.673 | 9.60E-01 | 0.93632 | 0.462-1.897 | 8.55E-01 | 1.09E-03 | 8.40E-01 | 1.069907 | 5.88E-01 | 0 |
| Processed meat intake | 0.012911 | 1.134-2.893 | 1.29E-02 | 2.045046 | 0.513-8.151 | 3.13E-01 | -4.48E-03 | 6.33E-01 | 0.824659 | 1.09E-01 | 0 |
| Poultry intake | 0.954413 | 0.469-2.044 | 9.54E-01 | 0.79425 | 0.118-5.328 | 8.13E-01 | 2.28E-03 | 8.30E-01 | 0.739882 | 2.58E-02 | 0 |
| Cooked vegetable intake | 0.588932 | 0.385-1.718 | 5.89E-01 | 0.815011 | 0.093-7.174 | 8.54E-01 | 9.16E-04 | 9.32E-01 | 0.450243 | 5.01E-05 | 0 |
| Salt added to food | 0.561858 | 0.773-1.607 | 5.62E-01 | 1.313177 | 0.585-2.948 | 5.10E-01 | -2.19E-03 | 6.69E-01 | 0.823774 | 3.81E-01 | 0 |
| Milk type used: Full cream | 0.107654 | 0.511-911.61 | 1.08E-01 | 66.91132 | 0.003-1.6E+06 | 4.25E-01 | -6.74E-03 | 7.14E-01 | 10.63901 | 2.05E-02 | 0 |
| Milk type used: Semi-skimmed | 0.713022 | 0.034-10.157 | 7.13E-01 | 0.337816 | 0.001-103.5 | 7.21E-01 | 5.71E-03 | 7.96E-01 | 0.693613 | 4.90E-01 | 0 |
| Milk type used: Skimmed | 0.81496 | 0.109-5.730 | 8.15E-01 | 0.413308 | 0.006-28.772 | 6.87E-01 | 7.58E-03 | 5.74E-01 | 1.318999 | 5.89E-01 | 0 |
| Milk type used: Soya | 0.830566 | 0.002-170.69 | 8.31E-01 | 1.689715 | 8.5E-06-3.3E+05 | 9.34E-01 | -2.72E-03 | 8.88E-01 | 0.736451 | 7.85E-01 | 0 |
| Milk type used: Other type of milk | 0.466433 | 3.3E-08-2676 | 4.66E-01 | 0.002424 | 1.9E-10-2.9E+04 | 4.93E-01 | 2.31E-03 | 8.97E-01 | 0.006123 | 6.22E-02 | 0 |
| Milk type used: Never/rarely have milk | 0.799555 | 0.001-287 | 8.00E-01 | 555.2051 | 6.2E-05-4.9E+09 | 4.57E-01 | -1.67E-02 | 4.55E-01 | 1.336845 | 8.91E-01 | 0 |
| Bread type: White | 0.042765 | 1.034-7.331 | 4.28E-02 | 0.975153 | 0.044-21.53 | 9.87E-01 | 5.11E-03 | 6.22E-01 | 2.087144 | 1.23E-02 | 0 |
| Bread type: Brown | 0.442629 | 0.003-13.058 | 4.43E-01 | 0.108562 | 1.6E-06-7.0E+03 | 7.06E-01 | 6.64E-03 | 8.11E-01 | 0.416086 | 4.82E-01 | 0 |
| Bread type: Wholemeal or wholegrain | 0.271123 | 0.19-1.595 | 2.71E-01 | 1.383433 | 0.05-38.577 | 8.49E-01 | -5.08E-03 | 6.81E-01 | 0.69792 | 2.15E-01 | 0 |
| Bread type: Other type of bread | 0.563999 | 0.001-48.367 | 5.64E-01 | 0.199225 | 1.5E-07-2.5E+05 | 8.27E-01 | 3.19E-03 | 9.01E-01 | 0.472338 | 4.45E-01 | 0 |
| Cereal type: Bran cereal (e.g. All Bran, Branflakes) | 0.674182 | 0.018-13.646 | 6.74E-01 | 0.072719 | 0.0002-25.761 | 4.10E-01 | 1.73E-02 | 4.25E-01 | 0.717085 | 7.31E-01 | 0 |
| Cereal type: Biscuit cereal (e.g. Weetabix) | 0.735193 | 0.047-8.705 | 7.35E-01 | 5.240179 | 0.034-796.794 | 5.31E-01 | -1.42E-02 | 4.36E-01 | 0.791927 | 7.34E-01 | 0 |
| Cereal type: Oat cereal (e.g. Ready Brek, porridge) | 0.376209 | 0.021-4.313 | 3.76E-01 | 0.748802 | 0.002-236.726 | 9.24E-01 | -5.17E-03 | 8.11E-01 | 0.379516 | 1.91E-01 | 0 |
| Cereal type: Muesli | 0.832641 | 0.279-4.882 | 8.33E-01 | 1.668132 | 0.086-32.19 | 7.36E-01 | -4.92E-03 | 6.25E-01 | 0.829115 | 6.53E-01 | 0 |
| Cereal type: Other (e.g. Cornflakes, Frosties) | 0.130509 | 0.703-15.329 | 1.31E-01 | 12.54714 | 0.352-446.811 | 1.75E-01 | -1.07E-02 | 3.82E-01 | 2.728515 | 1.95E-02 | 0 |
| Coffee type: Decaffeinated coffee (any type) | 0.719033 | 0.163-13.841 | 7.19E-01 | 13.00903 | 0.3-563.2 | 2.02E-01 | -2.56E-02 | 1.47E-01 | 0.906731 | 8.95E-01 | 0 |
| Coffee type: Instant coffee | 0.859312 | 0.284-4.517 | 8.59E-01 | 0.81675 | 0.023-29.26 | 9.13E-01 | 7.17E-04 | 9.62E-01 | 0.888022 | 7.23E-01 | 0 |
| Coffee type: Ground coffee (include espresso, filter etc) | 0.155717 | 0.18-1.316 | 1.56E-01 | 1.527182 | 0.109-21.43 | 7.54E-01 | -6.83E-03 | 4.71E-01 | 0.594713 | 8.42E-02 | 0 |
| Coffee type: Other type of coffee | 0.110447 | 0.043-2.2E+13 | 1.10E-01 | 1.97E+07 | 2.0E-11-1.9E+25 | 5.72E-01 | -1.65E-02 | 8.86E-01 | NA | NA | NA |

**Table S17.** Causality of genetically determined 45 dietary habits on LUAD in sensitivity analysis.

| **Exposure** | **Weighted median** | | | **MR Egger** | | | | | **MR Presso** | | |
| --- | --- | --- | --- | --- | --- | --- | --- | --- | --- | --- | --- |
|  | **OR** | **95%CI** | **Pval** | **OR** | **95%CI** | **Pval** | **Intercept** | **Intercept**  **Pval** | **OR** | **Pval** | **N outliers** |
| Age when last ate meat | 0.97718 | 0.648-1.474 | 0.912343 | 0.281365 | 0.050-1.596 | 1.95E-01 | 9.33E-02 | 1.91E-01 | 0.97718 | 9.08E-01 | 0 |
| Average weekly fortified wine intake | 0.737984 | 0.048-11.37 | 0.827624 | 0.652427 | 0.0005-872.7 | 9.10E-01 | 9.48E-04 | 9.72E-01 | 0.737984 | 7.75E-01 | 0 |
| Bread intake | 0.669732 | 0.378-1.188 | 0.170389 | 0.810551 | 0.101-6.533 | 8.44E-01 | -2.54E-03 | 8.53E-01 | 1.069729 | 5.44E-01 | 0 |
| lamb/mutton intake | 0.946446 | 0.443-2.022 | 0.887006 | 2.060465 | 0.122-34.697 | 6.17E-01 | -7.42E-03 | 5.77E-01 | 0.796935 | 3.96E-01 | 0 |
| Hot drink temperature | 0.943061 | 0.481-1.848 | 0.864345 | 1.894319 | 0.134-26.683 | 6.37E-01 | -5.63E-03 | 5.94E-01 | 1.106459 | 6.93E-01 | 0 |
| Cheese intake | 1.069729 | 0.721-1.587 | 0.737684 | 1.043592 | 0.221-4.931 | 9.57E-01 | 3.74E-04 | 9.74E-01 | 0.791513 | 1.08E-01 | 0 |
| Water intake | 1.158885 | 0.686-1.958 | 0.58163 | 1.386216 | 0.306-6.283 | 6.73E-01 | -2.32E-03 | 8.05E-01 | 1.299655 | 2.50E-01 | 0 |
| Cereal intake | 1.153008 | 0.691-1.925 | 0.586209 | 3.533946 | 0.536-23.314 | 1.92E-01 | -1.32E-02 | 2.29E-01 | 0.878097 | 6.43E-01 | 0 |
| Dried fruit intake | 0.864678 | 0.489-1.528 | 0.616727 | 0.749979 | 0.091-6.202 | 7.90E-01 | 1.59E-03 | 8.91E-01 | 1.361022 | 2.32E-01 | 0 |
| Alcohol usually taken with meals | 0.715926 | 0.352-1.457 | 0.35653 | 0.664914 | 0.023-18.9 | 8.12E-01 | 5.98E-04 | 9.65E-01 | 1.135826 | 4.83E-01 | 0 |
| Average weekly spirits intake | 0.796935 | 0.341-1.861 | 0.599948 | 0.182197 | 0.005-6.588 | 3.59E-01 | 1.98E-02 | 4.13E-01 | 0.828123 | 3.36E-01 | 0 |
| Non-oily fish intake | 0.603495 | 0.236-1.545 | 0.292371 | 0.752374 | 0.047-11.945 | 8.41E-01 | -2.52E-03 | 8.69E-01 | 0.648922 | 1.40E-01 | 0 |
| Salad / raw vegetable intake | 1.106459 | 0.419-2.923 | 0.838246 | 0.805495 | 0.042-15.494 | 8.87E-01 | 3.26E-03 | 8.25E-01 | 0.687067 | 2.15E-01 | 0 |
| Oily fish intake | 0.791513 | 0.502-1.247 | 0.313685 | 0.570925 | 0.098-3.332 | 5.35E-01 | 4.26E-03 | 7.08E-01 | 0.816143 | 1.01E-01 | 0 |
| Beef intake | 1.299655 | 0.619-2.730 | 0.488872 | 1.006921 | 0.08-12.677 | 9.96E-01 | 2.86E-03 | 8.37E-01 | 1.205976 | 2.72E-01 | 0 |
| Fresh fruit intake | 0.878097 | 0.404-1.908 | 0.742742 | 2.44492 | 0.155-38.622 | 5.27E-01 | -8.65E-03 | 4.51E-01 | 1.24081 | 4.06E-01 | 0 |
| Average weekly beer plus cider intake | 1.361022 | 0.647-2.865 | 0.416978 | 3.616104 | 0.173-75.738 | 4.11E-01 | -1.07E-02 | 5.19E-01 | 0.897874 | 6.54E-01 | 0 |
| Coffee intake | 1.135826 | 0.644-2.005 | 0.660352 | 0.787089 | 0.242-2.564 | 6.92E-01 | 5.33E-03 | 4.90E-01 | 0.979206 | 8.72E-01 | 0 |
| Average weekly red wine intake | 0.828123 | 0.450-1.522 | 0.543818 | 0.222033 | 0.017-2.847 | 2.52E-01 | 1.70E-02 | 3.01E-01 | 0.669732 | 3.69E-02 | 0 |
| pork intake | 0.648922 | 0.249-1.694 | 0.377018 | 2.075631 | 0.074-58.27 | 6.70E-01 | -1.11E-02 | 4.79E-01 | 0.946446 | 7.89E-01 | 0 |
| Average weekly champagne plus white wine intake | 0.687067 | 0.274-1.722 | 0.42333 | 4.600228 | 0.106-198.8 | 4.33E-01 | -2.34E-02 | 3.15E-01 | 0.943061 | 7.56E-01 | 0 |
| Tea intake | 0.816143 | 0.528-1.261 | 0.359966 | 0.650727 | 0.222-1.907 | 4.36E-01 | 3.75E-03 | 6.53E-01 | 1.158885 | 3.67E-01 | 0 |
| Processed meat intake | 1.205976 | 0.683-2.129 | 0.518527 | 2.026003 | 0.180-22.80 | 5.69E-01 | -7.09E-03 | 6.67E-01 | 1.153008 | 3.92E-01 | 0 |
| Poultry intake | 1.24081 | 0.537-2.870 | 0.613994 | 0.918153 | 0.056-14.98 | 9.52E-01 | 3.55E-03 | 8.26E-01 | 0.864678 | 4.24E-01 | 0 |
| Cooked vegetable intake | 0.897874 | 0.380-2.124 | 0.8063 | 0.642674 | 0.02-20.792 | 8.04E-01 | 3.33E-03 | 8.46E-01 | 0.715926 | 2.09E-01 | 0 |
| Salt added to food | 0.979206 | 0.661-1.450 | 0.91642 | 0.613004 | 0.164-2.286 | 4.67E-01 | 6.11E-03 | 4.66E-01 | 0.603495 | 8.27E-02 | 0 |
| Milk type used: Full cream | 5.579043 | 0.061-511.8 | 0.455919 | 0.084475 | 6.0E-08-1.2E+05 | 7.37E-01 | 1.61E-02 | 5.49E-01 | 5.579043 | 1.95E-01 | 0 |
| Milk type used: Semi-skimmed | 1.058866 | 0.046-24.47 | 0.97152 | 412.7663 | 0.077-2.2E+06 | 2.03E-01 | -4.78E-02 | 1.78E-01 | 1.058866 | 9.55E-01 | 0 |
| Milk type used: Skimmed | 0.226667 | 0.022-2.359 | 0.214254 | 0.027177 | 3.8E-05-19.22 | 2.95E-01 | 1.40E-02 | 5.06E-01 | 0.226667 | 8.72E-02 | 0 |
| Milk type used: Soya | 0.19164 | 0.0001-202.4 | 0.641864 | 0.000428 | 1.6E-14-1.2E+07 | 5.39E-01 | 1.82E-02 | 6.12E-01 | 0.19164 | 4.25E-01 | 0 |
| Milk type used: Other type of milk | 0.128438 | 1.1E-08-1.6E+06 | 0.805233 | 0.003432 | 6.8E-15-1.7E+09 | 6.97E-01 | 1.05E-02 | 7.54E-01 | 0.128438 | 5.96E-01 | 0 |
| Milk type used: Never/rarely have milk | 0.875858 | 0.0003-2449 | 0.973885 | 1556.323 | 4.1E-08-5.9E+13 | 5.69E-01 | -2.18E-02 | 5.40E-01 | 0.875858 | 9.59E-01 | 0 |
| Bread type: White | 1.440599 | 0.470-4.413 | 0.522752 | 0.211878 | 0.002-24.26 | 5.23E-01 | 1.28E-02 | 4.17E-01 | 1.440599 | 3.07E-01 | 0 |
| Bread type: Brown | 0.314499 | 0.002-50.15 | 0.654846 | 0.002319 | 4.5E-11-1.2E+05 | 5.25E-01 | 2.58E-02 | 5.90E-01 | 0.314499 | 5.25E-01 | 0 |
| Bread type: Wholemeal or wholegrain | 1.059086 | 0.341-3.285 | 0.920828 | 2.756773 | 0.016-483.488 | 7.02E-01 | -6.93E-03 | 7.11E-01 | 1.059086 | 8.84E-01 | 0 |
| Bread type: Other type of bread | 3.737478 | 0.003-5315.9 | 0.721891 | 0.013971 | 5.1E-12-3.8E+07 | 7.09E-01 | 2.10E-02 | 6.06E-01 | 3.737478 | 5.05E-01 | 0 |
| Cereal type: Bran cereal (e.g. All Bran, Branflakes) | 0.16249 | 0.003-7.731 | 0.356467 | 0.203527 | 0.0001-413.9 | 6.94E-01 | -1.78E-03 | 9.48E-01 | 0.16249 | 1.79E-01 | 0 |
| Cereal type: Biscuit cereal (e.g. Weetabix) | 0.654303 | 0.023-18.85 | 0.804592 | 8.968567 | 0.002-50637 | 6.31E-01 | -2.03E-02 | 5.35E-01 | 0.654303 | 7.30E-01 | 0 |
| Cereal type: Oat cereal (e.g. Ready Brek, porridge) | 1.066054 | 0.049-23.37 | 0.967611 | 0.008074 | 2.0E-06-32.50 | 2.88E-01 | 3.79E-02 | 2.49E-01 | 1.066054 | 9.49E-01 | 0 |
| Cereal type: Muesli | 0.686553 | 0.151-3.120 | 0.626332 | 0.469944 | 0.006-36.18 | 7.35E-01 | 2.69E-03 | 8.56E-01 | 0.686553 | 4.14E-01 | 0 |
| Cereal type: Other (e.g. Cornflakes, Frosties) | 1.645637 | 0.325-8.335 | 0.547318 | 1.06853 | 0.007-173.3 | 9.80E-01 | 3.00E-03 | 8.62E-01 | 1.645637 | 3.99E-01 | 0 |
| Coffee type: Decaffeinated coffee (any type) | 0.269588 | 0.02-3.559 | 0.319406 | 0.609444 | 0.001-502.5 | 8.87E-01 | -7.32E-03 | 8.01E-01 | 0.269588 | 6.53E-02 | 0 |
| Coffee type: Instant coffee | 2.077055 | 0.425-10.15 | 0.36663 | 0.758827 | 0.004-134.4 | 9.18E-01 | 8.81E-03 | 6.92E-01 | 2.077055 | 1.98E-01 | 0 |
| Coffee type: Ground coffee (include espresso, filter etc) | 0.602398 | 0.198-1.835 | 0.372474 | 0.843293 | 0.009-75.68 | 9.41E-01 | -2.39E-03 | 8.80E-01 | 0.602398 | 2.21E-01 | 0 |
| Coffee type: Other type of coffee | 208244.3 | 0.005-9.1E+12 | 0.172421 | 1617.653 | 1.6E-17-1.65E+23 | 7.83E-01 | 2.57E-02 | 8.44E-01 | 208244.3 | 4.67E-02 | 0 |

**Table S18.** Causality of genetically determined 45 dietary habits on LUSC in sensitivity analysis.

| **Exposure** | **Weighted median** | | | **MR Egger** | | | | | **MR Presso** | | |
| --- | --- | --- | --- | --- | --- | --- | --- | --- | --- | --- | --- |
|  | **OR** | **95%CI** | **Pval** | **OR** | **95%CI** | **Pval** | **Intercept** | **Intercept**  **Pval** | **OR** | **Pval** | **N outliers** |
| Age when last ate meat | 0.89183 | 0.526-1.511 | 6.71E-01 | 1.140412 | 0.203-6.391 | 8.85E-01 | -1.46E-02 | 8.28E-01 | 0.940558 | 7.16E-01 | 0 |
| Average weekly fortified wine intake | 0.184706 | 0.006-6.065 | 3.43E-01 | 0.074884 | 2.6E-05-219 | 5.38E-01 | 1.13E-02 | 6.86E-01 | 0.368091 | 2.66E-01 | 0 |
| Bread intake | 0.637123 | 0.283-1.436 | 2.77E-01 | 0.680552 | 0.084-5.482 | 7.19E-01 | 4.91E-04 | 9.73E-01 | 0.959144 | 7.32E-01 | 0 |
| lamb/mutton intake | 0.883225 | 0.300-2.599 | 8.22E-01 | 0.87505 | 0.043-17.76 | 9.31E-01 | 1.03E-03 | 9.41E-01 | 1.426048 | 2.57E-01 | 0 |
| Hot drink temperature | 0.546503 | 0.214-1.393 | 2.06E-01 | 0.665936 | 0.047-9.402 | 7.64E-01 | 3.25E-03 | 7.57E-01 | 0.799394 | 3.39E-01 | 0 |
| Cheese intake | 0.875431 | 0.524-1.462 | 6.11E-01 | 0.54705 | 0.131-2.290 | 4.10E-01 | 8.50E-03 | 4.27E-01 | 0.837888 | 2.35E-01 | 0 |
| Water intake | 0.956485 | 0.45-2.034 | 9.08E-01 | 1.711672 | 0.402-7.285 | 4.69E-01 | -6.00E-03 | 5.14E-01 | 1.254297 | 3.03E-01 | 0 |
| Cereal intake | 0.790377 | 0.392-1.593 | 5.11E-01 | 2.966783 | 0.409-21.54 | 2.85E-01 | -1.54E-02 | 1.83E-01 | 0.804303 | 3.93E-01 | 0 |
| Dried fruit intake | 0.41693 | 0.182-0.955 | 3.85E-02 | 0.63573 | 0.066-6.130 | 6.96E-01 | -2.55E-04 | 9.83E-01 | 1.72252 | 2.13E-02 | 0 |
| Alcohol usually taken with meals | 0.421891 | 0.152-1.168 | 9.66E-02 | 3.124263 | 0.119-82.00 | 4.96E-01 | -1.53E-02 | 2.42E-01 | 0.917527 | 6.35E-01 | 0 |
| Average weekly spirits intake | 2.263124 | 0.661-7.747 | 1.93E-01 | 0.736082 | 0.016-33.44 | 8.76E-01 | 9.08E-03 | 7.29E-01 | 1.035386 | 8.72E-01 | 0 |
| Non-oily fish intake | 2.263716 | 0.557-9.196 | 2.53E-01 | 0.578956 | 0.008-44.51 | 8.07E-01 | 7.44E-03 | 7.51E-01 | 0.863167 | 6.06E-01 | 0 |
| Salad / raw vegetable intake | 0.72317 | 0.216-2.426 | 6.00E-01 | 1.503584 | 0.073-30.96 | 7.92E-01 | -6.18E-03 | 6.68E-01 | 0.87724 | 6.15E-01 | 0 |
| Oily fish intake | 0.638708 | 0.329-1.241 | 1.86E-01 | 1.72328 | 0.309-9.613 | 5.36E-01 | -9.63E-03 | 3.94E-01 | 0.870052 | 3.75E-01 | 0 |
| Beef intake | 1.089016 | 0.390-3.041 | 8.71E-01 | 2.846331 | 0.147-54.97 | 4.91E-01 | -8.90E-03 | 5.76E-01 | 1.096053 | 5.78E-01 | 0 |
| Fresh fruit intake | 0.82119 | 0.247-2.726 | 7.48E-01 | 4.701103 | 0.311-71.09 | 2.67E-01 | -1.51E-02 | 1.86E-01 | 1.23394 | 4.24E-01 | 0 |
| Average weekly beer plus cider intake | 1.42007 | 0.523-3.856 | 4.91E-01 | 6.284783 | 0.709-55.74 | 1.04E-01 | -1.51E-02 | 2.22E-01 | 1.228775 | 3.62E-01 | 0 |
| Coffee intake | 1.128802 | 0.453-2.812 | 7.95E-01 | 0.727845 | 0.235-2.254 | 5.83E-01 | 3.40E-03 | 6.43E-01 | 1.043778 | 7.26E-01 | 0 |
| Average weekly red wine intake | 0.849055 | 0.361-1.997 | 7.08E-01 | 2.707475 | 0.450-16.30 | 2.81E-01 | -1.35E-02 | 2.68E-01 | 0.704543 | 2.81E-02 | 0 |
| pork intake | 0.98832 | 0.254-3.841 | 9.86E-01 | 1.367228 | 0.031-59.35 | 8.72E-01 | -4.36E-03 | 8.04E-01 | 0.976368 | 9.07E-01 | 0 |
| Average weekly champagne plus white wine intake | 1.015913 | 0.296-3.491 | 9.80E-01 | 0.608548 | 0.009-41.34 | 8.19E-01 | 4.50E-03 | 8.63E-01 | 0.99867 | 9.95E-01 | 0 |
| Tea intake | 0.887242 | 0.45-1.751 | 7.30E-01 | 0.650547 | 0.224-1.890 | 4.32E-01 | 5.00E-03 | 5.55E-01 | 1.09144 | 6.08E-01 | 0 |
| Processed meat intake | 1.187734 | 0.561-2.514 | 6.53E-01 | 3.628573 | 0.343-38.43 | 2.88E-01 | -1.61E-02 | 3.09E-01 | 0.804434 | 1.80E-01 | 0 |
| Poultry intake | 1.205891 | 0.351-4.143 | 7.66E-01 | 0.51654 | 0.02-13.493 | 6.94E-01 | 1.01E-02 | 5.89E-01 | 0.621129 | 1.28E-02 | 0 |
| Cooked vegetable intake | 1.075337 | 0.348-3.326 | 9.00E-01 | 3.043531 | 0.094-98.08 | 5.32E-01 | -8.88E-03 | 5.99E-01 | 0.461484 | 4.16E-03 | 0 |
| Salt added to food | 1.144301 | 0.648-2.021 | 6.42E-01 | 1.352428 | 0.379-4.825 | 6.42E-01 | -3.39E-03 | 6.75E-01 | 1.147578 | 6.34E-01 | 0 |
| Milk type used: Full cream | 1.804689 | 0.004-810.203 | 8.50E-01 | 1125.324 | 0.0002-7.72E+09 | 3.95E-01 | -2.17E-02 | 4.68E-01 | 3.693841 | 2.74E-01 | 0 |
| Milk type used: Semi-skimmed | 22.0372 | 0.123-3950 | 2.43E-01 | 2170.231 | 0.002-2.8E+09 | 3.45E-01 | -3.77E-02 | 4.70E-01 | 9.411488 | 1.71E-01 | 0 |
| Milk type used: Skimmed | 5.090446 | 0.155-167.36 | 3.61E-01 | 0.90302 | 0.00004-19168 | 9.84E-01 | 3.36E-03 | 9.14E-01 | 1.545986 | 6.30E-01 | 0 |
| Milk type used: Soya | 0.021864 | 1.5E-06-309 | 4.33E-01 | 0.001922 | 6.4E-14-5.73E+07 | 6.21E-01 | 1.11E-02 | 7.51E-01 | 0.087485 | 1.34E-01 | 0 |
| Milk type used: Other type of milk | 171.9871 | 4.5E-06-6.5E+09 | 5.63E-01 | 1660.64 | 1.1E-08-2.6E+14 | 5.91E-01 | -1.12E-02 | 7.16E-01 | 24.63286 | 4.57E-01 | 0 |
| Milk type used: Never/rarely have milk | 0.010228 | 5.98E-07-174 | 3.57E-01 | 0.003676 | 2.4E-14-5.6E+08 | 6.79E-01 | 5.75E-03 | 8.65E-01 | 0.032907 | 1.33E-01 | 0 |
| Bread type: White | 1.587832 | 0.343-7.341 | 5.54E-01 | 6.545203 | 0.04-1061 | 4.72E-01 | -8.61E-03 | 6.16E-01 | 1.831926 | 1.13E-01 | 0 |
| Bread type: Brown | 0.301384 | 0.0001-497.8 | 7.51E-01 | 0.008106 | 2.6E-11-2.6E+06 | 6.50E-01 | 2.22E-02 | 6.72E-01 | 0.591977 | 8.10E-01 | 0 |
| Bread type: Wholemeal or wholegrain | 1.58172 | 0.341-7.326 | 5.58E-01 | 1.180741 | 0.004-310.3 | 9.54E-01 | -9.78E-04 | 9.61E-01 | 1.031459 | 9.32E-01 | 0 |
| Bread type: Other type of bread | 0.056927 | 3.7E-06-870 | 5.60E-01 | 0.000217 | 4.6E-13-103262 | 4.32E-01 | 2.62E-02 | 4.91E-01 | 0.199769 | 5.73E-01 | 0 |
| Cereal type: Bran cereal (e.g. All Bran, Branflakes) | 0.860273 | 0.004-174.4 | 9.56E-01 | 1.154615 | 2.7E-05-48547 | 9.80E-01 | 5.12E-04 | 9.88E-01 | 1.249352 | 8.95E-01 | 0 |
| Cereal type: Biscuit cereal (e.g. Weetabix) | 3.003886 | 0.052-172.1 | 5.94E-01 | 121.2557 | 0.03-49735 | 2.80E-01 | -3.63E-02 | 2.83E-01 | 1.44102 | 7.36E-01 | 0 |
| Cereal type: Oat cereal (e.g. Ready Brek, porridge) | 0.790797 | 0.01-59.87 | 9.15E-01 | 72.28328 | 0.001-5376871 | 4.83E-01 | -3.04E-02 | 4.62E-01 | 1.004924 | 9.97E-01 | 0 |
| Cereal type: Muesli | 1.534913 | 0.178-13.24 | 6.97E-01 | 1.670367 | 0.017-163.422 | 8.28E-01 | -4.01E-03 | 8.02E-01 | 0.959728 | 9.37E-01 | 0 |
| Cereal type: Other (e.g. Cornflakes, Frosties) | 6.397627 | 0.557-73.4 | 1.36E-01 | 160.3534 | 0.535-48032 | 9.09E-02 | -2.20E-02 | 2.61E-01 | 6.892497 | 2.13E-03 | 0 |
| Coffee type: Decaffeinated coffee (any type) | 1.477096 | 0.046-47.14 | 8.25E-01 | 13.35396 | 0.039-4517 | 4.00E-01 | -2.32E-02 | 4.14E-01 | 1.423567 | 6.71E-01 | 0 |
| Coffee type: Instant coffee | 0.434707 | 0.04-4.744 | 4.94E-01 | 0.548803 | 0.002-159.805 | 8.38E-01 | 5.84E-03 | 8.14E-01 | 1.058904 | 9.32E-01 | 0 |
| Coffee type: Ground coffee (include espresso, filter etc) | 0.808602 | 0.172-3.804 | 7.88E-01 | 1.804563 | 0.014-237.136 | 8.13E-01 | -6.90E-03 | 6.86E-01 | 0.674514 | 2.53E-01 | 0 |
| Coffee type: Other type of coffee | 21533.76 | 1.98E-08-2.3E+16 | 4.80E-01 | 89.06722 | 1.1E-60-7.5E+63 | 9.61E-01 | 6.72E-03 | 9.85E-01 | NA | NA | NA |

**Table S19.** Causality of genetically determined 45 dietary habits on OC in sensitivity analysis.

| **Exposure** | **Weighted median** | | | **MR Egger** | | | | | **MR Presso** | | |
| --- | --- | --- | --- | --- | --- | --- | --- | --- | --- | --- | --- |
|  | **OR** | **95%CI** | **Pval** | **OR** | **95%CI** | **Pval** | **Intercept** | **Intercept**  **Pval** | **OR** | **Pval** | **N outliers** |
| Age when last ate meat | 0.967855 | 0.753-1.245 | 7.99E-01 | 0.783866 | 0.421-1.461 | 4.63E-01 | 1.53E-02 | 5.42E-01 | 0.949916 | 5.93E-01 | 0 |
| Average weekly fortified wine intake | 0.774242 | 0.183-3.284 | 7.29E-01 | 0.649603 | 0.044-9.537 | 7.56E-01 | -7.13E-04 | 9.42E-01 | 0.591843 | 3.18E-01 | 0 |
| Bread intake | 1.123595 | 0.790-1.598 | 5.16E-01 | 1.029098 | 0.434-2.440 | 9.48E-01 | 1.01E-03 | 8.60E-01 | 0.864834 | 4.86E-02 | 0 |
| lamb/mutton intake | 0.932561 | 0.590-1.474 | 7.65E-01 | 0.917152 | 0.310-2.713 | 8.76E-01 | -3.83E-04 | 9.41E-01 | 0.936653 | 7.17E-01 | 0 |
| Hot drink temperature | 0.796938 | 0.532-1.194 | 2.71E-01 | 1.696608 | 0.586-4.910 | 3.31E-01 | -3.66E-03 | 3.79E-01 | 1.161428 | 3.53E-01 | 0 |
| Cheese intake | 0.919414 | 0.720-1.174 | 5.01E-01 | 0.687175 | 0.387-1.222 | 2.03E-01 | 3.50E-03 | 4.15E-01 | 1.11972 | 2.44E-01 | 0 |
| Water intake | 1.179851 | 0.839-1.659 | 3.41E-01 | 0.912129 | 0.480-1.734 | 7.79E-01 | 1.65E-03 | 6.74E-01 | 0.757916 | 5.65E-02 | 0 |
| Cereal intake | 0.914097 | 0.663-1.260 | 5.83E-01 | 1.476929 | 0.676-3.226 | 3.30E-01 | -5.99E-03 | 1.96E-01 | 0.914057 | 5.70E-01 | 0 |
| Dried fruit intake | 0.907108 | 0.641-1.283 | 5.82E-01 | 0.698515 | 0.269-1.813 | 4.62E-01 | 2.23E-03 | 6.64E-01 | 1.104464 | 5.33E-01 | 0 |
| Alcohol usually taken with meals | 1.025823 | 0.642-1.640 | 9.15E-01 | 0.55023 | 0.131-2.309 | 4.16E-01 | 4.47E-03 | 4.39E-01 | 1.027424 | 8.13E-01 | 0 |
| Average weekly spirits intake | 1.014112 | 0.581-1.769 | 9.61E-01 | 0.336978 | 0.098-1.161 | 9.25E-02 | 1.44E-02 | 9.71E-02 | 0.781295 | 7.00E-02 | 0 |
| Non-oily fish intake | 1.29485 | 0.725-2.313 | 3.83E-01 | 1.402824 | 0.410-4.798 | 5.92E-01 | 5.49E-04 | 9.35E-01 | 0.725878 | 1.22E-01 | 0 |
| Salad / raw vegetable intake | 1.09541 | 0.649-1.850 | 7.33E-01 | 2.051699 | 0.614-6.861 | 2.46E-01 | -5.55E-03 | 3.34E-01 | 0.779296 | 2.05E-01 | 0 |
| Oily fish intake | 1.04475 | 0.765-1.427 | 7.83E-01 | 0.831939 | 0.374-1.852 | 6.53E-01 | 3.77E-03 | 4.53E-01 | 1.007295 | 9.37E-01 | 0 |
| Beef intake | 0.741255 | 0.476-1.154 | 1.85E-01 | 0.70314 | 0.251-1.966 | 5.04E-01 | 8.66E-04 | 8.81E-01 | 0.806429 | 3.47E-02 | 0 |
| Fresh fruit intake | 0.81259 | 0.498-1.327 | 4.07E-01 | 0.367031 | 0.111-1.212 | 1.03E-01 | 7.60E-03 | 1.21E-01 | 1.074943 | 6.47E-01 | 0 |
| Average weekly beer plus cider intake | 1.205731 | 0.747-1.946 | 4.44E-01 | 0.444716 | 0.168-1.178 | 1.06E-01 | 1.04E-02 | 5.69E-02 | 1.220971 | 2.20E-01 | 0 |
| Coffee intake | 1.097207 | 0.748-1.610 | 6.35E-01 | 1.192087 | 0.700-2.031 | 5.19E-01 | -2.08E-03 | 5.37E-01 | 1.076256 | 3.30E-01 | 0 |
| Average weekly red wine intake | 0.781164 | 0.529-1.153 | 2.14E-01 | 0.911301 | 0.399-2.083 | 8.26E-01 | -2.15E-03 | 7.01E-01 | 1.108552 | 3.40E-01 | 0 |
| pork intake | 0.623993 | 0.346-1.125 | 1.17E-01 | 0.22755 | 0.060-0.865 | 3.32E-02 | 1.12E-02 | 7.78E-02 | 0.881878 | 3.72E-01 | 0 |
| Average weekly champagne plus white wine intake | 0.570389 | 0.328-0.993 | 4.73E-02 | 4.268402 | 1.066-17.10 | 4.65E-02 | -2.16E-02 | 1.62E-02 | 1.069542 | 6.06E-01 | 0 |
| Tea intake | 1.114301 | 0.824-1.507 | 4.82E-01 | 0.890852 | 0.55-1.443 | 6.39E-01 | 1.96E-03 | 5.89E-01 | 1.038625 | 7.15E-01 | 0 |
| Processed meat intake | 0.711837 | 0.515-0.984 | 3.97E-02 | 0.821832 | 0.352-1.917 | 6.51E-01 | -2.65E-04 | 9.64E-01 | 0.899008 | 2.77E-01 | 0 |
| Poultry intake | 1.178997 | 0.719-1.933 | 5.14E-01 | 1.332394 | 0.416-4.263 | 6.30E-01 | -2.50E-03 | 7.06E-01 | 0.857357 | 1.33E-01 | 0 |
| Cooked vegetable intake | 1.300268 | 0.780-2.167 | 3.14E-01 | 1.251042 | 0.324-4.837 | 7.46E-01 | -2.45E-04 | 9.71E-01 | 0.956773 | 7.87E-01 | 0 |
| Salt added to food | 1.054815 | 0.818-1.360 | 6.81E-01 | 1.083271 | 0.641-1.83 | 7.65E-01 | -8.42E-05 | 9.80E-01 | 1.472261 | 2.17E-02 | 0 |
| Milk type used: Full cream | 0.417895 | 0.029-6.061 | 5.23E-01 | 0.174327 | 0.001-59.69 | 5.63E-01 | 5.28E-03 | 6.36E-01 | 0.673991 | 6.52E-01 | 0 |
| Milk type used: Semi-skimmed | 0.790786 | 0.078-8.068 | 8.43E-01 | 1.371734 | 0.003-549.4 | 9.20E-01 | -1.41E-03 | 9.46E-01 | 1.124528 | 9.10E-01 | 0 |
| Milk type used: Skimmed | 3.017934 | 0.628-14.51 | 1.68E-01 | 1.068009 | 0.042-26.841 | 9.68E-01 | 3.33E-03 | 7.40E-01 | 1.800141 | 2.82E-01 | 0 |
| Milk type used: Soya | 2.746501 | 0.065-115.6 | 5.96E-01 | 0.188654 | 0.0001-242.2 | 6.52E-01 | 7.44E-03 | 5.01E-01 | 1.922119 | 6.28E-01 | 0 |
| Milk type used: Other type of milk | 2.643467 | 0.0005-14316 | 8.25E-01 | 0.032162 | 0.0000001-10032 | 6.05E-01 | 1.49E-02 | 2.91E-01 | 15.22303 | 3.83E-01 | 0 |
| Milk type used: Never/rarely have milk | 0.183034 | 0.003-12.04 | 4.27E-01 | 0.020021 | 0.000005-81.23 | 3.68E-01 | 7.24E-03 | 5.55E-01 | 0.211032 | 3.38E-01 | 0 |
| Bread type: White | 1.004331 | 0.504-2.003 | 9.90E-01 | 0.204573 | 0.025-1.677 | 1.42E-01 | 1.05E-02 | 1.32E-01 | 1.001447 | 9.95E-01 | 0 |
| Bread type: Brown | 1.514007 | 0.15-15.305 | 7.25E-01 | 0.765418 | 0.006-105.38 | 9.17E-01 | 9.18E-04 | 9.42E-01 | 0.910258 | 8.84E-01 | 0 |
| Bread type: Wholemeal or wholegrain | 0.729545 | 0.38-1.402 | 3.44E-01 | 3.361966 | 0.365-30.94 | 2.87E-01 | -1.02E-02 | 1.99E-01 | 0.802694 | 2.81E-01 | 0 |
| Bread type: Other type of bread | 5.49505 | 0.174-173.9 | 3.34E-01 | 0.035046 | 0.00008-15.33 | 2.95E-01 | 2.03E-02 | 1.02E-01 | 4.797338 | 2.14E-01 | 0 |
| Cereal type: Bran cereal (e.g. All Bran, Branflakes) | 0.494473 | 0.045-5.419 | 5.64E-01 | 9.721172 | 0.244-386.5 | 2.57E-01 | -1.47E-02 | 2.71E-01 | 1.378613 | 7.01E-01 | 0 |
| Cereal type: Biscuit cereal (e.g. Weetabix) | 1.62747 | 0.358-7.400 | 5.29E-01 | 0.731057 | 0.045-11.95 | 8.28E-01 | 3.37E-03 | 7.35E-01 | 1.144309 | 7.85E-01 | 0 |
| Cereal type: Oat cereal (e.g. Ready Brek, porridge) | 0.648058 | 0.136-3.089 | 5.86E-01 | 0.669928 | 0.044-10.26 | 7.76E-01 | 1.44E-03 | 8.93E-01 | 0.798149 | 6.72E-01 | 0 |
| Cereal type: Muesli | 0.678107 | 0.258-1.783 | 4.31E-01 | 1.179902 | 0.143-9.712 | 8.78E-01 | -1.87E-03 | 7.90E-01 | 0.898855 | 7.49E-01 | 0 |
| Cereal type: Other (e.g. Cornflakes, Frosties) | 1.261322 | 0.443-3.593 | 6.64E-01 | 1.740523 | 0.155-19.60 | 6.56E-01 | -4.98E-03 | 5.42E-01 | 0.843992 | 6.49E-01 | 0 |
| Coffee type: Decaffeinated coffee (any type) | 1.394718 | 0.253-7.694 | 7.03E-01 | 3.578224 | 0.168-76.41 | 4.27E-01 | -4.86E-03 | 7.19E-01 | 2.116888 | 2.29E-01 | 0 |
| Coffee type: Instant coffee | 0.91385 | 0.345-2.422 | 8.56E-01 | 0.746936 | 0.072-7.723 | 8.08E-01 | 2.12E-03 | 8.29E-01 | 0.957115 | 8.92E-01 | 0 |
| Coffee type: Ground coffee (include espresso, filter etc) | 0.722 | 0.363-1.435 | 3.52E-01 | 2.546996 | 0.482-13.464 | 2.74E-01 | -8.34E-03 | 1.68E-01 | 0.820874 | 4.17E-01 | 0 |
| Coffee type: Other type of coffee | 0.658027 | 0.0002-2028 | 9.19E-01 | 2.353221 | 7.9E-06-7.0E+05 | 9.01E-01 | -2.52E-03 | 9.11E-01 | NN | 8.84E-01 | 0 |

**Table S20.** Causality of genetically determined 45 dietary habits on OC(HGS) in sensitivity analysis.

| **Exposure** | **Weighted median** | | | **MR Egger** | | | | | **MR Presso** | | |
| --- | --- | --- | --- | --- | --- | --- | --- | --- | --- | --- | --- |
|  | **OR** | **95%CI** | **Pval** | **OR** | **95%CI** | **Pval** | **Intercept** | **Intercept**  **Pval** | **OR** | **Pval** | **N outliers** |
| Age when last ate meat | 1.062915 | 0.791-1.428 | 6.86E-01 | 0.882227 | 0.436-1.785 | 7.36E-01 | 8.93E-03 | 7.51E-01 | 0.98702 | 8.95E-01 | 0 |
| Average weekly fortified wine intake | 1.092614 | 0.201-5.925 | 9.18E-01 | 0.492349 | 0.020-12.24 | 6.70E-01 | 3.15E-03 | 7.89E-01 | 0.740801 | 5.11E-01 | 0 |
| Bread intake | 1.369959 | 0.879-2.136 | 1.65E-01 | 1.077808 | 0.390-2.979 | 8.85E-01 | 3.76E-04 | 9.56E-01 | 0.905901 | 2.45E-01 | 0 |
| lamb/mutton intake | 0.803462 | 0.471-1.372 | 4.23E-01 | 1.105477 | 0.305-4.002 | 8.79E-01 | -2.91E-03 | 6.38E-01 | 0.910349 | 5.95E-01 | 0 |
| Hot drink temperature | 0.816077 | 0.489-1.361 | 4.36E-01 | 0.679105 | 0.185-2.489 | 5.60E-01 | 3.62E-03 | 4.77E-01 | 0.902243 | 5.62E-01 | 0 |
| Cheese intake | 0.770484 | 0.576-1.030 | 7.83E-02 | 0.510189 | 0.252-1.034 | 6.35E-02 | 8.75E-03 | 9.88E-02 | 1.068735 | 5.55E-01 | 0 |
| Water intake | 0.979538 | 0.65-1.476 | 9.21E-01 | 0.916063 | 0.426-1.969 | 8.23E-01 | -8.78E-06 | 9.99E-01 | 0.739616 | 4.95E-02 | 0 |
| Cereal intake | 0.795725 | 0.544-1.163 | 2.38E-01 | 1.246807 | 0.493-3.152 | 6.42E-01 | -5.08E-03 | 3.56E-01 | 0.808377 | 2.55E-01 | 0 |
| Dried fruit intake | 0.83805 | 0.547-1.283 | 4.16E-01 | 0.715557 | 0.229-2.232 | 5.65E-01 | 4.53E-04 | 9.41E-01 | 1.13179 | 5.05E-01 | 0 |
| Alcohol usually taken with meals | 0.788506 | 0.452-1.375 | 4.02E-01 | 0.54776 | 0.091-3.307 | 5.13E-01 | 3.88E-03 | 5.90E-01 | 0.975144 | 8.45E-01 | 0 |
| Average weekly spirits intake | 0.848587 | 0.460-1.564 | 5.99E-01 | 0.351652 | 0.077-1.611 | 1.86E-01 | 1.33E-02 | 2.07E-01 | 0.752483 | 6.77E-02 | 0 |
| Non-oily fish intake | 2.392047 | 1.186-4.824 | 1.48E-02 | 2.263818 | 0.507-10.10 | 2.89E-01 | -3.69E-03 | 6.54E-01 | 0.672728 | 7.91E-02 | 0 |
| Salad / raw vegetable intake | 0.961887 | 0.526-1.759 | 9.00E-01 | 0.703241 | 0.172-2.871 | 6.25E-01 | 2.42E-03 | 7.16E-01 | 0.799743 | 2.99E-01 | 0 |
| Oily fish intake | 1.243357 | 0.873-1.771 | 2.27E-01 | 0.795189 | 0.302-2.094 | 6.43E-01 | 3.76E-03 | 5.37E-01 | 0.950667 | 6.24E-01 | 0 |
| Beef intake | 0.787457 | 0.457-1.356 | 3.89E-01 | 0.718575 | 0.210-2.461 | 6.00E-01 | 3.35E-04 | 9.62E-01 | 0.883253 | 3.15E-01 | 0 |
| Fresh fruit intake | 0.675787 | 0.357-1.278 | 2.28E-01 | 0.642386 | 0.154-2.676 | 5.44E-01 | 1.92E-03 | 7.42E-01 | 1.194907 | 2.79E-01 | 0 |
| Average weekly beer plus cider intake | 1.102372 | 0.634-1.918 | 7.30E-01 | 0.342961 | 0.105-1.122 | 8.04E-02 | 1.38E-02 | 4.05E-02 | 1.431055 | 4.77E-02 | 0 |
| Coffee intake | 0.939228 | 0.593-1.487 | 7.89E-01 | 0.968088 | 0.511-1.833 | 9.21E-01 | 1.02E-04 | 9.80E-01 | 1.102233 | 2.85E-01 | 0 |
| Average weekly red wine intake | 0.813766 | 0.522-1.267 | 3.62E-01 | 0.361787 | 0.142-0.924 | 3.63E-02 | 1.03E-02 | 1.09E-01 | 1.107815 | 4.31E-01 | 0 |
| pork intake | 0.662395 | 0.327-1.34 | 2.52E-01 | 0.208799 | 0.042-1.028 | 5.81E-02 | 1.13E-02 | 1.35E-01 | 0.822443 | 2.46E-01 | 0 |
| Average weekly champagne plus white wine intake | 0.830239 | 0.437-1.577 | 5.70E-01 | 1.635597 | 0.329-8.122 | 5.50E-01 | -9.13E-03 | 3.66E-01 | 1.070778 | 6.68E-01 | 0 |
| Tea intake | 1.094394 | 0.765-1.566 | 6.22E-01 | 0.935879 | 0.524-1.67 | 8.23E-01 | 2.53E-04 | 9.54E-01 | 0.91543 | 4.74E-01 | 0 |
| Processed meat intake | 0.833659 | 0.556-1.250 | 3.78E-01 | 0.821052 | 0.298-2.261 | 7.04E-01 | 1.02E-03 | 8.84E-01 | 0.819433 | 8.83E-02 | 0 |
| Poultry intake | 1.158003 | 0.649-2.065 | 6.19E-01 | 1.529337 | 0.369-6.347 | 5.60E-01 | -2.89E-03 | 7.23E-01 | 0.746056 | 1.63E-02 | 0 |
| Cooked vegetable intake | 1.513494 | 0.836-2.741 | 1.71E-01 | 0.467853 | 0.096-2.287 | 3.51E-01 | 1.13E-02 | 1.56E-01 | 0.888328 | 5.23E-01 | 0 |
| Salt added to food | 0.934313 | 0.685-1.275 | 6.68E-01 | 0.611825 | 0.326-1.149 | 1.28E-01 | 7.63E-03 | 5.49E-02 | 1.63548 | 3.13E-02 | 0 |
| Milk type used: Full cream | 0.414495 | 0.020-8.514 | 5.68E-01 | 0.963342 | 0.001-846.31 | 9.91E-01 | 2.33E-03 | 8.57E-01 | 1.743872 | 5.18E-01 | 0 |
| Milk type used: Semi-skimmed | 4.592135 | 0.376-56.074 | 2.32E-01 | 0.921905 | 0.003-293.6 | 9.78E-01 | 3.03E-03 | 8.78E-01 | 1.420988 | 7.27E-01 | 0 |
| Milk type used: Skimmed | 3.403182 | 0.582-19.89 | 1.74E-01 | 1.045281 | 0.022-49.04 | 9.82E-01 | 4.55E-04 | 9.70E-01 | 1.122873 | 8.48E-01 | 0 |
| Milk type used: Soya | 0.993799 | 0.007-133.5 | 9.98E-01 | 0.544504 | 6.6E-05-4459 | 8.96E-01 | 3.99E-03 | 7.80E-01 | 1.81147 | 7.30E-01 | 0 |
| Milk type used: Other type of milk | 47.36449 | 0.002-1.0E+06 | 4.49E-01 | 131.4192 | 3.9E-05-4.5+08 | 5.38E-01 | 5.52E-04 | 9.73E-01 | 165.1799 | 1.50E-01 | 0 |
| Milk type used: Never/rarely have milk | 0.290429 | 0.001-56.644 | 6.46E-01 | 0.003039 | 2.1E-07-43.06 | 2.49E-01 | 1.57E-02 | 2.72E-01 | 0.501576 | 7.16E-01 | 0 |
| Bread type: White | 0.970471 | 0.444-2.119 | 9.40E-01 | 0.291664 | 0.023-3.671 | 3.43E-01 | 8.76E-03 | 2.96E-01 | 1.093495 | 7.19E-01 | 0 |
| Bread type: Brown | 1.075354 | 0.074-15.582 | 9.58E-01 | 0.541804 | 0.002-189.9 | 8.40E-01 | 9.17E-03 | 5.45E-01 | 3.0582 | 1.78E-01 | 0 |
| Bread type: Wholemeal or wholegrain | 0.638828 | 0.285-1.431 | 2.76E-01 | 5.1554 | 0.363-73.28 | 2.29E-01 | -1.57E-02 | 1.01E-01 | 0.575071 | 1.94E-02 | 0 |
| Bread type: Other type of bread | 11.1666 | 0.171-731.3 | 2.58E-01 | 2.500204 | 0.002-3402 | 8.06E-01 | 1.08E-02 | 4.50E-01 | 33.84671 | 2.31E-02 | 0 |
| Cereal type: Bran cereal (e.g. All Bran, Branflakes) | 1.110652 | 0.064-19.373 | 9.43E-01 | 1.468977 | 0.018-118 | 8.67E-01 | -3.34E-03 | 8.27E-01 | 0.940284 | 9.48E-01 | 0 |
| Cereal type: Biscuit cereal (e.g. Weetabix) | 1.501891 | 0.247-9.135 | 6.59E-01 | 1.424923 | 0.052-38.7 | 8.35E-01 | -1.88E-03 | 8.74E-01 | 1.111372 | 8.46E-01 | 0 |
| Cereal type: Oat cereal (e.g. Ready Brek, porridge) | 1.297546 | 0.215-7.834 | 7.76E-01 | 0.462251 | 0.018-11.79 | 6.46E-01 | 2.69E-03 | 8.32E-01 | 0.640679 | 4.44E-01 | 0 |
| Cereal type: Muesli | 1.173639 | 0.357-3.863 | 7.92E-01 | 0.93208 | 0.076-11.37 | 9.56E-01 | 8.55E-04 | 9.18E-01 | 1.055606 | 8.87E-01 | 0 |
| Cereal type: Other (e.g. Cornflakes, Frosties) | 0.647987 | 0.179-2.344 | 5.08E-01 | 3.154404 | 0.14-70.975 | 4.73E-01 | -1.14E-02 | 2.72E-01 | 0.58182 | 2.51E-01 | 0 |
| Coffee type: Decaffeinated coffee (any type) | 3.058647 | 0.429-21.79 | 2.64E-01 | 4.119925 | 0.118-143.6 | 4.46E-01 | -3.68E-03 | 8.17E-01 | 2.784759 | 1.29E-01 | 0 |
| Coffee type: Instant coffee | 0.68613 | 0.212-2.217 | 5.29E-01 | 0.708596 | 0.044-11.39 | 8.09E-01 | 4.00E-03 | 7.32E-01 | 1.130169 | 7.61E-01 | 0 |
| Coffee type: Ground coffee (include espresso, filter etc) | 0.730957 | 0.323-1.654 | 4.52E-01 | 3.14369 | 0.442-22.342 | 2.55E-01 | -1.17E-02 | 9.99E-02 | 0.637948 | 1.10E-01 | 0 |
| Coffee type: Other type of coffee | 22.93516 | 0.002-2.3E+05 | 5.05E-01 | 1290.037 | 0.0004-3.7E+09 | 3.99E-01 | -1.72E-02 | 5.33E-01 | 16.08823 | 2.06E-01 | 0 |

**Table S21.** Causality of genetically determined 45 dietary habits on OC(LGS) in sensitivity analysis.

| **Exposure** | **Weighted median** | | | **MR Egger** | | | | | **MR Presso** | | |
| --- | --- | --- | --- | --- | --- | --- | --- | --- | --- | --- | --- |
|  | **OR** | **95%CI** | **Pval** | **OR** | **95%CI** | **Pval** | **Intercept** | **Intercept**  **Pval** | **OR** | **Pval** | **N outliers** |
| Age when last ate meat | 1.333986 | 0.561-3.174 | 0.514675 | 0.164996 | 0.020-1.337 | 1.26E-01 | 1.68E-01 | 6.95E-02 | 1.340571 | 3.34E-01 | 0 |
| Average weekly fortified wine intake | 0.021091 | 4.1E-05-10.84 | 0.225639 | 4.35E-07 | 1.8E-12-0.105 | 3.51E-02 | 9.32E-02 | 4.68E-02 | 0.161206 | 4.65E-01 | 0 |
| Bread intake | 1.963243 | 0.534-7.219 | 0.309902 | 1.458522 | 0.064-33.48 | 8.14E-01 | 1.08E-03 | 9.60E-01 | 0.648026 | 7.82E-02 | 0 |
| lamb/mutton intake | 0.477253 | 0.087-2.633 | 0.395928 | 0.141427 | 0.003-6.434 | 3.17E-01 | 1.54E-02 | 4.05E-01 | 1.051085 | 9.35E-01 | 0 |
| Hot drink temperature | 0.733842 | 0.164-3.293 | 0.686199 | 1.82346 | 0.032-102.9 | 7.71E-01 | -6.33E-03 | 6.87E-01 | 2.238712 | 1.43E-01 | 0 |
| Cheese intake | 0.686817 | 0.29-1.628 | 0.393698 | 1.612327 | 0.186-13.997 | 6.65E-01 | -1.38E-02 | 3.90E-01 | 0.928753 | 8.01E-01 | 0 |
| Water intake | 1.505473 | 0.386-5.876 | 0.555955 | 0.975962 | 0.089-10.757 | 9.84E-01 | -1.30E-04 | 9.93E-01 | 0.916779 | 8.64E-01 | 0 |
| Cereal intake | 1.101956 | 0.334-3.632 | 0.873226 | 1.160459 | 0.069-19.604 | 9.18E-01 | -2.72E-04 | 9.87E-01 | 0.785215 | 6.58E-01 | 0 |
| Dried fruit intake | 1.327764 | 0.344-5.132 | 0.681078 | 0.878361 | 0.028-27.085 | 9.41E-01 | 8.65E-03 | 6.41E-01 | 1.442268 | 4.47E-01 | 0 |
| Alcohol usually taken with meals | 0.500383 | 0.098-2.554 | 0.405159 | 5.500936 | 0.029-10401 | 5.25E-01 | -1.68E-02 | 4.26E-01 | 1.158504 | 6.94E-01 | 0 |
| Average weekly spirits intake | 1.085915 | 0.153-7.708 | 0.934305 | 1.66779 | 0.014-191.885 | 8.34E-01 | -6.48E-03 | 8.43E-01 | 0.679056 | 3.51E-01 | 0 |
| Non-oily fish intake | 0.713608 | 0.079-6.436 | 0.763644 | 1.022228 | 0.009-113.8 | 9.93E-01 | 2.83E-03 | 9.12E-01 | 0.888193 | 8.35E-01 | 0 |
| Salad / raw vegetable intake | 4.218182 | 0.612-29.10 | 0.144045 | 1.062174 | 0.013-84.59 | 9.79E-01 | 7.37E-03 | 7.26E-01 | 1.664041 | 4.53E-01 | 0 |
| Oily fish intake | 1.207153 | 0.445-3.276 | 0.711645 | 1.267392 | 0.079-20.23 | 8.67E-01 | -4.04E-03 | 8.20E-01 | 0.93869 | 8.09E-01 | 0 |
| Beef intake | 1.3463 | 0.25-7.252 | 0.729263 | 5.687372 | 0.132-245.2 | 3.68E-01 | -2.13E-02 | 3.20E-01 | 0.91555 | 7.90E-01 | 0 |
| Fresh fruit intake | 0.374362 | 0.052-2.684 | 0.328272 | 0.193001 | 0.003-13.37 | 4.48E-01 | 1.18E-02 | 4.99E-01 | 0.711262 | 4.76E-01 | 0 |
| Average weekly beer plus cider intake | 1.177814 | 0.246-5.637 | 0.837668 | 3.063702 | 0.094-100.2 | 5.31E-01 | -8.61E-03 | 6.57E-01 | 0.736383 | 5.63E-01 | 0 |
| Coffee intake | 0.718613 | 0.157-3.294 | 0.670592 | 1.13599 | 0.163-7.902 | 8.98E-01 | 2.78E-04 | 9.82E-01 | 1.056765 | 8.17E-01 | 0 |
| Average weekly red wine intake | 0.794097 | 0.201-3.139 | 0.742304 | 0.091364 | 0.005-1.737 | 1.15E-01 | 2.80E-02 | 1.62E-01 | 1.576578 | 1.98E-01 | 0 |
| pork intake | 0.83044 | 0.09-7.679 | 0.869951 | 6.138209 | 0.043-876.6 | 4.76E-01 | -1.88E-02 | 4.24E-01 | 0.667783 | 4.10E-01 | 0 |
| Average weekly champagne plus white wine intake | 1.440205 | 0.191-10.85 | 0.723333 | 0.355301 | 0.002-77.75 | 7.09E-01 | 1.99E-02 | 5.63E-01 | 0.817207 | 6.32E-01 | 0 |
| Tea intake | 0.726529 | 0.233-2.262 | 0.581409 | 0.492456 | 0.085-2.843 | 4.30E-01 | 1.03E-02 | 4.35E-01 | 0.96607 | 9.17E-01 | 0 |
| Processed meat intake | 1.114517 | 0.327-3.796 | 0.862335 | 1.091906 | 0.047-25.23 | 9.56E-01 | -2.44E-03 | 9.09E-01 | 1.134564 | 6.86E-01 | 0 |
| Poultry intake | 0.512499 | 0.087-3.032 | 0.461084 | 1.187754 | 0.018-78.01 | 9.36E-01 | -6.11E-03 | 8.02E-01 | 1.930449 | 1.07E-01 | 0 |
| Cooked vegetable intake | 0.616734 | 0.098-3.882 | 0.606589 | 0.614087 | 0.004-95.39 | 8.50E-01 | 1.82E-03 | 9.42E-01 | 0.687846 | 4.48E-01 | 0 |
| Salt added to food | 0.921353 | 0.379-2.238 | 0.856453 | 1.267901 | 0.195-8.238 | 8.04E-01 | -2.39E-03 | 8.41E-01 | 1.314496 | 6.20E-01 | 0 |
| Milk type used: Full cream | 0.408194 | 3.2E-05-5230.3 | 0.852697 | 5.29E-08 | 6.5E-18-428.7 | 1.64E-01 | 6.28E-02 | 1.48E-01 | 0.897258 | 9.72E-01 | 0 |
| Milk type used: Semi-skimmed | 141.3072 | 0.102-194841 | 0.179484 | 4.406313 | 2.9E-07-6.8E+07 | 8.65E-01 | 3.08E-02 | 5.98E-01 | 337.5506 | 1.63E-04 | 0 |
| Milk type used: Skimmed | 8.721372 | 0.039-1945 | 0.432425 | 1.376345 | 3.5E-06-545601 | 9.62E-01 | 6.27E-03 | 8.73E-01 | 3.793285 | 3.55E-01 | 0 |
| Milk type used: Soya | 14.90978 | 1.8E-05-1.2E+07 | 0.697592 | 356912.2 | 1.1E-07-1.2E+18 | 3.95E-01 | -2.88E-02 | 5.05E-01 | 30.15233 | 3.40E-01 | 0 |
| Milk type used: Other type of milk | 0.14466 | 7.0E-16-3.0E+13 | 0.908483 | 0.362829 | 8.3E-23-1.6E+21 | 9.69E-01 | -7.49E-03 | 8.85E-01 | 0.013743 | 6.30E-01 | 0 |
| Milk type used: Never/rarely have milk | 0.001042 | 5.0E-10-2192.6 | 0.355278 | 7.586455 | 2.5E-12-2.3E+13 | 8.92E-01 | -2.82E-02 | 5.19E-01 | 0.001 | 1.22E-01 | 0 |
| Bread type: White | 1.260531 | 0.111-14.27 | 0.851656 | 2.765053 | 0.001-5241.17 | 7.92E-01 | -7.75E-04 | 9.75E-01 | 2.458623 | 2.02E-01 | 0 |
| Bread type: Brown | 0.61704 | 0.0001-3137.6 | 0.911704 | 5396.085 | 1.2E-05-2.5E+12 | 4.11E-01 | -4.80E-02 | 3.45E-01 | 0.443492 | 7.52E-01 | 0 |
| Bread type: Wholemeal or wholegrain | 0.831594 | 0.08-8.641 | 0.87729 | 340.5649 | 0.102-1132849 | 1.63E-01 | -4.63E-02 | 1.13E-01 | 0.523564 | 3.61E-01 | 0 |
| Bread type: Other type of bread | 0.482586 | 1.0E-06-2.3E+05 | 0.91298 | 137.3775 | 8.4E-09-2.2E+12 | 6.88E-01 | -1.40E-02 | 7.57E-01 | 4.25865 | 7.06E-01 | 0 |
| Cereal type: Bran cereal (e.g. All Bran, Branflakes) | 0.020407 | 1.8E-06-226.07 | 0.412728 | 89501.3 | 0.039-2.1E+11 | 1.71E-01 | -9.53E-02 | 9.35E-02 | 0.25351 | 6.84E-01 | 0 |
| Cereal type: Biscuit cereal (e.g. Weetabix) | 8.268811 | 0.033-2086.3 | 0.454072 | 8.302234 | 0.0001-4.1E+05 | 7.05E-01 | 2.03E-03 | 9.57E-01 | 10.93461 | 5.79E-02 | 0 |
| Cereal type: Oat cereal (e.g. Ready Brek, porridge) | 0.02419 | 0.0001-4.387 | 0.160696 | 0.594143 | 2.56E-05-13595.6 | 9.20E-01 | -2.07E-02 | 6.14E-01 | 0.053665 | 1.97E-02 | 0 |
| Cereal type: Muesli | 0.735426 | 0.02-26.715 | 0.866852 | 0.278502 | 0.0001-746.4 | 7.52E-01 | 1.03E-02 | 6.96E-01 | 1.253105 | 8.38E-01 | 0 |
| Cereal type: Other (e.g. Cornflakes, Frosties) | 0.82129 | 0.019-34.853 | 0.917998 | 42.75953 | 0.005-350346 | 4.18E-01 | -2.72E-02 | 3.70E-01 | 0.796819 | 8.56E-01 | 0 |
| Coffee type: Decaffeinated coffee (any type) | 9.337531 | 0.017-5080.98 | 0.486979 | 2335.344 | 0.049-1.1E+08 | 1.80E-01 | -4.21E-02 | 4.09E-01 | 33.4956 | 7.16E-02 | 0 |
| Coffee type: Instant coffee | 3.725447 | 0.114-122 | 0.459885 | 3.064282 | 0.0003-27992 | 8.11E-01 | -3.09E-03 | 9.35E-01 | 2.120396 | 4.36E-01 | 0 |
| Coffee type: Ground coffee (include espresso, filter etc) | 0.300084 | 0.027-3.323 | 0.326495 | 0.217921 | 0.001-87.17 | 6.19E-01 | 4.56E-03 | 8.33E-01 | 0.405727 | 2.18E-01 | 0 |
| Coffee type: Other type of coffee | 13.95992 | 2.6E-12-7.4E+13 | 0.860052 | 39.84271 | 2.4E-19-6.5E+21 | 8.84E-01 | -2.32E-02 | 7.77E-01 | 0.088903 | 7.16E-01 | 0 |

**Table S22.** Causality of genetically determined 45 dietary habits on OC(ED) in sensitivity analysis.

| **Exposure** | **Weighted median** | | | **MR Egger** | | | | | **MR Presso** | | |
| --- | --- | --- | --- | --- | --- | --- | --- | --- | --- | --- | --- |
|  | **OR** | **95%CI** | **Pval** | **OR** | **95%CI** | **Pval** | **Intercept** | **Intercept**  **Pval** | **OR** | **Pval** | **N outliers** |
| Age when last ate meat | 0.929675 | 0.550-1.572 | 7.85E-01 | 0.744198 | 0.207-2.675 | 6.62E-01 | 1.73E-02 | 7.35E-01 | 0.924937 | 6.54E-01 | 0 |
| Average weekly fortified wine intake | 1.113057 | 0.049-25.48 | 9.47E-01 | 1.141204 | 0.003-496.5 | 9.66E-01 | -3.42E-03 | 8.76E-01 | 0.724088 | 7.57E-01 | 0 |
| Bread intake | 1.214307 | 0.555-2.654 | 6.27E-01 | 2.447656 | 0.319-18.79 | 3.92E-01 | -1.45E-02 | 2.86E-01 | 1.058858 | 6.93E-01 | 0 |
| lamb/mutton intake | 1.104276 | 0.408-2.987 | 8.45E-01 | 0.619303 | 0.062-6.186 | 6.84E-01 | 6.65E-03 | 5.52E-01 | 1.248888 | 5.20E-01 | 0 |
| Hot drink temperature | 1.567642 | 0.612-4.017 | 3.49E-01 | 3.702998 | 0.333-41.24 | 2.88E-01 | -8.20E-03 | 3.86E-01 | 1.023073 | 9.42E-01 | 0 |
| Cheese intake | 1.076395 | 0.631-1.835 | 7.87E-01 | 2.035319 | 0.565-7.329 | 2.79E-01 | -1.01E-02 | 2.98E-01 | 0.989459 | 9.58E-01 | 0 |
| Water intake | 0.524469 | 0.240-1.145 | 1.05E-01 | 0.354778 | 0.080-1.577 | 1.76E-01 | 8.84E-03 | 3.34E-01 | 0.408608 | 2.63E-03 | 0 |
| Cereal intake | 1.390997 | 0.701-2.760 | 3.45E-01 | 1.597331 | 0.301-8.490 | 5.83E-01 | -4.96E-03 | 6.18E-01 | 0.803841 | 5.32E-01 | 0 |
| Dried fruit intake | 1.016598 | 0.466-2.216 | 9.67E-01 | 0.354562 | 0.043-2.929 | 3.38E-01 | 1.38E-02 | 2.30E-01 | 0.773967 | 3.69E-01 | 0 |
| Alcohol usually taken with meals | 1.625918 | 0.566-4.675 | 3.67E-01 | 0.926942 | 0.040-21.23 | 9.62E-01 | 2.06E-03 | 8.70E-01 | 1.3502 | 1.88E-01 | 0 |
| Average weekly spirits intake | 1.113807 | 0.326-3.804 | 8.63E-01 | 0.845195 | 0.043-16.44 | 9.12E-01 | 5.54E-03 | 7.89E-01 | 0.989781 | 9.68E-01 | 0 |
| Non-oily fish intake | 1.228973 | 0.343-4.397 | 7.51E-01 | 0.533937 | 0.034-8.289 | 6.56E-01 | 8.68E-03 | 5.69E-01 | 0.923902 | 8.46E-01 | 0 |
| Salad / raw vegetable intake | 1.299453 | 0.419-4.027 | 6.50E-01 | 0.720355 | 0.055-9.519 | 8.04E-01 | 3.42E-03 | 7.80E-01 | 0.462321 | 7.13E-02 | 0 |
| Oily fish intake | 1.050076 | 0.547-2.015 | 8.83E-01 | 0.917813 | 0.168-5.003 | 9.21E-01 | 9.72E-04 | 9.28E-01 | 1.288742 | 1.18E-01 | 0 |
| Beef intake | 0.243233 | 0.092-0.642 | 4.32E-03 | 0.931338 | 0.093-9.340 | 9.52E-01 | -9.49E-03 | 4.64E-01 | 1.090949 | 6.74E-01 | 0 |
| Fresh fruit intake | 0.662789 | 0.197-2.235 | 5.07E-01 | 0.831217 | 0.063-10.99 | 8.89E-01 | -2.81E-04 | 9.79E-01 | 1.232638 | 5.27E-01 | 0 |
| Average weekly beer plus cider intake | 0.924503 | 0.360-2.372 | 8.70E-01 | 0.971782 | 0.108-8.756 | 9.80E-01 | -2.56E-03 | 8.32E-01 | 1.056448 | 8.70E-01 | 0 |
| Coffee intake | 2.735099 | 1.088-6.876 | 3.24E-02 | 3.284927 | 1.036-10.41 | 4.61E-02 | -1.25E-02 | 8.90E-02 | 1.135352 | 4.20E-01 | 0 |
| Average weekly red wine intake | 0.976178 | 0.419-2.276 | 9.55E-01 | 3.423798 | 0.538-21.77 | 1.96E-01 | -1.73E-02 | 1.70E-01 | 0.83593 | 4.24E-01 | 0 |
| pork intake | 1.299179 | 0.355-4.760 | 6.93E-01 | 0.573615 | 0.03-10.938 | 7.13E-01 | 4.68E-03 | 7.39E-01 | 1.206682 | 5.12E-01 | 0 |
| Average weekly champagne plus white wine intake | 0.417668 | 0.121-1.437 | 1.66E-01 | 1.828735 | 0.099-33.94 | 6.87E-01 | -1.76E-02 | 3.40E-01 | 1.320897 | 2.69E-01 | 0 |
| Tea intake | 2.036445 | 1.020-4.065 | 4.37E-02 | 2.021539 | 0.709-5.763 | 1.90E-01 | -7.26E-03 | 3.62E-01 | 0.712102 | 9.79E-02 | 0 |
| Processed meat intake | 1.090695 | 0.534-2.228 | 8.12E-01 | 0.68046 | 0.110-4.205 | 6.79E-01 | 6.60E-03 | 5.98E-01 | 1.061979 | 7.43E-01 | 0 |
| Poultry intake | 1.399852 | 0.478-4.103 | 5.40E-01 | 1.200347 | 0.092-15.70 | 8.90E-01 | 3.15E-04 | 9.83E-01 | 1.241947 | 3.73E-01 | 0 |
| Cooked vegetable intake | 1.210338 | 0.38-3.851 | 7.46E-01 | 0.177721 | 0.010-3.118 | 2.41E-01 | 1.83E-02 | 2.07E-01 | 1.195342 | 6.14E-01 | 0 |
| Salt added to food | 1.175763 | 0.667-2.074 | 5.76E-01 | 1.305862 | 0.418-4.083 | 6.47E-01 | -1.82E-03 | 8.00E-01 | 1.140062 | 7.39E-01 | 0 |
| Milk type used: Full cream | 0.160935 | 0.0004-61.02 | 5.47E-01 | 1.491261 | 4.6E-06-4.9E+06 | 9.51E-01 | -1.84E-03 | 9.40E-01 | 0.93338 | 9.72E-01 | 0 |
| Milk type used: Semi-skimmed | 1.006721 | 0.011-93.00 | 9.98E-01 | 7.759069 | 0.0002-243891 | 7.07E-01 | -7.82E-03 | 8.28E-01 | 2.553625 | 5.91E-01 | 0 |
| Milk type used: Skimmed | 9.923543 | 0.363-271.7 | 1.74E-01 | 1.596046 | 0.002-1698.082 | 8.96E-01 | 4.66E-03 | 8.29E-01 | 3.316467 | 2.34E-01 | 0 |
| Milk type used: Soya | 0.344925 | 0.0001-1036 | 7.94E-01 | 3.18E-05 | 2.9E-12-353.7 | 2.25E-01 | 2.57E-02 | 3.15E-01 | 0.089184 | 2.33E-01 | 0 |
| Milk type used: Other type of milk | 23.70457 | 1.2E-08-4.6E+10 | 7.72E-01 | 1.668056 | 3.6E-13-7.8E+12 | 9.73E-01 | -3.42E-03 | 9.24E-01 | 0.484115 | 9.19E-01 | 0 |
| Milk type used: Never/rarely have milk | 0.001645 | 3.4E-07-7.898 | 1.38E-01 | 1.60E-06 | 8.5E-14-30.37 | 1.35E-01 | 2.85E-02 | 2.54E-01 | 0.017619 | 1.17E-01 | 0 |
| Bread type: White | 0.137569 | 0.031-0.614 | 9.38E-03 | 0.009639 | 8.6E-05-1.074 | 5.63E-02 | 2.25E-02 | 1.51E-01 | 0.286261 | 1.15E-02 | 0 |
| Bread type: Brown | 0.004731 | 1.7E-05-1.34 | 6.31E-02 | 0.001107 | 8.9E-09-138.4 | 2.76E-01 | 2.05E-02 | 4.82E-01 | 0.063969 | 1.59E-01 | 0 |
| Bread type: Wholemeal or wholegrain | 3.520597 | 0.836-14.82 | 8.61E-02 | 7.281946 | 0.042-1261 | 4.52E-01 | -8.45E-03 | 6.44E-01 | 2.205857 | 9.10E-02 | 0 |
| Bread type: Other type of bread | 0.625125 | 0.0003-1471 | 9.06E-01 | 0.035066 | 4.4E-08-28021 | 6.35E-01 | 1.27E-02 | 6.43E-01 | 0.712291 | 8.83E-01 | 0 |
| Cereal type: Bran cereal (e.g. All Bran, Branflakes) | 27.13103 | 0.198-3715 | 1.88E-01 | 262.454 | 0.079-8.7E+05 | 2.15E-01 | -1.97E-02 | 5.04E-01 | 20.65831 | 3.43E-02 | 0 |
| Cereal type: Biscuit cereal (e.g. Weetabix) | 0.874502 | 0.029-26.147 | 9.38E-01 | 0.035371 | 6.2E-05-20.318 | 3.13E-01 | 2.09E-02 | 3.69E-01 | 0.548901 | 5.82E-01 | 0 |
| Cereal type: Oat cereal (e.g. Ready Brek, porridge) | 0.46591 | 0.017-12.749 | 6.51E-01 | 14.8771 | 0.03-7343 | 4.05E-01 | -2.65E-02 | 2.83E-01 | 0.591376 | 5.72E-01 | 0 |
| Cereal type: Muesli | 1.125106 | 0.127-9.998 | 9.16E-01 | 2.929857 | 0.028-304.9 | 6.52E-01 | -8.97E-03 | 5.69E-01 | 0.813754 | 7.71E-01 | 0 |
| Cereal type: Other (e.g. Cornflakes, Frosties) | 3.626886 | 0.414-31.75 | 2.44E-01 | 2.473016 | 0.01-594.585 | 7.48E-01 | 1.80E-03 | 9.21E-01 | 3.230328 | 7.54E-02 | 0 |
| Coffee type: Decaffeinated coffee (any type) | 1.720139 | 0.049-60.79 | 7.66E-01 | 0.590344 | 0.001-550.8 | 8.82E-01 | 1.37E-02 | 6.70E-01 | 2.376024 | 3.79E-01 | 0 |
| Coffee type: Instant coffee | 0.771792 | 0.092-6.506 | 8.12E-01 | 2.072062 | 0.013-331.4 | 7.80E-01 | -7.71E-03 | 7.20E-01 | 0.848815 | 8.08E-01 | 0 |
| Coffee type: Ground coffee (include espresso, filter etc) | 1.033303 | 0.217-4.911 | 9.67E-01 | 3.601846 | 0.092-141.6 | 4.96E-01 | -1.01E-02 | 4.48E-01 | 0.917063 | 8.73E-01 | 0 |
| Coffee type: Other type of coffee | 67.43508 | 6.8E-08-6.7E+10 | 6.90E-01 | 817031.1 | 8.0E-09-8.3E+19 | 4.69E-01 | -1.91E-02 | 7.28E-01 | 3521.271 | 2.40E-01 | 0 |

**Table S23.** Causality of genetically determined 45 dietary habits on OC(CC) in sensitivity analysis.

| **Exposure** | **Weighted median** | | | **MR Egger** | | | | | **MR Presso** | | |
| --- | --- | --- | --- | --- | --- | --- | --- | --- | --- | --- | --- |
|  | **OR** | **95%CI** | **Pval** | **OR** | **95%CI** | **Pval** | **Intercept** | **Intercept**  **Pval** | **OR** | **Pval** | **N outliers** |
| Age when last ate meat | 1.333329 | 0.604-2.941 | 4.76E-01 | 3.786801 | 0.506-28.34 | 2.27E-01 | -1.18E-01 | 1.61E-01 | 0.847417 | 6.23E-01 | 0 |
| Average weekly fortified wine intake | 1.43912 | 0.016-132.7 | 8.75E-01 | 0.003348 | 8.4E-07-13.41 | 1.94E-01 | 4.52E-02 | 1.51E-01 | 1.139214 | 9.27E-01 | 0 |
| Bread intake | 0.939471 | 0.322-2.737 | 9.09E-01 | 0.361268 | 0.026-5.046 | 4.51E-01 | 1.43E-02 | 4.16E-01 | 0.845231 | 4.02E-01 | 0 |
| lamb/mutton intake | 0.796137 | 0.200-3.165 | 7.46E-01 | 0.247474 | 0.009-6.565 | 4.06E-01 | 1.01E-02 | 5.25E-01 | 0.589762 | 2.89E-01 | 0 |
| Hot drink temperature | 0.533405 | 0.149-1.904 | 3.33E-01 | 0.222803 | 0.007-7.107 | 3.97E-01 | 9.75E-03 | 4.74E-01 | 1.193732 | 7.09E-01 | 0 |
| Cheese intake | 0.99768 | 0.483-2.062 | 9.95E-01 | 0.970176 | 0.150-6.267 | 9.75E-01 | -2.09E-03 | 8.80E-01 | 0.890349 | 6.60E-01 | 0 |
| Water intake | 1.343772 | 0.449-4.018 | 5.97E-01 | 0.737186 | 0.102-5.337 | 7.63E-01 | 7.92E-03 | 5.11E-01 | 0.594001 | 2.26E-01 | 0 |
| Cereal intake | 0.904426 | 0.347-2.356 | 8.37E-01 | 4.334201 | 0.409-45.94 | 2.25E-01 | -2.28E-02 | 1.07E-01 | 0.784708 | 6.01E-01 | 0 |
| Dried fruit intake | 1.438072 | 0.477-4.338 | 5.19E-01 | 0.532199 | 0.028-10.13 | 6.75E-01 | 8.95E-03 | 5.71E-01 | 0.396956 | 2.06E-02 | 0 |
| Alcohol usually taken with meals | 1.483974 | 0.338-6.524 | 6.01E-01 | 4.871569 | 0.064-372.8 | 4.75E-01 | -8.96E-03 | 6.09E-01 | 1.136506 | 6.98E-01 | 0 |
| Average weekly spirits intake | 0.485311 | 0.095-2.468 | 3.84E-01 | 0.264789 | 0.007-10.75 | 4.86E-01 | 1.14E-02 | 6.58E-01 | 1.24441 | 5.32E-01 | 0 |
| Non-oily fish intake | 1.787031 | 0.262-12.19 | 5.53E-01 | 0.880374 | 0.015-50.92 | 9.51E-01 | 5.45E-03 | 8.04E-01 | 0.813454 | 6.94E-01 | 0 |
| Salad / raw vegetable intake | 1.533934 | 0.305-7.714 | 6.04E-01 | 0.592079 | 0.012-28.26 | 7.91E-01 | 6.66E-03 | 7.10E-01 | 0.491886 | 1.46E-01 | 0 |
| Oily fish intake | 0.953162 | 0.388-2.341 | 9.17E-01 | 1.305316 | 0.119-14.37 | 8.28E-01 | -4.91E-03 | 7.47E-01 | 1.259484 | 2.95E-01 | 0 |
| Beef intake | 0.937232 | 0.214-4.103 | 9.31E-01 | 0.149628 | 0.006-3.778 | 2.52E-01 | 1.63E-02 | 3.80E-01 | 0.806627 | 4.21E-01 | 0 |
| Fresh fruit intake | 0.371582 | 0.073-1.902 | 2.35E-01 | 0.116972 | 0.003-4.314 | 2.46E-01 | 1.60E-02 | 2.82E-01 | 0.663557 | 2.79E-01 | 0 |
| Average weekly beer plus cider intake | 0.307633 | 0.079-1.204 | 9.04E-02 | 0.135823 | 0.007-2.748 | 1.97E-01 | 1.23E-02 | 4.63E-01 | 1.192385 | 7.12E-01 | 0 |
| Coffee intake | 1.222089 | 0.367-4.072 | 7.44E-01 | 1.061037 | 0.210-5.355 | 9.43E-01 | 9.86E-04 | 9.25E-01 | 0.956784 | 8.31E-01 | 0 |
| Average weekly red wine intake | 0.737979 | 0.228-2.388 | 6.12E-01 | 1.471195 | 0.125-17.26 | 7.59E-01 | -2.36E-03 | 8.88E-01 | 1.036764 | 9.02E-01 | 0 |
| pork intake | 1.626133 | 0.286-9.242 | 5.83E-01 | 0.253245 | 0.004-17.381 | 5.27E-01 | 1.11E-02 | 5.73E-01 | 0.685551 | 3.46E-01 | 0 |
| Average weekly champagne plus white wine intake | 0.362318 | 0.073-1.808 | 2.16E-01 | 0.581579 | 0.008-40.432 | 8.03E-01 | -2.12E-03 | 9.36E-01 | 0.757257 | 4.08E-01 | 0 |
| Tea intake | 1.300051 | 0.505-3.349 | 5.87E-01 | 1.160175 | 0.269-4.995 | 8.42E-01 | 1.31E-03 | 9.05E-01 | 1.37933 | 2.59E-01 | 0 |
| Processed meat intake | 0.810073 | 0.289-2.268 | 6.88E-01 | 1.253716 | 0.076-20.56 | 8.74E-01 | -6.08E-03 | 7.50E-01 | 0.669381 | 1.24E-01 | 0 |
| Poultry intake | 0.98971 | 0.225-4.345 | 9.89E-01 | 0.862225 | 0.022-34.093 | 9.37E-01 | -3.04E-03 | 8.85E-01 | 1.214821 | 5.38E-01 | 0 |
| Cooked vegetable intake | 0.652402 | 0.131-3.256 | 6.03E-01 | 0.155838 | 0.002-15.143 | 4.29E-01 | 2.04E-02 | 3.71E-01 | 1.611379 | 3.37E-01 | 0 |
| Salt added to food | 1.013826 | 0.470-2.188 | 9.72E-01 | 1.214067 | 0.249-5.908 | 8.10E-01 | -3.13E-03 | 7.56E-01 | 1.437192 | 4.95E-01 | 0 |
| Milk type used: Full cream | 0.024794 | 7.4E-06-83.35 | 3.72E-01 | 1.22401 | 4.4E-08-3.4E+07 | 9.82E-01 | -1.46E-02 | 6.60E-01 | 0.031086 | 1.44E-01 | 0 |
| Milk type used: Semi-skimmed | 0.634702 | 0.002-221.2 | 8.79E-01 | 0.566073 | 1.5E-06-2.1E+05 | 9.32E-01 | 1.06E-02 | 8.16E-01 | 2.436537 | 5.72E-01 | 0 |
| Milk type used: Skimmed | 529.8451 | 5.394-52049 | 7.36E-03 | 89.20673 | 0.003-2.3E+06 | 3.96E-01 | -6.39E-03 | 8.40E-01 | 32.82606 | 4.71E-02 | 0 |
| Milk type used: Soya | 11.18566 | 0.0001-984317 | 6.78E-01 | 0.220015 | 8.2E-11-5.9E+08 | 8.92E-01 | 6.57E-03 | 8.44E-01 | 1.714909 | 8.69E-01 | 0 |
| Milk type used: Other type of milk | 6.924884 | 1.7E-11-2.8E+12 | 8.87E-01 | 54.92729 | 4.0E-16-7.6E+18 | 8.46E-01 | -8.64E-04 | 9.84E-01 | 38.06127 | 6.92E-01 | 0 |
| Milk type used: Never/rarely have milk | 0.51212 | 1.6E-06-1.60E+05 | 9.17E-01 | 1040.452 | 1.2E-08-9.3E+13 | 5.97E-01 | -3.09E-02 | 4.17E-01 | 0.050251 | 3.94E-01 | 0 |
| Bread type: White | 0.271585 | 0.037-1.980 | 1.98E-01 | 0.00382 | 5.1E-06-2.842 | 1.02E-01 | 3.00E-02 | 1.70E-01 | 0.360723 | 9.53E-02 | 0 |
| Bread type: Brown | 0.502659 | 0.0002-895.8 | 8.57E-01 | 0.739756 | 1.3E-08-4.2E+07 | 9.74E-01 | -7.84E-03 | 8.61E-01 | 0.157958 | 3.90E-01 | 0 |
| Bread type: Wholemeal or wholegrain | 1.339651 | 0.170-10.565 | 7.81E-01 | 51.17314 | 0.05-52816 | 2.70E-01 | -2.36E-02 | 3.42E-01 | 1.866814 | 3.38E-01 | 0 |
| Bread type: Other type of bread | 0.11134 | 2.1E-06-5890 | 6.92E-01 | 5.93E-05 | 2.6E-13-1.4E+04 | 3.37E-01 | 5.14E-02 | 1.78E-01 | 20.23047 | 3.96E-01 | 0 |
| Cereal type: Bran cereal (e.g. All Bran, Branflakes) | 0.90294 | 0.0004-1844 | 9.79E-01 | 0.083779 | 4.6E-09-1.5E+06 | 7.80E-01 | 2.27E-02 | 6.68E-01 | 2.995166 | 6.76E-01 | 0 |
| Cereal type: Biscuit cereal (e.g. Weetabix) | 0.455317 | 0.005-42.37 | 7.34E-01 | 0.603676 | 0.0001-3099 | 9.09E-01 | 2.97E-03 | 9.22E-01 | 0.897625 | 9.39E-01 | 0 |
| Cereal type: Oat cereal (e.g. Ready Brek, porridge) | 1.98229 | 0.018-221.9 | 7.76E-01 | 80.98079 | 0.021-309205 | 3.09E-01 | -3.16E-02 | 3.34E-01 | 1.772857 | 7.19E-01 | 0 |
| Cereal type: Muesli | 0.592825 | 0.029-11.98 | 7.33E-01 | 2.008351 | 0.003-1416 | 8.36E-01 | -3.34E-04 | 9.88E-01 | 1.913433 | 4.23E-01 | 0 |
| Cereal type: Other (e.g. Cornflakes, Frosties) | 0.068314 | 0.003-1.753 | 1.05E-01 | 0.056681 | 3.6E-05-89.32 | 4.49E-01 | 4.92E-03 | 8.43E-01 | 0.11546 | 3.52E-02 | 0 |
| Coffee type: Decaffeinated coffee (any type) | 3.519887 | 0.016-777.9 | 6.48E-01 | 0.378596 | 3.1E-05-4573 | 8.42E-01 | 1.23E-02 | 7.65E-01 | 1.439438 | 8.47E-01 | 0 |
| Coffee type: Instant coffee | 3.072131 | 0.099-95.20 | 5.22E-01 | 0.175282 | 3.3E-05-935.2 | 6.94E-01 | 1.70E-02 | 6.42E-01 | 1.245364 | 8.70E-01 | 0 |
| Coffee type: Ground coffee (include espresso, filter etc) | 0.398703 | 0.052-3.075 | 3.78E-01 | 4.077618 | 0.027-612.186 | 5.84E-01 | -6.33E-03 | 7.27E-01 | 1.729252 | 4.13E-01 | 0 |
| Coffee type: Other type of coffee | 3512.834 | 2.0E-08-6.2E+14 | 5.37E-01 | 0.000102 | 2.0E-21-5.3E+12 | 6.64E-01 | 8.15E-02 | 2.74E-01 | 162991.6 | 2.24E-01 | 0 |

**Table S24.** Causality of genetically determined 45 dietary habits on OC(IM) in sensitivity analysis.

| **Exposure** | **Weighted median** | | | **MR Egger** | | | | | **MR Presso** | | |
| --- | --- | --- | --- | --- | --- | --- | --- | --- | --- | --- | --- |
|  | **OR** | **95%CI** | **Pval** | **OR** | **95%CI** | **Pval** | **Intercept** | **Intercept**  **Pval** | **OR** | **Pval** | **N outliers** |
| Age when last ate meat | 0.975991 | 0.479-1.988 | 9.47E-01 | 0.27013 | 0.04-1.842 | 2.14E-01 | 7.48E-02 | 3.41E-01 | 0.690305 | 2.45E-01 | 0 |
| Average weekly fortified wine intake | 0.698549 | 0.01-50.891 | 8.70E-01 | 43.92245 | 0.014-141271 | 3.71E-01 | -3.67E-02 | 2.30E-01 | 0.413321 | 3.49E-01 | 0 |
| Bread intake | 0.845069 | 0.303-2.357 | 7.48E-01 | 1.58977 | 0.113-22.301 | 7.32E-01 | -1.12E-02 | 5.28E-01 | 0.755743 | 1.63E-01 | 0 |
| lamb/mutton intake | 0.458777 | 0.115-1.824 | 2.68E-01 | 0.950096 | 0.038-23.958 | 9.75E-01 | -4.10E-03 | 7.94E-01 | 0.874748 | 7.87E-01 | 0 |
| Hot drink temperature | 1.027686 | 0.289-3.655 | 9.66E-01 | 2.458074 | 0.095-63.453 | 5.88E-01 | -9.24E-03 | 4.72E-01 | 1.877762 | 1.20E-01 | 0 |
| Cheese intake | 0.870558 | 0.443-1.709 | 6.87E-01 | 1.999781 | 0.358-11.157 | 4.31E-01 | -1.50E-02 | 2.48E-01 | 1.244697 | 3.21E-01 | 0 |
| Water intake | 1.699325 | 0.586-4.927 | 3.29E-01 | 0.739328 | 0.104-5.262 | 7.63E-01 | 1.02E-02 | 3.94E-01 | 0.797905 | 5.24E-01 | 0 |
| Cereal intake | 0.726133 | 0.275-1.920 | 5.19E-01 | 0.378942 | 0.034-4.172 | 4.29E-01 | 6.26E-03 | 6.57E-01 | 0.737236 | 4.51E-01 | 0 |
| Dried fruit intake | 0.776892 | 0.269-2.242 | 6.41E-01 | 1.161560 | 0.062-21.853 | 9.20E-01 | -5.52E-03 | 7.28E-01 | 0.658964 | 3.66E-01 | 0 |
| Alcohol usually taken with meals | 1.153166 | 0.292-4.558 | 8.39E-01 | 0.149474 | 0.002-12.222 | 3.99E-01 | 1.01E-02 | 5.71E-01 | 0.717026 | 2.95E-01 | 0 |
| Average weekly spirits intake | 0.766369 | 0.165-3.567 | 7.34E-01 | 0.282230 | 0.004-19.459 | 5.62E-01 | 1.57E-02 | 5.89E-01 | 0.691201 | 2.97E-01 | 0 |
| Non-oily fish intake | 1.503471 | 0.286-7.897 | 6.30E-01 | 5.661300 | 0.139-230.185 | 3.63E-01 | -2.03E-02 | 3.10E-01 | 0.605249 | 3.29E-01 | 0 |
| Salad / raw vegetable intake | 1.337467 | 0.294-6.094 | 7.07E-01 | 2.701380 | 0.072-101.28 | 5.92E-01 | -3.57E-03 | 8.37E-01 | 0.356602 | 2.92E-02 | 0 |
| Oily fish intake | 1.460223 | 0.633-3.369 | 3.75E-01 | 0.441811 | 0.043-4.505 | 4.92E-01 | 1.35E-02 | 3.67E-01 | 0.966844 | 8.91E-01 | 0 |
| Beef intake | 1.100773 | 0.301-4.027 | 8.85E-01 | 1.360099 | 0.05-37.32 | 8.56E-01 | -6.06E-03 | 7.42E-01 | 0.602813 | 6.30E-02 | 0 |
| Fresh fruit intake | 0.757222 | 0.179-3.210 | 7.06E-01 | 0.1982710 | 0.006-6.809 | 3.72E-01 | 1.11E-02 | 4.49E-01 | 1.60173 | 2.20E-01 | 0 |
| Average weekly beer plus cider intake | 0.559784 | 0.143-2.189 | 4.04E-01 | 4.624651828 | 0.24-89.173 | 3.13E-01 | -2.21E-02 | 1.77E-01 | 0.836967 | 6.75E-01 | 0 |
| Coffee intake | 0.655235 | 0.202-2.124 | 4.81E-01 | 1.143625825 | 0.235-5.575 | 8.68E-01 | -6.61E-03 | 5.12E-01 | 1.515364 | 4.27E-02 | 0 |
| Average weekly red wine intake | 0.675419 | 0.218-2.096 | 4.97E-01 | 9.478097959 | 0.76-118.225 | 8.42E-02 | -3.62E-02 | 3.54E-02 | 0.701509 | 2.65E-01 | 0 |
| pork intake | 0.459785 | 0.079-2.679 | 3.88E-01 | 0.671961569 | 0.011-40.805 | 8.50E-01 | -1.00E-03 | 9.58E-01 | 0.629104 | 2.07E-01 | 0 |
| Average weekly champagne plus white wine intake | 0.311737 | 0.063-1.553 | 1.55E-01 | 11.89527502 | 0.213-665.133 | 2.34E-01 | -4.47E-02 | 8.15E-02 | 0.775747 | 4.80E-01 | 0 |
| Tea intake | 1.168901 | 0.448-3.05 | 7.50E-01 | 0.798180652 | 0.185-3.452 | 7.63E-01 | 3.08E-03 | 7.81E-01 | 1.655604 | 5.14E-02 | 0 |
| Processed meat intake | 0.588896 | 0.218-1.592 | 2.97E-01 | 1.671534097 | 0.103-27.19 | 7.19E-01 | -1.39E-02 | 4.60E-01 | 0.63917 | 7.10E-02 | 0 |
| Poultry intake | 1.532723 | 0.34-6.916 | 5.79E-01 | 8.254245344 | 0.251-271.929 | 2.41E-01 | -1.97E-02 | 3.37E-01 | 0.701654 | 2.41E-01 | 0 |
| Cooked vegetable intake | 0.43165 | 0.104-1.787 | 2.46E-01 | 0.773399945 | 0.016-37.684 | 8.97E-01 | 8.16E-04 | 9.67E-01 | 0.519232 | 1.59E-01 | 0 |
| Salt added to food | 1.946607 | 0.904-4.192 | 8.88E-02 | 4.491858582 | 0.926-21.794 | 6.37E-02 | -1.43E-02 | 1.56E-01 | 0.909935 | 8.52E-01 | 0 |
| Milk type used: Full cream | 3.545309 | 0.001-11229.7 | 7.58E-01 | 0.053996562 | 2.4E-09-1.2E+06 | 7.38E-01 | 4.53E-03 | 8.91E-01 | 0.167827 | 5.26E-01 | 0 |
| Milk type used: Semi-skimmed | 0.24932 | 0.0003-209.4 | 6.86E-01 | 8.579191772 | 2.2E-05-3.3E+06 | 7.52E-01 | -1.86E-02 | 6.98E-01 | 0.748858 | 9.00E-01 | 0 |
| Milk type used: Skimmed | 5.77811 | 0.044-764.161 | 4.82E-01 | 29.01523331 | 8.7E-05-9.7E+06 | 6.09E-01 | -1.71E-02 | 6.53E-01 | 1.670152 | 7.42E-01 | 0 |
| Milk type used: Soya | 345.8336 | 0.005-2.4E+07 | 3.04E-01 | 93.60982078 | 9.0E-10-9.7E+12 | 7.30E-01 | -1.38E-03 | 9.70E-01 | 58.9429 | 1.78E-01 | 0 |
| Milk type used: Other type of milk | 0.792244 | 6.3E-12-9.9E+10 | 9.86E-01 | 13.50585926 | 8.0E-18-2.3E+19 | 9.06E-01 | -2.78E-03 | 9.49E-01 | 3.975051 | 8.54E-01 | 0 |
| Milk type used: Never/rarely have milk | 2691.869 | 0.012-6.3E+08 | 2.10E-01 | 5340.54344 | 1.6E-08-1.7E+15 | 5.35E-01 | -1.13E-02 | 7.62E-01 | 108.5538 | 1.76E-01 | 0 |
| Bread type: White | 1.841047 | 0.254-13.355 | 5.46E-01 | 7.589516575 | 0.014-4109.215 | 5.29E-01 | -1.47E-02 | 4.80E-01 | 0.826176 | 7.40E-01 | 0 |
| Bread type: Brown | 0.0807 | 0.00005-108.9 | 4.94E-01 | 0.012851984 | 1.8E-09-9.2E+04 | 5.97E-01 | 1.12E-02 | 7.79E-01 | 0.111046 | 2.70E-01 | 0 |
| Bread type: Wholemeal or wholegrain | 2.677263 | 0.385-18.609 | 3.19E-01 | 2.104547411 | 0.002-2799 | 8.40E-01 | 2.51E-03 | 9.22E-01 | 2.995855 | 7.53E-02 | 0 |
| Bread type: Other type of bread | 0.032755 | 1.2E-06-910.9 | 5.13E-01 | 1.57E-05 | 3.3E-14-7245 | 2.92E-01 | 2.96E-02 | 4.37E-01 | 0.028825 | 2.79E-01 | 0 |
| Cereal type: Bran cereal (e.g. All Bran, Branflakes) | 2.822804 | 0.001-8345 | 7.99E-01 | 1936.580066 | 0.003-1.2E+09 | 3.03E-01 | -5.74E-02 | 2.56E-01 | 1.152203 | 9.67E-01 | 0 |
| Cereal type: Biscuit cereal (e.g. Weetabix) | 1.856935 | 0.018-195.9 | 7.95E-01 | 0.389291555 | 7.4E-05-2042 | 8.31E-01 | 1.40E-02 | 6.46E-01 | 2.499797 | 4.93E-01 | 0 |
| Cereal type: Oat cereal (e.g. Ready Brek, porridge) | 1.545397 | 0.01-230.325 | 8.65E-01 | 0.150144217 | 0.00002-1129 | 6.82E-01 | 1.23E-02 | 7.18E-01 | 0.708673 | 8.31E-01 | 0 |
| Cereal type: Muesli | 5.076524 | 0.287-89.929 | 2.68E-01 | 2.115251658 | 0.004-1150 | 8.17E-01 | 1.43E-03 | 9.46E-01 | 2.599869 | 2.99E-01 | 0 |
| Cereal type: Other (e.g. Cornflakes, Frosties) | 0.474607 | 0.023-9.767 | 6.29E-01 | 0.416005017 | 0.0003-567.3 | 8.13E-01 | 4.85E-03 | 8.41E-01 | 0.843238 | 8.70E-01 | 0 |
| Coffee type: Decaffeinated coffee (any type) | 1.192644 | 0.007-212.4 | 9.47E-01 | 2.162623376 | 0.0002-20686 | 8.71E-01 | 9.66E-03 | 8.11E-01 | 6.122034 | 3.02E-01 | 0 |
| Coffee type: Instant coffee | 0.123247 | 0.007-2.145 | 1.51E-01 | 0.339683796 | 0.0003-372.4 | 7.64E-01 | -3.90E-03 | 8.95E-01 | 0.215603 | 1.06E-01 | 0 |
| Coffee type: Ground coffee (include espresso, filter etc) | 1.798854 | 0.242-13.38 | 5.66E-01 | 6.29884445 | 0.038-1034 | 4.81E-01 | -1.32E-02 | 4.72E-01 | 1.037397 | 9.52E-01 | 0 |
| Coffee type: Other type of coffee | 1.068057 | 2.4E-11-4.8E+10 | 9.96E-01 | 6.49E-07 | 1.2E-23-3.5E+10 | 5.09E-01 | 2.20E-02 | 7.49E-01 | 0.000203 | 2.76E-01 | 0 |

**Table S25.** Causality of genetically determined 45 dietary habits on PC in sensitivity analysis.

| **Exposure** | **Weighted median** | | | **MR Egger** | | | | | **MR Presso** | | |
| --- | --- | --- | --- | --- | --- | --- | --- | --- | --- | --- | --- |
|  | **OR** | **95%CI** | **Pval** | **OR** | **95%CI** | **Pval** | **Intercept** | **Intercept**  **Pval** | **OR** | **Pval** | **N outliers** |
| Age when last ate meat | 0.991493 | 0.852-1.154 | 9.12E-01 | 1.365808 | 0.851-2.193 | 2.26E-01 | -2.10E-02 | 2.62E-01 | 1.038574 | 6.20E-01 | 0 |
| Average weekly fortified wine intake | 1.46798 | 0.597-3.612 | 4.03E-01 | 2.966286 | 0.579-15.19 | 2.08E-01 | -6.39E-03 | 2.92E-01 | 1.295734 | 3.63E-01 | 0 |
| Bread intake | 1.145008 | 0.905-1.449 | 2.59E-01 | 0.98083 | 0.578-1.665 | 9.43E-01 | 1.49E-03 | 6.72E-01 | 0.969373 | 5.73E-01 | 0 |
| lamb/mutton intake | 1.040148 | 0.766-1.413 | 8.01E-01 | 1.658508 | 0.848-3.245 | 1.42E-01 | -4.11E-03 | 2.09E-01 | 0.956134 | 7.19E-01 | 0 |
| Hot drink temperature | 0.92488 | 0.707-1.21 | 5.69E-01 | 0.772558 | 0.377-1.582 | 4.81E-01 | 1.40E-03 | 6.16E-01 | 1.040967 | 7.44E-01 | 0 |
| Cheese intake | 0.978418 | 0.836-1.146 | 7.86E-01 | 0.930345 | 0.634-1.365 | 7.13E-01 | 6.32E-04 | 8.27E-01 | 0.924786 | 2.34E-01 | 0 |
| Water intake | 0.89241 | 0.725-1.099 | 2.84E-01 | 0.729492 | 0.482-1.104 | 1.38E-01 | 2.97E-03 | 2.44E-01 | 1.130417 | 2.06E-01 | 0 |
| Cereal intake | 1.139359 | 0.919-1.412 | 2.33E-01 | 0.795511 | 0.477-1.327 | 3.82E-01 | 3.86E-03 | 2.00E-01 | 1.003206 | 9.77E-01 | 0 |
| Dried fruit intake | 1.180528 | 0.939-1.483 | 1.54E-01 | 0.746806 | 0.402-1.387 | 3.57E-01 | 4.66E-03 | 1.63E-01 | 1.045913 | 6.61E-01 | 0 |
| Alcohol usually taken with meals | 0.992634 | 0.729-1.352 | 9.63E-01 | 1.15331 | 0.443-3.005 | 7.71E-01 | -4.17E-04 | 9.13E-01 | 0.993019 | 9.35E-01 | 0 |
| Average weekly spirits intake | 0.874519 | 0.616-1.241 | 4.53E-01 | 1.000901 | 0.474-2.112 | 9.98E-01 | -6.69E-04 | 9.00E-01 | 1.018441 | 8.20E-01 | 0 |
| Non-oily fish intake | 0.937959 | 0.656-1.342 | 7.26E-01 | 1.468546 | 0.720-2.997 | 2.96E-01 | -4.11E-03 | 2.98E-01 | 1.05831 | 6.59E-01 | 0 |
| Salad / raw vegetable intake | 1.046 | 0.735-1.489 | 8.03E-01 | 0.958895 | 0.435-2.112 | 9.17E-01 | 7.82E-04 | 8.31E-01 | 1.260356 | 1.23E-01 | 0 |
| Oily fish intake | 0.96514 | 0.792-1.175 | 7.24E-01 | 0.765427 | 0.473-1.238 | 2.77E-01 | 2.49E-03 | 4.25E-01 | 0.97441 | 6.65E-01 | 0 |
| Beef intake | 1.212767 | 0.907-1.621 | 1.93E-01 | 1.050488 | 0.554-1.992 | 8.80E-01 | 8.52E-04 | 8.13E-01 | 0.926157 | 3.04E-01 | 0 |
| Fresh fruit intake | 1.030106 | 0.752-1.411 | 8.53E-01 | 0.824442 | 0.404-1.681 | 5.96E-01 | 1.66E-03 | 5.73E-01 | 0.905039 | 3.51E-01 | 0 |
| Average weekly beer plus cider intake | 1.087574 | 0.816-1.45 | 5.67E-01 | 2.028612 | 1.115-3.69 | 2.27E-02 | -7.69E-03 | 2.40E-02 | 0.919036 | 4.38E-01 | 0 |
| Coffee intake | 0.903742 | 0.685-1.193 | 4.75E-01 | 1.014819 | 0.714-1.442 | 9.35E-01 | -3.11E-04 | 8.91E-01 | 1.001557 | 9.77E-01 | 0 |
| Average weekly red wine intake | 1.030158 | 0.815-1.302 | 8.03E-01 | 1.287693 | 0.774-2.141 | 3.33E-01 | -3.31E-03 | 3.42E-01 | 1.094322 | 2.45E-01 | 0 |
| pork intake | 1.029333 | 0.697-1.52 | 8.84E-01 | 0.711063 | 0.288-1.753 | 4.62E-01 | 3.73E-03 | 3.69E-01 | 1.099482 | 3.82E-01 | 0 |
| Average weekly champagne plus white wine intake | 1.181555 | 0.809-1.725 | 3.88E-01 | 1.633852 | 0.58-4.604 | 3.58E-01 | -3.31E-03 | 6.12E-01 | 0.922413 | 4.09E-01 | 0 |
| Tea intake | 0.954616 | 0.78-1.168 | 6.52E-01 | 1.125661 | 0.841-1.507 | 4.28E-01 | -2.37E-03 | 2.91E-01 | 0.920874 | 2.51E-01 | 0 |
| Processed meat intake | 0.945797 | 0.773-1.157 | 5.87E-01 | 0.710658 | 0.423-1.194 | 2.00E-01 | 3.63E-03 | 3.00E-01 | 1.09706 | 2.20E-01 | 0 |
| Poultry intake | 0.931954 | 0.678-1.281 | 6.64E-01 | 0.755408 | 0.365-1.566 | 4.53E-01 | 2.13E-03 | 6.12E-01 | 1.145657 | 1.02E-01 | 0 |
| Cooked vegetable intake | 1.068266 | 0.774-1.475 | 6.88E-01 | 0.92962 | 0.391-2.21 | 8.69E-01 | -1.15E-04 | 9.79E-01 | 1.094652 | 4.04E-01 | 0 |
| Salt added to food | 0.910541 | 0.765-1.084 | 2.93E-01 | 1.185936 | 0.825-1.704 | 3.58E-01 | -2.16E-03 | 3.39E-01 | 1.026337 | 8.17E-01 | 0 |
| Milk type used: Full cream | 2.168629 | 0.387-12.166 | 3.79E-01 | 1.672786 | 0.041-68.80 | 7.89E-01 | 3.44E-03 | 6.42E-01 | 3.906263 | 2.54E-02 | 0 |
| Milk type used: Semi-skimmed | 0.555073 | 0.147-2.092 | 3.85E-01 | 0.483116 | 0.031-7.470 | 6.14E-01 | 4.89E-03 | 6.30E-01 | 0.917059 | 8.71E-01 | 0 |
| Milk type used: Skimmed | 0.423285 | 0.167-1.07 | 6.93E-02 | 0.193435 | 0.026-1.418 | 1.21E-01 | 5.22E-03 | 4.20E-01 | 0.424855 | 1.53E-02 | 0 |
| Milk type used: Soya | 2.086501 | 0.163-26.647 | 5.71E-01 | 1.800185 | 0.009-354.5 | 8.30E-01 | 1.77E-03 | 8.37E-01 | 3.034799 | 2.74E-01 | 0 |
| Milk type used: Other type of milk | 0.078539 | 0.0003-23.94 | 3.83E-01 | 4.816043 | 0.004-6434 | 6.79E-01 | -6.35E-03 | 5.05E-01 | 0.580014 | 7.88E-01 | 0 |
| Milk type used: Never/rarely have milk | 1.493351 | 0.108-20.59 | 7.65E-01 | 1.615805 | 0.008-326.8 | 8.62E-01 | 1.94E-04 | 9.79E-01 | 1.727517 | 5.52E-01 | 0 |
| Bread type: White | 0.830625 | 0.527-1.31 | 4.25E-01 | 1.200294 | 0.285-5.055 | 8.04E-01 | -2.31E-03 | 6.27E-01 | 0.847643 | 3.31E-01 | 0 |
| Bread type: Brown | 0.356566 | 0.066-1.932 | 2.32E-01 | 1.095633 | 0.054-22.03 | 9.53E-01 | -4.06E-03 | 6.27E-01 | 0.54507 | 3.27E-01 | 0 |
| Bread type: Wholemeal or wholegrain | 0.755766 | 0.46-1.242 | 2.69E-01 | 0.682049 | 0.134-3.480 | 6.47E-01 | 2.24E-03 | 7.05E-01 | 0.928534 | 6.83E-01 | 0 |
| Bread type: Other type of bread | 0.942324 | 0.068-13.055 | 9.65E-01 | 1.670283 | 0.005-565.5 | 8.65E-01 | -4.46E-03 | 6.73E-01 | 0.508311 | 5.40E-01 | 0 |
| Cereal type: Bran cereal (e.g. All Bran, Branflakes) | 3.00605 | 0.678-13.326 | 1.47E-01 | 2.584777 | 0.181-36.92 | 5.00E-01 | 1.65E-04 | 9.85E-01 | 2.646535 | 9.43E-02 | 0 |
| Cereal type: Biscuit cereal (e.g. Weetabix) | 0.575897 | 0.229-1.451 | 2.42E-01 | 0.582816 | 0.112-3.03 | 5.27E-01 | 4.46E-03 | 4.56E-01 | 1.043869 | 8.84E-01 | 0 |
| Cereal type: Oat cereal (e.g. Ready Brek, porridge) | 1.296397 | 0.45-3.737 | 6.31E-01 | 0.440542 | 0.054-3.603 | 4.56E-01 | 9.56E-03 | 2.87E-01 | 1.277409 | 6.10E-01 | 0 |
| Cereal type: Muesli | 1.247065 | 0.68-2.287 | 4.76E-01 | 0.664163 | 0.19-2.316 | 5.24E-01 | 4.24E-03 | 3.14E-01 | 1.219822 | 2.68E-01 | 0 |
| Cereal type: Other (e.g. Cornflakes, Frosties) | 0.782638 | 0.388-1.578 | 4.93E-01 | 1.468136 | 0.238-9.038 | 6.81E-01 | -5.07E-03 | 4.06E-01 | 0.70037 | 2.15E-01 | 0 |
| Coffee type: Decaffeinated coffee (any type) | 1.226113 | 0.4-3.762 | 7.22E-01 | 0.173426 | 0.024-1.255 | 1.05E-01 | 1.45E-02 | 1.17E-01 | 0.810707 | 6.36E-01 | 0 |
| Coffee type: Instant coffee | 1.172565 | 0.612-2.245 | 6.31E-01 | 0.617692 | 0.113-3.389 | 5.83E-01 | 4.39E-03 | 5.43E-01 | 1.029041 | 9.13E-01 | 0 |
| Coffee type: Ground coffee (include espresso, filter etc) | 1.116209 | 0.708-1.761 | 6.36E-01 | 0.83983 | 0.252-2.793 | 7.77E-01 | 2.44E-03 | 5.72E-01 | 1.17115 | 3.77E-01 | 0 |
| Coffee type: Other type of coffee | 0.002116 | 4.5E-06-1.005 | 5.02E-02 | 0.004018 | 1.3E-09-1.2E+04 | 5.22E-01 | 4.03E-03 | 9.22E-01 | 0.008545 | 1.19E-01 | 0 |

**Table S26.** FDR results of associations between dietary habits and the types of cancer in the research.

| **Exposure** | **BC** | **BC(ER+)** | **BC(ER-)** | **EC** | **EC(EH)** | **EC(NEH)** | **LC** | **LUAD** | **LUSC** | **OC** | **OC(HGS)** | **OC(LGS)** | **OC(ED)** | **OC(CC)** | **OC(IM)** | **PC** |
| --- | --- | --- | --- | --- | --- | --- | --- | --- | --- | --- | --- | --- | --- | --- | --- | --- |
| Age when last ate meat | 4.15E-01 | 8.73E-01 | 2.84E-02 | 9.32E-01 | 8.71E-01 | 9.52E-01 | 9.58E-01 | 9.74E-01 | 9.98E-01 | 9.42E-01 | 9.25E-01 | 9.47E-01 | 9.74E-01 | 9.07E-01 | 7.51E-01 | 9.03E-01 |
| Average weekly fortified wine intake | 7.30E-01 | 8.73E-01 | 7.23E-01 | 4.29E-01 | 2.17E-01 | 9.52E-01 | 8.73E-01 | 9.74E-01 | 9.98E-01 | 8.60E-01 | 8.84E-01 | 9.47E-01 | 9.74E-01 | 9.50E-01 | 8.51E-01 | 8.57E-01 |
| Bread intake | 9.71E-01 | 8.73E-01 | 8.96E-01 | 9.57E-01 | 9.53E-01 | 9.60E-01 | 9.58E-01 | 9.74E-01 | 9.98E-01 | 8.60E-01 | 8.84E-01 | 9.47E-01 | 9.74E-01 | 9.50E-01 | 7.51E-01 | 8.35E-01 |
| lamb/mutton intake | 7.30E-01 | 8.73E-01 | 9.02E-01 | 7.56E-01 | 9.21E-01 | 9.52E-01 | 9.94E-01 | 9.74E-01 | 9.98E-01 | 8.60E-01 | 8.07E-01 | 9.47E-01 | 9.74E-01 | 9.07E-01 | 7.51E-01 | 8.35E-01 |
| Hot drink temperature | 9.71E-01 | 8.73E-01 | 4.15E-01 | 9.57E-01 | 9.98E-01 | 9.52E-01 | 8.48E-01 | 9.74E-01 | 9.98E-01 | 9.42E-01 | 8.84E-01 | 9.47E-01 | 9.74E-01 | 9.07E-01 | 8.46E-01 | 8.35E-01 |
| Cheese intake | 2.31E-01 | 5.86E-01 | 3.02E-02 | 1.08E-01 | 1.85E-01 | 9.52E-01 | 9.58E-01 | 9.74E-01 | 9.98E-01 | 7.80E-01 | 8.07E-01 | 9.47E-01 | 9.74E-01 | 9.07E-01 | 7.51E-01 | 9.03E-01 |
| Water intake | 7.30E-01 | 8.73E-01 | 6.27E-01 | 9.57E-01 | 9.98E-01 | 9.52E-01 | 9.58E-01 | 9.74E-01 | 9.98E-01 | 9.42E-01 | 8.84E-01 | 9.66E-01 | 9.74E-01 | 9.07E-01 | 7.51E-01 | 8.35E-01 |
| Cereal intake | 7.30E-01 | 9.34E-01 | 6.49E-02 | 4.29E-01 | 4.44E-01 | 9.52E-01 | 8.48E-01 | 9.74E-01 | 9.98E-01 | 8.60E-01 | 6.39E-01 | 9.47E-01 | 9.74E-01 | 9.07E-01 | 7.51E-01 | 8.35E-01 |
| Dried fruit intake | 2.31E-01 | 5.86E-01 | 6.49E-02 | 9.57E-01 | 9.98E-01 | 9.52E-01 | 6.35E-01 | 9.74E-01 | 9.98E-01 | 8.60E-01 | 5.97E-01 | 9.47E-01 | 9.74E-01 | 9.07E-01 | 7.51E-01 | 8.35E-01 |
| Alcohol usually taken with meals | 7.30E-01 | 8.73E-01 | 3.56E-01 | 9.57E-01 | 9.98E-01 | 9.52E-01 | 2.58E-02 | 9.74E-01 | 9.27E-01 | 9.58E-01 | 8.84E-01 | 9.47E-01 | 9.74E-01 | 9.07E-01 | 7.51E-01 | 8.35E-01 |
| Average weekly spirits intake | 4.15E-01 | 7.27E-01 | 3.56E-01 | 9.57E-01 | 9.84E-01 | 9.47E-01 | 9.58E-01 | 9.74E-01 | 9.98E-01 | 9.42E-01 | 8.84E-01 | 9.66E-01 | 9.74E-01 | 9.07E-01 | 9.51E-01 | 9.21E-01 |
| Non-oily fish intake | 9.71E-01 | 8.73E-01 | 6.38E-01 | 1.08E-01 | 4.14E-01 | 9.52E-01 | 9.58E-01 | 9.74E-01 | 9.98E-01 | 7.80E-01 | 5.97E-01 | 9.47E-01 | 9.74E-01 | 9.07E-01 | 9.51E-01 | 9.67E-01 |
| Salad / raw vegetable intake | 7.30E-01 | 8.73E-01 | 8.96E-01 | 9.57E-01 | 9.98E-01 | 9.60E-01 | 9.13E-01 | 9.74E-01 | 9.98E-01 | 8.60E-01 | 8.84E-01 | 9.47E-01 | 9.74E-01 | 9.07E-01 | 7.51E-01 | 9.28E-01 |
| Oily fish intake | 4.15E-01 | 8.73E-01 | 8.96E-01 | 9.57E-01 | 9.98E-01 | 9.59E-01 | 6.92E-01 | 9.74E-01 | 9.98E-01 | 8.60E-01 | 8.84E-01 | 9.47E-01 | 9.74E-01 | 9.07E-01 | 7.95E-01 | 8.35E-01 |
| Beef intake | 5.99E-01 | 6.64E-01 | 9.02E-01 | 9.57E-01 | 9.98E-01 | 9.52E-01 | 9.58E-01 | 9.74E-01 | 9.98E-01 | 7.80E-01 | 5.97E-01 | 9.47E-01 | 4.18E-01 | 9.07E-01 | 8.86E-01 | 8.35E-01 |
| Fresh fruit intake | 2.31E-01 | 5.86E-01 | 5.12E-01 | 7.05E-01 | 5.15E-01 | 9.52E-01 | 9.58E-01 | 9.74E-01 | 9.98E-01 | 9.42E-01 | 8.07E-01 | 9.47E-01 | 9.74E-01 | 9.07E-01 | 8.46E-01 | 9.78E-01 |
| Average weekly beer plus cider intake | 8.82E-01 | 8.73E-01 | 9.02E-01 | 9.57E-01 | 9.98E-01 | 9.52E-01 | 4.08E-01 | 9.74E-01 | 9.98E-01 | 9.42E-01 | 8.84E-01 | 9.47E-01 | 9.74E-01 | 9.02E-01 | 7.51E-01 | 9.03E-01 |
| Coffee intake | 7.30E-01 | 7.27E-01 | 2.77E-01 | 5.67E-01 | 9.98E-01 | 9.52E-01 | 8.48E-01 | 9.74E-01 | 9.98E-01 | 9.60E-01 | 9.25E-01 | 9.47E-01 | 9.74E-01 | 9.07E-01 | 7.51E-01 | 9.78E-01 |
| Average weekly red wine intake | 9.71E-01 | 9.11E-01 | 2.77E-01 | 4.29E-01 | 9.98E-01 | 9.52E-01 | 7.95E-01 | 9.74E-01 | 9.98E-01 | 7.80E-01 | 5.97E-01 | 9.47E-01 | 9.74E-01 | 9.07E-01 | 7.51E-01 | 9.67E-01 |
| pork intake | 9.71E-01 | 8.73E-01 | 9.02E-01 | 9.60E-01 | 9.84E-01 | 9.59E-01 | 9.58E-01 | 9.74E-01 | 9.98E-01 | 8.60E-01 | 5.97E-01 | 9.47E-01 | 9.74E-01 | 9.07E-01 | 7.51E-01 | 9.03E-01 |
| Average weekly champagne plus white wine intake | 9.71E-01 | 8.73E-01 | 3.53E-01 | 4.29E-01 | 4.14E-01 | 9.52E-01 | 9.58E-01 | 9.74E-01 | 9.98E-01 | 8.60E-01 | 8.07E-01 | 9.47E-01 | 9.74E-01 | 9.07E-01 | 7.51E-01 | 8.35E-01 |
| Tea intake | 9.00E-01 | 9.15E-01 | 9.02E-01 | 9.32E-01 | 9.21E-01 | 9.52E-01 | 9.95E-01 | 9.74E-01 | 9.98E-01 | 9.72E-01 | 8.84E-01 | 9.47E-01 | 9.74E-01 | 9.07E-01 | 9.51E-01 | 9.03E-01 |
| Processed meat intake | 9.71E-01 | 8.73E-01 | 1.02E-01 | 9.57E-01 | 9.21E-01 | 9.52E-01 | 4.08E-01 | 9.74E-01 | 9.98E-01 | 7.80E-01 | 8.46E-01 | 9.47E-01 | 9.74E-01 | 9.07E-01 | 7.51E-01 | 8.35E-01 |
| Poultry intake | 7.37E-01 | 7.27E-01 | 4.90E-01 | 8.12E-01 | 9.98E-01 | 9.52E-01 | 9.58E-01 | 9.74E-01 | 9.98E-01 | 9.42E-01 | 8.74E-01 | 9.47E-01 | 9.74E-01 | 9.07E-01 | 7.51E-01 | 8.35E-01 |
| Cooked vegetable intake | 9.00E-01 | 9.11E-01 | 9.02E-01 | 4.29E-01 | 4.14E-01 | 9.52E-01 | 9.58E-01 | 9.74E-01 | 9.98E-01 | 8.60E-01 | 5.97E-01 | 9.47E-01 | 9.74E-01 | 9.07E-01 | 9.51E-01 | 8.73E-01 |
| Salt added to food | 7.30E-01 | 8.73E-01 | 6.11E-01 | 2.55E-01 | 4.14E-01 | 9.52E-01 | 9.13E-01 | 9.74E-01 | 9.98E-01 | 8.60E-01 | 8.07E-01 | 9.47E-01 | 9.74E-01 | 9.50E-01 | 7.51E-01 | 9.78E-01 |
| Milk type used: Full cream | 9.71E-01 | 9.15E-01 | 6.11E-01 | 9.14E-01 | 9.98E-01 | 9.52E-01 | 6.35E-01 | 9.74E-01 | 9.98E-01 | 9.42E-01 | 8.84E-01 | 9.75E-01 | 9.74E-01 | 9.07E-01 | 8.46E-01 | 5.74E-01 |
| Milk type used: Semi-skimmed | 7.30E-01 | 8.73E-01 | 8.96E-01 | 9.14E-01 | 9.98E-01 | 9.52E-01 | 9.58E-01 | 9.74E-01 | 9.98E-01 | 9.72E-01 | 8.84E-01 | 9.47E-01 | 9.74E-01 | 9.07E-01 | 9.51E-01 | 9.76E-01 |
| Milk type used: Skimmed | 8.31E-01 | 9.15E-01 | 7.08E-01 | 9.57E-01 | 9.98E-01 | 9.52E-01 | 9.58E-01 | 9.74E-01 | 9.98E-01 | 8.60E-01 | 9.25E-01 | 9.47E-01 | 9.74E-01 | 9.02E-01 | 9.51E-01 | 5.74E-01 |
| Milk type used: Soya | 7.30E-01 | 8.73E-01 | 3.56E-01 | 9.60E-01 | 9.98E-01 | 9.47E-01 | 9.58E-01 | 9.74E-01 | 9.98E-01 | 9.42E-01 | 8.84E-01 | 9.47E-01 | 9.74E-01 | 9.50E-01 | 7.51E-01 | 8.35E-01 |
| Milk type used: Other type of milk | 9.71E-01 | 9.11E-01 | 8.65E-01 | 9.61E-01 | 9.98E-01 | 9.52E-01 | 8.48E-01 | 9.74E-01 | 9.98E-01 | 8.60E-01 | 7.15E-01 | 9.47E-01 | 9.74E-01 | 9.07E-01 | 9.51E-01 | 9.61E-01 |
| Milk type used: Never/rarely have milk | 9.71E-01 | 8.73E-01 | 6.11E-01 | 9.57E-01 | 9.84E-01 | 9.52E-01 | 9.58E-01 | 9.74E-01 | 9.98E-01 | 8.60E-01 | 8.84E-01 | 9.47E-01 | 9.74E-01 | 9.07E-01 | 7.51E-01 | 9.03E-01 |
| Bread type: White | 7.30E-01 | 8.73E-01 | 1.02E-01 | 2.85E-01 | 4.59E-01 | 9.52E-01 | 4.78E-01 | 9.74E-01 | 9.98E-01 | 9.95E-01 | 8.88E-01 | 9.47E-01 | 4.18E-01 | 9.07E-01 | 9.51E-01 | 8.35E-01 |
| Bread type: Brown | 7.30E-01 | 5.86E-01 | 1.02E-01 | 9.57E-01 | 9.98E-01 | 9.60E-01 | 9.58E-01 | 9.74E-01 | 9.98E-01 | 9.72E-01 | 8.07E-01 | 9.47E-01 | 9.74E-01 | 9.07E-01 | 7.51E-01 | 8.35E-01 |
| Bread type: Wholemeal or wholegrain | 5.99E-01 | 7.94E-01 | 6.49E-02 | 9.32E-01 | 9.98E-01 | 9.52E-01 | 8.73E-01 | 9.74E-01 | 9.98E-01 | 8.60E-01 | 5.97E-01 | 9.47E-01 | 9.74E-01 | 9.07E-01 | 7.51E-01 | 9.03E-01 |
| Bread type: Other type of bread | 9.71E-01 | 9.15E-01 | 9.61E-01 | 1.08E-01 | 2.17E-01 | 9.60E-01 | 9.58E-01 | 9.74E-01 | 9.98E-01 | 8.60E-01 | 5.97E-01 | 9.47E-01 | 9.74E-01 | 9.07E-01 | 7.51E-01 | 9.03E-01 |
| Cereal type: Bran cereal (e.g. All Bran, Branflakes) | 7.30E-01 | 5.86E-01 | 3.56E-01 | 4.29E-01 | 5.69E-01 | 9.52E-01 | 9.58E-01 | 9.74E-01 | 9.98E-01 | 9.42E-01 | 9.53E-01 | 9.47E-01 | 9.74E-01 | 9.07E-01 | 9.66E-01 | 7.55E-01 |
| Cereal type: Biscuit cereal (e.g. Weetabix) | 9.71E-01 | 9.34E-01 | 9.66E-01 | 9.57E-01 | 9.98E-01 | 9.52E-01 | 9.58E-01 | 9.74E-01 | 9.98E-01 | 9.59E-01 | 9.25E-01 | 9.47E-01 | 9.74E-01 | 9.50E-01 | 8.51E-01 | 9.77E-01 |
| Cereal type: Oat cereal (e.g. Ready Brek, porridge) | 9.71E-01 | 8.73E-01 | 6.11E-01 | 4.29E-01 | 5.15E-01 | 9.52E-01 | 8.73E-01 | 9.74E-01 | 9.98E-01 | 9.42E-01 | 8.84E-01 | 9.47E-01 | 9.74E-01 | 9.07E-01 | 9.51E-01 | 9.03E-01 |
| Cereal type: Muesli | 9.71E-01 | 8.73E-01 | 8.00E-01 | 4.29E-01 | 3.46E-01 | 9.52E-01 | 9.58E-01 | 9.74E-01 | 9.98E-01 | 9.47E-01 | 9.25E-01 | 9.47E-01 | 9.74E-01 | 9.07E-01 | 7.51E-01 | 8.35E-01 |
| Cereal type: Other (e.g. Cornflakes, Frosties) | 9.71E-01 | 7.41E-01 | 8.96E-01 | 9.57E-01 | 9.84E-01 | 9.52E-01 | 6.35E-01 | 9.74E-01 | 9.27E-01 | 9.42E-01 | 8.07E-01 | 9.47E-01 | 9.74E-01 | 9.02E-01 | 9.51E-01 | 8.35E-01 |
| Coffee type: Decaffeinated coffee (any type) | 2.31E-01 | 6.64E-01 | 6.38E-01 | 9.57E-01 | 9.98E-01 | 9.52E-01 | 9.58E-01 | 9.74E-01 | 9.98E-01 | 8.60E-01 | 6.39E-01 | 9.47E-01 | 9.74E-01 | 9.50E-01 | 7.51E-01 | 9.03E-01 |
| Coffee type: Instant coffee | 9.71E-01 | 8.73E-01 | 2.72E-01 | 4.29E-01 | 9.84E-01 | 9.52E-01 | 9.58E-01 | 9.74E-01 | 9.98E-01 | 9.72E-01 | 8.88E-01 | 9.47E-01 | 9.74E-01 | 9.50E-01 | 7.51E-01 | 9.77E-01 |
| Coffee type: Ground coffee (include espresso, filter etc) | 7.30E-01 | 8.73E-01 | 6.49E-02 | 1.08E-01 | 1.85E-01 | 9.47E-01 | 6.92E-01 | 9.74E-01 | 9.98E-01 | 8.60E-01 | 5.97E-01 | 9.47E-01 | 9.74E-01 | 9.07E-01 | 9.66E-01 | 8.35E-01 |
| Coffee type: Other type of coffee | 9.00E-01 | 8.73E-01 | 6.11E-01 | 9.57E-01 | 9.53E-01 | 9.60E-01 | 5.60E-01 | 9.74E-01 | 9.98E-01 | 9.72E-01 | 8.84E-01 | 9.47E-01 | 9.74E-01 | 9.07E-01 | 7.51E-01 | 7.18E-01 |

**Table S27.** MVMR analysis in reliable causality evidence.

| **Exposure** | **Outcome** | **Model** | **nSNPs** | **Methods** | | |
| --- | --- | --- | --- | --- | --- | --- |
|  |  |  |  | **P(IVW)** | **Egger Intercept** | **Egger Int.p*** |
| BC(R+) | Bran cereal | Body mass index | 160 | 0.306056 | -2.01E-03 | 1.85E-01 |
| BC(R+) | Bran cereal | Age at menopause | 89 | 0.515832 | -2.00E-04 | 9.26E-01 |
| BC(R+) | Bran cereal | Age at menarche | 131 | 0.482532 | -3.30E-04 | 8.48E-01 |
| BC(R+) | Bran cereal | Age Of Smoking Initiation | 59 | 0.858157 | -9.60E-04 | 6.67E-01 |
| OC(HGS) | Non-oily fish intake | Body mass index | 158 | 0.635154 | -2.89E-03 | 1.85E-01 |
| OC(HGS) | Non-oily fish intake | Age at menopause | 90 | 0.560008 | 3.67E-04 | 9.02E-01 |
| OC(HGS) | Non-oily fish intake | Age at menarche | 138 | 0.089974 | 1.31E-03 | 5.66E-01 |
| OC(HGS) | Non-oily fish intake | Age Of Smoking Initiation | 96 | 0.00767 | -2.93E-03 | 4.17E-01 |
| LC | Average weekly beer plus cider intake | Body mass index | 172 | 4.78E-05 | 6.97E-04 | 7.87E-01 |
| LC | Average weekly beer plus cider intake | Age Of Smoking Initiation | 124 | 8.00E-06 | -4.38E-03 | 2.79E-01 |
| LC | Processed meat intake | Body mass index | 172 | 0.07054 | -1.31E-03 | 6.17E-01 |
| LC | Processed meat intake | Age Of Smoking Initiation | 136 | 0.237363 | 1.38E-03 | 7.71E-01 |

**Table S28.** The effect of dietary habits on overall survival and cancer-specific survival in common cancers.

| SNPS | Exposure | Outcome | HR_OS | SE-OS | P_value_OS | HR_CSS | SE-CSS | P_value_CSS |
| --- | --- | --- | --- | --- | --- | --- | --- | --- |
| rs9824301 | Average weekly beer plus cider intake | LC | 0.91 | 0.04 | 0.047 | 0.04 | 0.04 | 0.064 |
| rs34895146 | Average weekly beer plus cider intake | LC | 1.29 | 0.10 | 0.014 | 1.20 | 0.11 | 0.109 |
| rs1283208 | Average weekly beer plus cider intake | LC | 0.91 | 0.04 | 0.033 | 0.88 | 0.04 | 0.006 |
| rs35782576 | Average weekly beer plus cider intake | LC | 1.10 | 0.04 | 0.042 | 1.12 | 0.05 | 0.053 |
